# E2F and STAT3 provide transcriptional synergy for histone variant H2AZ activation to sustain glioblastoma chromatin accessibility and tumorigenicity

**DOI:** 10.1038/s41418-021-00926-5

**Published:** 2022-01-20

**Authors:** Jeehyun Yoon, Oleg V. Grinchuk, Roberto Tirado-Magallanes, Zhen Kai Ngian, Emmy Xue Yun Tay, You Heng Chuah, Bernice Woon Li Lee, Jia Feng, Karen Carmelina Crasta, Chin Tong Ong, Touati Benoukraf, Derrick Sek Tong Ong

**Affiliations:** 1 Department of Physiology, Yong Loo Lin School of Medicine, National University of Singapore, Singapore, 117593 Singapore; 2 NUS Center for Cancer Research, Yong Loo Lin School of Medicine, National University of Singapore, Singapore, Singapore; 3 Cancer Science Institute of Singapore, Yong Loo Lin School of Medicine, National University of Singapore, Singapore, 117599 Singapore; 4 Temasek Life Sciences Laboratory, National University of Singapore, Singapore, 117604 Singapore; 5 Centre for Healthy Longevity, National University Health System, Singapore, 119228 Singapore; 6 Institute of Molecular and Cell Biology (IMCB), Agency for Science, Technology and Research (A*STAR), Singapore, Singapore; 7 Department of Biological Sciences, National University of Singapore, Singapore, 117558 Singapore; 8 Division of BioMedical Sciences, Faculty of Medicine, Memorial University of Newfoundland, St. John’s, NL A1B 3V6 Canada; 9 National Neuroscience Institute, Singapore, 308433 Singapore

**Keywords:** Cancer stem cells, Gene regulation

## Abstract

The histone variant H2AZ is overexpressed in diverse cancer types where it facilitates the accessibility of transcriptional regulators to the promoters of cell cycle genes. However, the molecular basis for its dysregulation in cancer remains unknown. Here, we report that glioblastomas (GBM) and glioma stem cells (GSCs) preferentially overexpress *H2AZ* for their proliferation, stemness and tumorigenicity. Chromatin accessibility analysis of *H2AZ2* depleted GSC revealed that E2F1 occupies the enhancer region within *H2AZ2* gene promoter, thereby activating *H2AZ2* transcription. Exploration of other *H2AZ2* transcriptional activators using a customized “anti-*H2AZ2*” query signature for connectivity map analysis identified STAT3. Co-targeting E2F and STAT3 synergistically reduced the levels of H2AZ, histone 3 lysine 27 acetylation (H3K27ac) and cell cycle gene transcription, indicating that E2F1 and STAT3 synergize to activate *H2AZ* gene transcription in GSCs. Remarkably, an E2F/STAT3 inhibitor combination durably suppresses GSC tumorigenicity in an orthotopic GBM xenograft model. In glioma patients, high STAT3 signaling is associated with high *E2F1* and *H2AZ2* expression. Thus, GBM has uniquely opted the use of E2F1- and STAT3-containing “enhanceosomes” that integrate multiple signaling pathways to achieve *H2AZ* gene activation, supporting a translational path for the E2F/STAT3 inhibitor combination to be applied in GBM treatment.

## Introduction

Despite our improved molecular understanding of glioblastomas (GBM), maximal surgical resection followed by radiotherapy and adjuvant Temozolomide treatment remains the standard-of-care for this disease. Tumor recurrence is unfortunately inevitable, highlighting an urgent need to uncover more durable treatment options for GBM. A subset of GBM cells, commonly referred to as glioma stem cells (GSCs), exhibit stem-like traits, robust proliferative and invasive capacity, as well as therapy resistance, hence is widely adopted as an invaluable experimental GBM model [1–5]. GBM exploits various epigenetic aberrancies, including widespread changes in DNA methylation, redistribution of histone marks and interference in chromatin structure, to regulate its cell state and differentiation programs [6–10]. We have also recently reported that GBM is dependent on biotin distribution to carboxylases and histones, in order to sustain its specific metabolic and epigenetic requirement for proliferation and invasiveness [11].

Notably, two distinct isoforms of H2AZ variant histone, namely H2AZ1 and H2AZ2 that are encoded by two non-allelic genes and differ only by three amino acids, are overexpressed in multiple cancers [12–15]. Like their canonical counterparts, the main function of variant histone proteins is to generate nucleosomes for genome organization within the nucleus. However, the variant histones differ from the canonical histones in their unique temporal expression patterns and genomic loci-specific deposition. As a result, they play a key role in controlling gene transcription by directly affecting nucleosome structure and stability or indirectly affecting chromatin organization via their posttranslational modifications, thereby influencing organismal development and tumorigenesis [16, 17]. While much is established about the chromatin occupancy (e.g., mainly at gene promoters and enhancers), deposition mechanisms (e.g., by p400 and SWI2–SNF2-related CBP activator protein (SRCAP)) and posttranslational modification (e.g., acetylation) of H2AZ, as well as its effect on chromatin structure and gene transcription (e.g., destabilizes nucleosomes and facilitates gene transcription), how H2AZ is dysregulated in cancer remains poorly understood [14, 17–23]. Whether H2AZ levels influence GSC stemness and GBM progression have also not been explored.

Genome sequencing studies of GBM have illuminated biologically relevant alterations in three core pathways, namely MDM2/p53, Rb/E2F, and receptor tyrosine kinase (RTK)/Ras/phosphoinositide 3-kinase signaling, which endow GBM with unlimited proliferative capacity [24, 25]. Given that histone protein synthesis is required for cell proliferation, these dysregulated pathways may exert a prominent role in histone gene regulation in GBM. Early studies demonstrated a crucial role of H2AZ in cell cycle progression and chromosome segregation, hinting that the transcription of H2AZ, unlike that of other variant histones, may be coupled to cell cycle progression [26, 27]. In addition, E2F1 regulates cell cycle gene transcription in a H2AZ- and BET bromodomain protein-dependent manner in melanoma and GBM [14, 17, 28]. There also appears to be a connection between chromatin accessibility at E2F target genes and cell identity as it decreases when wing and pluripotent stem cells differentiate and exit from cell cycle [29, 30]. Collectively, these observations raise the possibility that E2Fs may regulate *H2AZ* gene transcription, which in turn affect cell proliferation and stemness of GSC. Whether activation of other GBM signaling pathways, including STAT3 that is downstream of RTK/JAK signaling, can direct *H2AZ* transcription in GSC is also unclear. Such molecular insights are important as they may offer new therapeutic opportunities for GBM.

In this study, we report that E2F1 and STAT3 synergistically activate *H2AZ* transcription in GSC by employing unbiased epigenomic profiling and chemical biology approaches to identify candidate transcriptional regulators. Our transcriptional analyses of *H2AZ* led to the rational combination of E2F and STAT3 inhibitors as a potential anti-GSC therapy, which synergistically reduced the levels of H2AZ, chromatin accessibility and cell cycle gene expression. Intriguingly, a short-term treatment of GSC with an E2F/STAT3 inhibitor combination durably impaired GSC tumorigenicity in an orthotopic GBM xenograft model. Finally, we highlight the intimate link between high STAT3 signaling, *E2F1* and *H2AZ2* expression in glioma patients. Thus, our findings suggest that an E2F/STAT3 inhibitor combination may be further developed for GBM treatment.

## Results

### High expression of the *H2AZ* isoforms correlates with GBM and GSC stemness

**Fig. 1:**
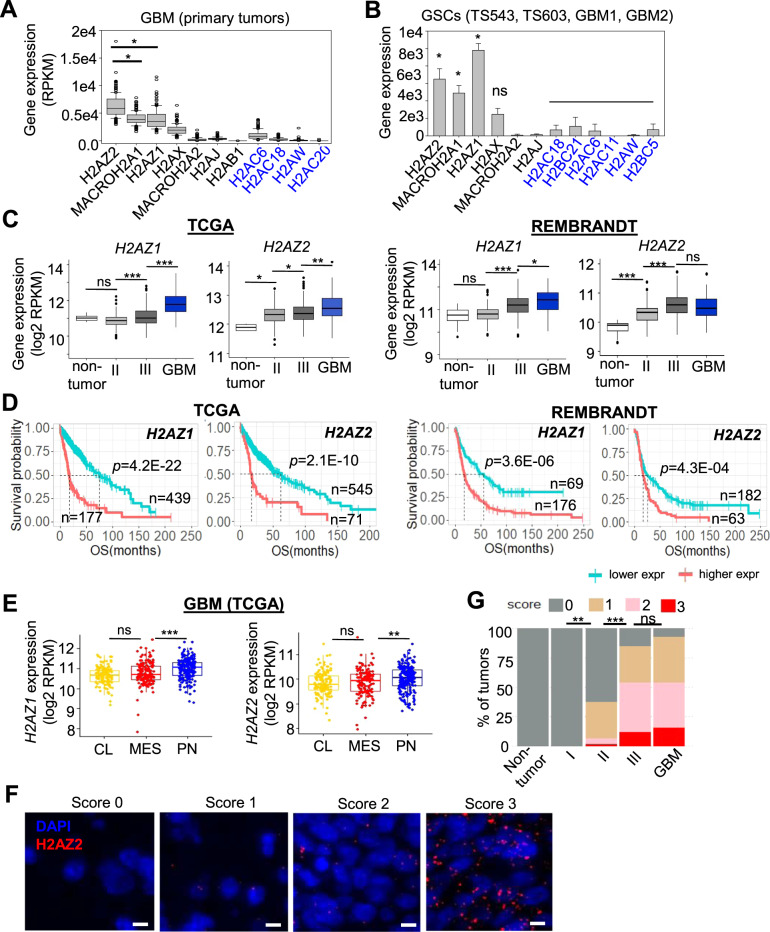
High expression of the *H2AZ* isoforms correlates with GBM and GSC stemness. Gene expression of selected H2A variants and canonical H2A in TCGA GBM (**A**) and GSCs (**B**). Only the top 4 canonical H2A genes (blue font) with the highest expression are shown. **A** *p < 1.0e−20 (Mann–Whitney test); **B** *p < 0.01 (two-tailed unpaired Student’s *t* test) denotes that the given variant H2A gene expression is significantly higher than expression of any of the shown canonical H2A genes. **C** Comparison of *H2AZ* mRNA levels in non-tumors vs. gliomas of different clinical grades in TCGA and REMBRANDT cohorts. Man–Whitney test, *p < 0.05, **p < 1.0e−04, ***p < 1.0e−12. **D** Correlative analysis of *H2AZ* levels with glioma patient survival in multiple glioma patient cohorts. OS overall survival. Wald test. **E** Correlative analysis of *H2AZ* levels with classical (CL), mesenchymal (MES) and proneural (PN) GBM subtypes in TCGA GBM. Man–Whitney test, **p < 0.01, ***p < 0.001. **F** Detection of *H2AZ2* RNA transcripts in tumor cells from glioma patients of different clinical grades using the RNAscope assay. Scale bar, 10 µm. **G** RNAscope scores of *H2AZ2* transcripts in different grades of glioma using glioma patient TMA (from **F**). *H2AZ2* mRNA transcripts counts are binned into three score classes according to the RNAscope ® Assay Semi-Quantative Scoring Guideline. Differences in gene expression are estimated using Fisher-Freeman-Halton exact test. **p < 0.01, ***p < 0.001.

To assess the clinical relevance of the *H2AZ* isoforms in GBM, we first examined their expression in GBM and GSCs. This revealed their preferential overexpression in GBM and GSCs when compared to the canonical *H2A* members, suggesting that the *H2AZ* isoforms may play a critical role in regulating the epigenome of GBM and GSC (Fig. 1A, B). Notably, the expression of the *H2AZ* isoforms was significantly higher in GSCs than serum-induced, differentiated GSCs, strengthening their relation with GSC stemness (Supplementary Fig. S1A). The expression of both *H2AZ* isoforms was also significantly higher in high grade (Grade III and GBM) than low grade gliomas and non-tumors in the TCGA and NCI REMBRANDT datasets (Fig. 1C). Accordingly, we observed a robust association between high *H2AZ* expression and poor glioma patient survival in multiple patient cohorts (Fig. 1D). The expression of both *H2AZ* isoforms was also significantly higher in the proneural than mesenchymal and classical GBM subtypes in the TCGA dataset, consistent with a potential role of H2AZ in GBM stemness that is associated with the proneural subtype (Fig. 1E). Correlative analysis of *H2AZ* levels with various GBM genotypes revealed a significant, positive association with *TP53* mutation and *PDGFRA* amplifications, but not *IDH1* mutation, promoter methylation of *MGMT*, *CDK4*/*CDK6* amplifications and *PTEN* mutation in GBM (Supplementary Fig. S1B–G). Interestingly, only *H2AZ2* (but not *H2AZ1*) expression rigorously correlated with GSCs as (1) its expression was higher in GSCs (CD133^+^ or Nestin^high^) than non-GSCs (CD133^−^ or Nestin^low^) (Supplementary Fig. S1H, I); and its expression was significantly higher in GSCs than bulk GBM tumors (Supplementary Fig. S1J). Given the lack of a H2AZ2-specific antibody to further substantiate the overexpression of *H2AZ2* in GBM, we performed RNAscope analysis of *H2AZ2* in glioma patient tumor microarray wherein each single dot represents a *H2AZ2* transcript at single cell resolution. This confirmed that GBM preferentially expressed higher *H2AZ2* levels than low grade gliomas and non-tumors (Fig. 1F, G). Since the H2AZ1 and H2AZ2 isoforms share many similarities in their genome-wide occupancy and protein interactions [14, 31, 32], we speculate that both isoforms would regulate glioma proliferation. However, we decided to focus on the *H2AZ2* isoform in our transcriptional studies due to its stronger correlation with GSC stemness markers, including Nestin and CD133, than *H2AZ1* from multiple published datasets.

### *H2AZ2* depletion compromises GSC self-renewal/proliferation and tumorigenicity

**Fig. 2:**
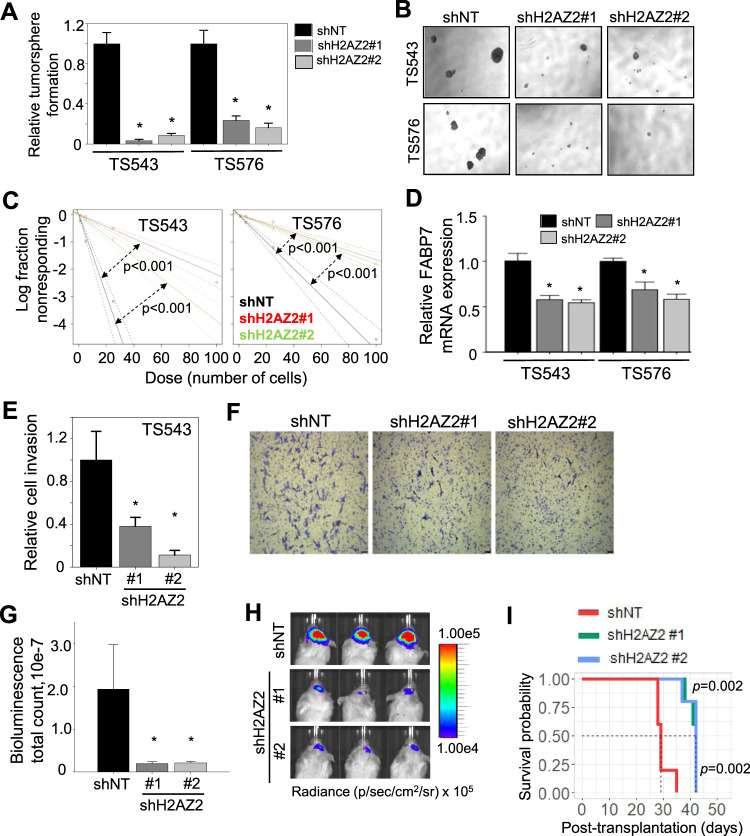
*H2AZ2* depletion compromises GSC self-renewal/proliferation and tumorigenicity. **A**, **B** Tumorsphere formation and representative images of GSCs following *H2AZ2* KD (*N* = 6) (mean ± SD). *p < 1.0e−3. **C** In vitro limiting dilution assays of GSCs transduced with NT/control or H2AZ2 shRNA calculated with ELDA analysis. **D** qRT-PCR analysis of *FABP7* mRNA levels of GSCs with or without *H2AZ2* KD (*N* = 3) (mean ± SD). The housekeeping genes include *TBP*, TATA box binding protein; *HSP70*, Heat shock protein 70; and *ACTB*, beta actin. *p < 0.05. **E** Transwell migration and invasion assay of GSCs with *H2AZ2* KD (*n* = 3) (mean ± SD). *p < 0.05. **F** Representative images of (**E**). **G**, **H** In vivo bioluminescence-based imaging 22 days post-orthotropic injection of GSC TS543 (1 × 10^5^ cells) transduced with NT/control or H2AZ2 shRNAs. Quantification of tumor volume based on bioluminescence (**G**) and representative images of the tumor-bearing mice (**H**) (*n* = 5) (mean ± SD). *p < 0.05. **I** Survival curves of mice implanted with GSC TS543 transduced with NT or H2AZ2 shRNA. **A**, **C**–**E**, **G** Two-tailed unpaired Student’s *t* test; **I** log-rank test.

To evaluate the importance of *H2AZ2* in GSC biology, we employed a myriad of in vitro and in vivo assays, including the tumorsphere assay (a readout for GSC proliferation); extreme limiting dilution assay (a readout for tumor initiating cell frequency); soft agar colony formation assay (a readout for GSC clonogenicity and transforming potential); Transwell migration and invasion assay (a readout for GSC invasiveness); and xenotransplantation assay (a readout for GSC tumorigenicity) [5]. The efficient KD of *H2AZ2* resulted in a dramatic reduction in the number and size of tumorspheres of multiple GSC lines (Supplementary Fig. S2A, B and Fig. 2A, B), indicating reduced GSC proliferation (NB: the pan-H2AZ antibody detects both H2AZ2 and H2AZ1). Silencing *H2AZ2* also significantly decreased GSC tumor initiating cell frequency as revealed by the extreme limiting dilution assay, which tracked with reduced FABP7 (a GSC marker) levels (Fig. 2C, D). This corroborated with a significant reduction of GSC colony formation upon *H2AZ2* depletion (Supplementary Fig. S2C). Furthermore, *H2AZ2* KD significantly impaired GSC invasiveness (Fig. 2E, F) and sensitized GSC to carboplatin-induced apoptosis (Supplementary Fig. S2D, E). Extending these in vitro findings to xenotransplantation experiments, we showed that *H2AZ2* depleted GSCs generated significantly smaller tumor volume than the *H2AZ2* intact controls (Fig. 2G, H). Accordingly, mice bearing *H2AZ2* depleted GSCs survived significantly longer than those bearing *H2AZ2* intact GSCs (median survival of 42 vs. 29 days) (Fig. 2I). Collectively, our data demonstrate that high *H2AZ2* levels are crucial for GSC proliferation, stemness, invasiveness and tumorigenicity.

### Chromatin accessibility analysis unveils E2F1 as a transcriptional activator of *H2AZ2*

The molecular basis for *H2AZ2* overexpression in cancer remains unclear. We hypothesize that the H2AZ2 protein may be deposited on the promoters of GSC-critical genes, including *H2AZ2*, where it controls the access of transcriptional complexes to these genomic regions. To identify direct gene targets of the H2AZ2 protein in GSC, we first performed H2AZ chromatin immunoprecipitation followed by sequencing (ChIP-Seq) analysis (using a pan-H2AZ antibody since H2AZ2 and H2AZ1 showed very similar genome-wide occupancy [14, 32]). Consistent with previous reports, we observed a selective enrichment of H2AZ at the promoters (14.2%), CpG island (3.7%) and 5′ untranslated regions (UTRs) (1.4%) of genes (Supplementary Fig. S3A, B) [14, 32]. Comparison of H2AZ, H3K4me3 (active transcription mark) and H3K27ac (active enhancer mark) ChIP-Seq peaks showed that the H2AZ ChIP-Seq peaks colocalized with that of the H3K27ac, suggesting that H2AZ may regulate the accessibility of transcriptional regulators to enhancer elements within GSC gene promoters (Supplementary Fig. S3C).

**Fig. 3:**
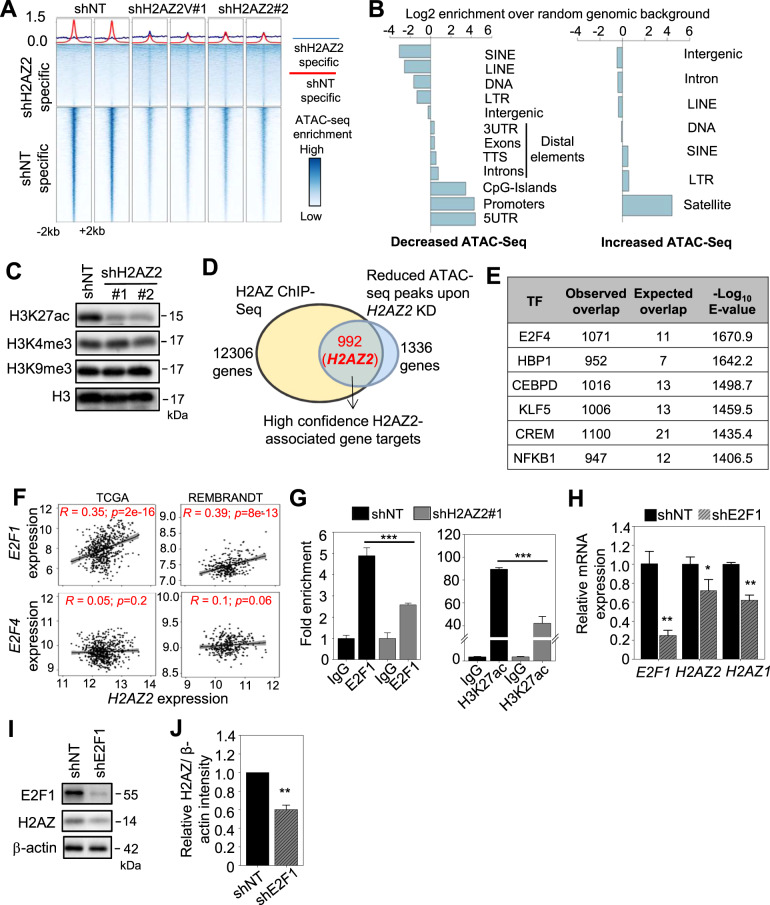
Chromatin accessibility analysis unveils E2F1 as a transcriptional activator of *H2AZ2*. **A** Heatmaps displaying the differences in ATAC-Seq signal after *H2AZ2* KD. The panels on top of the heatmaps show the average signal for each group of regions in each individual sample. **B** Barplot showing the log_2_ enrichment of the observed over expected overlap of genomic regions with differential ATAC-Seq signal upon *H2AZ2* KD as shown in (**A**). **C** Western blot analysis of H3K27ac, H3K4me3, H3K9me3 proteins levels upon *H2AZ2* KD of GSC. Histone H3 serves as the loading control. **D** Venn diagram showing the 992 high confidence H2AZ2-associated gene targets from the intersection between the H2AZ ChIP-Seq and ATAC-Seq analysis upon *H2AZ2* KD. **E** Transcription factor enrichment analysis (ReMap software) within the significantly decreased ATAC-seq peaks upon *H2AZ2* KD. **F** Correlative analysis of *H2AZ2* levels with that of *E2F1* or *E2F4* in glioma patients from TCGA and REMBRANDT cohorts. **G** ChIP-qPCR analysis of E2F1 occupancy and H3K27ac levels on the *H2AZ2* promoter in *H2AZ2* depleted GSC (*n* = 3) (mean ± SD). ***p < 0.005. **H** qRT-PCR analysis of *H2AZ2* and *H2AZ1* mRNA levels in *E2F1* KD GSC. *HSP70* and *TBP* serve as the housekeeping genes (*n* = 3) (mean ± SD). *p < 0.05; **p < 0.005. **I** Western blot analysis of E2F1 and H2AZ protein levels upon *E2F1* KD in GSC. **J** Quantification of H2AZ band intensities in (**I**) when normalized to the β-actin control (*n* = 3) (mean ± SD). **p < 0.005. **G**, **H**, **J** Two-tailed unpaired Student’s *t* test.

Next, the assay for transposase-accessible chromatin using sequencing (ATAC-Seq) analysis of *H2AZ2* depleted vs. intact cells revealed that the majority of the differential ATAC-Seq peaks was significantly reduced (6206 reduced peaks vs. 3346 enhanced peaks) upon *H2AZ2* KD (Fig. 3A). These reduced ATAC-Seq peaks were mapped to gene promoters, CpG islands, 5′ UTRs, as well as distal regions (including 3′ UTRs, exons, introns and transcription termination sites) that were significantly enriched over genomic background control, accounting for 66.4% of the reduced ATAC-Seq peaks upon *H2AZ2* KD, indicating decreased chromatin accessibility mainly at gene promoters in GSC (Fig. 3B and Supplementary Fig. S3D). On the other hand, the enhanced ATAC-Seq peaks were mapped to repetitive elements, including short interspersed nuclear elements, long terminal repeats, and satellite DNA, accounting for 38.21% of the enhanced ATAC-Seq peaks upon *H2AZ2* KD (Fig. 3B and Supplementary Fig. S3D). That *H2AZ2* KD leads to reduced chromatin accessibility at enhancer regions within GSC gene promoters was consistent with a specific decrease in H3K27ac, but not H3K4me3 levels (Fig. 3C).

To uncover genes whose promoter accessibility is directly controlled by the H2AZ2 protein, we intersected our H2AZ ChIP-Seq and ATAC-Seq datasets, and found *H2AZ2* to be one of the high confidence H2AZ2-associated genes that was common in both analyses (Fig. 3D and Supplementary Table S1). Thus, the H2AZ2 protein associates with the promoter region of the *H2AZ2* gene, where it may facilitate the access of transcriptional regulators to the *H2AZ2* gene promoter. Interestingly, our integrative analysis also revealed that while H2AZ2 was deposited on numerous gene promoters, only a fraction of these gene promoters (992 out of 12,306) significantly experienced chromatin compaction upon *H2AZ2* depletion (Fig. 3D). From our ATAC-Seq analysis of *H2AZ2* KD cells, we searched the ReMap datasets and identified E2F4 among the top glioma-relevant transcriptional regulators whose promoter accessibility may be altered in *H2AZ2* depleted cells (Fig. 3E and Supplementary Table S2). The E2F family of transcription factors (TF) regulate S-phase entry and there are at least seven E2F members in mammals. E2F1, E2F2 and E2F3 have potent transcriptional activation activity, interact exclusively with pRb and are expressed periodically during the cell cycle [33]. In contrast, E2F4 and E2F5 are poor transcriptional activators and appear to function as repressors by recruiting pocket proteins to E2F-regulated promoters [33]. Since E2Fs bind to similar DNA binding sequences [34], we conducted correlative analysis of *H2AZ2* with *E2F1* and *E2F4* in TCGA glioma. There was a significant association between *H2AZ2* and *E2F1* (but not *E2F4*), suggesting that *E2F1* may be co-expressed with *H2AZ2* in glioma (Fig. 3F). Integrative analysis of our ATAC-Seq, H3K27ac and H2AZ ChIP-Seq data, together with a published E2F1 ChIP-Seq analysis of the U87 GBM cell line further supported the idea that E2F1 may bind to the enhancer element within *H2AZ2* gene promoter in GSC (Supplementary Fig. S3E) [28]. Indeed, we validated a significant decrease in E2F1 occupancy at the enhancer region within *H2AZ2* gene promoter upon *H2AZ2* depletion, which corresponded with a reduction in the H3K27ac mark (Fig. 3G). Accordingly, *E2F1* KD significantly decreased *H2AZ2* (and *H2AZ1*) mRNA and protein levels in GSC (Fig. 3H–J). Importantly, we showed that *H2AZ2* overexpression can partially rescue the impaired colony formation of *E2F1* KD GSC, indicating that H2AZ2 levels in part contribute to the effect of E2F1 on GSC proliferation (Supplementary Fig. S3F–H). Thus, we conclude that E2F1 activates *H2AZ2* transcription in GSCs.

### E2F inhibition does not fully recapitulate the transcriptomic changes elicited by *H2AZ2* KD

**Fig. 4:**
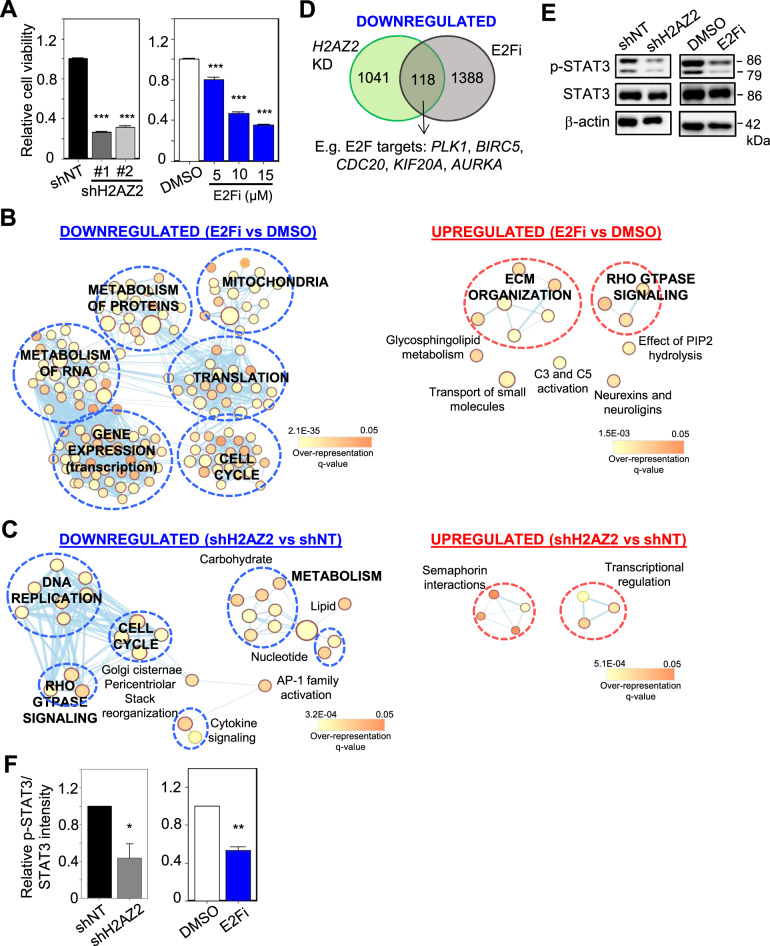
E2F inhibition does not fully recapitulate the transcriptomic changes elicited by *H2AZ2* KD. **A** Cell viability assay of GSCs with *H2AZ2* KD or 3 days of E2Fi treatment (*n* = 6) (mean ± SD). ***p < 0.001. Gene set enrichment map of pathways containing genes downregulated or upregulated upon 15 μM E2Fi (3 days) treatment (**B**) or *H2AZ2* depletion (**C**) of GSC. Nodes represent gene sets (pathways) that were significantly enriched in the comparison treated vs. control samples (FDR < 0.05). **D** Venn diagram showing the number of overlapped genes among the downregulated genes upon 15 μM E2Fi treatment (3 days) or *H2AZ2* depletion. **E** Western blot analysis of p-STAT3 and STAT3 protein levels in *H2AZ2* KD or 15 μM E2Fi-treated GSC. **F** Quantification of p-STAT3 band intensities in (**E**) when normalized to the STAT3 control (*n* = 3) (mean ± SD). *p < 0.05; **p < 0.01. **A**, **F** Two-tailed unpaired Student’s *t* test.

In melanoma, it was proposed that H2AZ2 exerts its oncogenic function by recruiting E2F1 and BRD2 to the promoters of cell cycle genes [14]. Thus, we asked if direct E2F inhibition (to mimic reduced E2F chromatin accessibility upon *H2AZ2* depletion) by using HLM006474, a selective E2F inhibitor (hereafter referred to as E2Fi) [35], is sufficient to elicit the *H2AZ2* KD-associated transcriptomic changes in GSC. First, we demonstrated that 15 μM E2Fi treatment significantly reduced GSC viability similar to that of *H2AZ2* KD (Fig. 4A). RNA-Seq followed by ConsensusPathDB analysis of the differentially expressed genes (DEGs) revealed that while both 15 μM E2Fi treatment and *H2AZ2* KD downregulated the cell cycle pathway, *H2AZ2* depletion also downregulated other GSC-critical pathways, including DNA replication and Rho GTPase signaling (related to cell migration) (Fig. 4B, C). Transcriptomic comparison of the E2Fi-treated vs. *H2AZ2* KD GSC showed that there were about 118 common downregulated genes, including *PLK1*, *BIRC5*, *CDC20*, *KIF20A*, *AURKA* that are well-established E2F targets (Fig. 4D). We validated a subset of cell cycle genes from our RNA-Seq data, including *MELK* (that encodes a serine/threonine-protein kinase that is involved in cell cycle regulation and self-renewal of GSCs [36]) and *PLK1* (that regulates centrosome maturation and spindle assembly, mitotic exit, cytokinesis and survival of GSCs [37]) in *H2AZ2* depleted GSC (Supplementary Fig. S4A). As expected, *H2AZ2* depleted GSCs exhibited a block in G1 → S progression (Supplementary Fig. S4B) and there was a significant reduction in S-phase cells upon *H2AZ2* KD as revealed by transient BrdU labeling analysis (Supplementary Fig. S4C, D). To more precisely determine the downstream consequence of *H2AZ2* KD in GSC, we performed further integrative analyses and found that about 99 high confidence H2AZ2-associated genes with reduced transcriptional output potentially harbor E2F1 and/or STAT3 binding sites (68 E2F1 targets; 2 STAT3 targets; 18 E2F1 and STAT3 targets, including *H2AZ2*) (Supplementary Fig. S4E, F and Supplementary Table S3). Therefore, a direct outcome of *H2AZ2* gene silencing appears to reduce promoter accessibility of E2F1 and STAT3 to their target genes, which influence GSC proliferation and invasiveness. Accordingly, *H2AZ2* KD treatment significantly reduced p-STAT3 levels in GSC (Fig. 4E, F). It is notable that p-STAT3 levels also decreased with 15 μM E2Fi treatment of GSC, consistent with H2AZ downregulation upon *E2F1* KD (Fig. 4E, F and 3I). Collectively, our findings indicate that E2F inhibition does not fully recapitulate the transcriptomic alterations that are elicited by *H2AZ2* KD in GSC.

### A chemical biology approach identified STAT3 as another *H2AZ2* transcriptional activator

**Fig. 5:**
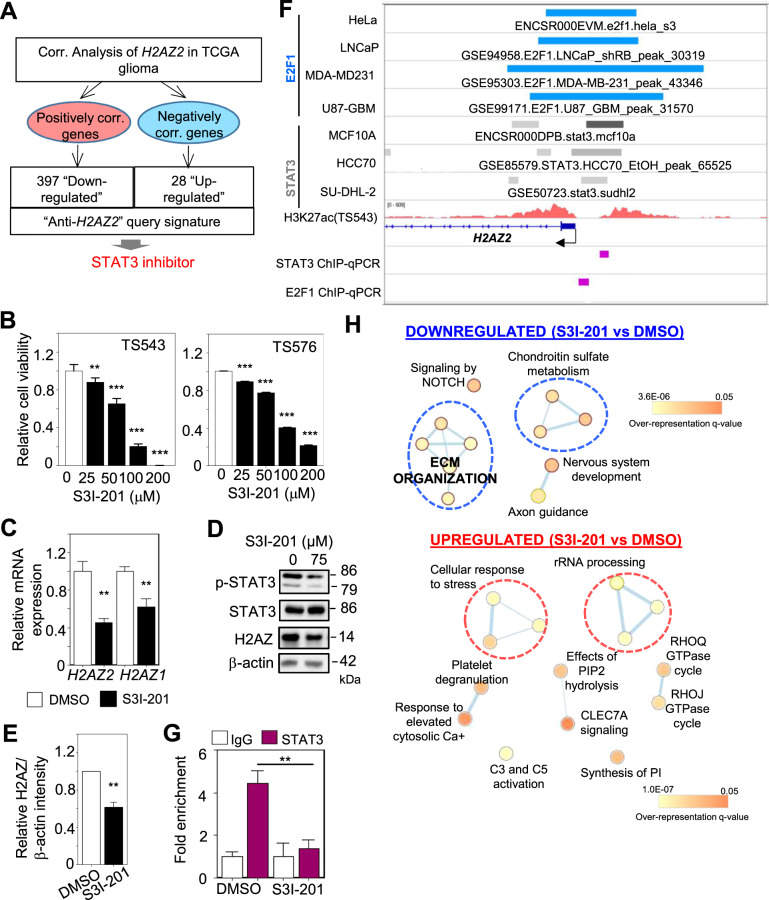
A chemical biology approach identified STAT3 as another *H2AZ2* transcriptional activator. **A** CMA using the “anti-*H2AZ2*” gene signature identified STAT3 inhibitor that may downregulate a subset of *H2AZ2*-associated genes. **B** Cell viability assay of GSCs with 3 days of S3I-201 treatment (*n* = 8) (mean ± SD) **p < 0.01; ***p < 0.001. **C** qRT-PCR analysis of *H2AZ2* and *H2AZ1* mRNA levels upon S3I-201 treatment of GSC. *HSP70* and *TBP* serve as the housekeeping genes (*n* = 3) (mean ± SD). **p < 0.005. **D** Western blot analysis of p-STAT3, STAT3, and H2AZ protein levels upon S3I-201 treatment of GSC. β-actin serves the loading control. **E** Quantification of H2AZ band intensities in (**D**) when normalized to β-actin control (*n* = 3) (mean ± SD). **p < 0.01. **F** E2F1, STAT3, and H3K27ac ChIP-Seq tracks for *H2AZ2* in the indicated cell lines, along with the location of ChIP-qPCR primers. **G** ChIP-qPCR analysis of STAT3 occupancy on the *H2AZ2* promoter upon S3I-201 treatment of GSC (*n* = 3) (mean ± SD). **p < 0.01. **H** Gene set enrichment map of pathways containing genes downregulated or upregulated upon 75 μM S3I-201 treatment of GSC (3 days). Nodes represent gene sets (pathways) that were significantly enriched in the comparison treated vs. control samples (FDR < 0.05). Two-tailed unpaired Student’s *t* test.

Next, we attempted to identify other transcriptional regulators of *H2AZ2* by inferring from compounds that can downregulate *H2AZ2*-associated genes (as a readout of *H2AZ2* level). To this end, we generated a query signature that comprised the low expression of *H2AZ2*-positively correlated genes and high expression of *H2AZ2*-negatively correlated genes in TCGA gliomas, and submitted this “anti-*H2AZ2*” gene signature to the Connectivity Map Analysis (CMA) (Fig. 5A and Supplementary Table S4). Briefly, CMA is a catalog of thousands of drug-induced gene expression profiles, which provides the opportunity to discover compounds that elicit similar (i.e., activators) or dissimilar (i.e., inhibitors) gene expression profiles to the query signature [38]. Using this approach, we identified palbociclib as a top hit confirming our previous finding that the E2Fs regulate *H2AZ2* expression (Supplementary Fig. S5A). Among our CMA hits, we also found TG101348 (a JAK2-selective inhibitor) and dovitinib (a multi-targeted RTK inhibitor), suggesting that inhibition of the RTK/JAK/STAT pathway may also downregulate *H2AZ2* (Supplementary Fig. S5A). We decided to focus on STAT3 inhibitors due to our previous observation that the *H2AZ2* gene promoter may also harbor STAT3 binding site (Supplementary Table S3**)**.

We first evaluated the effect of S3I-201 (a well-established STAT3 inhibitor) on GSC viability after a 3-day treatment. Treatment of GSCs with S3I-201 significantly decreased GSC viability in a dose-dependent manner (Fig. 5B). STAT3 inhibition led to a significant decrease in H2AZ mRNA and protein levels in GSC (Fig. 5C–E), which corresponded to reduced STAT3 occupancy on the enhancer region within the *H2AZ2* gene promoter (Fig. 5F, G). We also assessed the transcriptomic changes upon S3I-201 treatment of GSC. Reassuringly, S3I-201 treatment reduced the expression of a subset of genes in the “anti-*H2AZ2*” gene signature that is expected to be downregulated in our CMA (Supplementary Fig. S5B). In contrast to the downregulation of cell cycle pathway upon E2Fi treatment, S3I-201 treatment significantly downregulated genes that were enriched in pathways, including extracellular matrix organization (related to cell migration) in GSC (Figs. 4B and 5H. Taken together, these results indicate that STAT3 also regulates *H2AZ2* gene transcription in GSC.

### S3I-201 synergizes with E2Fi in reducing *H2AZ* expression and GSC viability

**Fig. 6:**
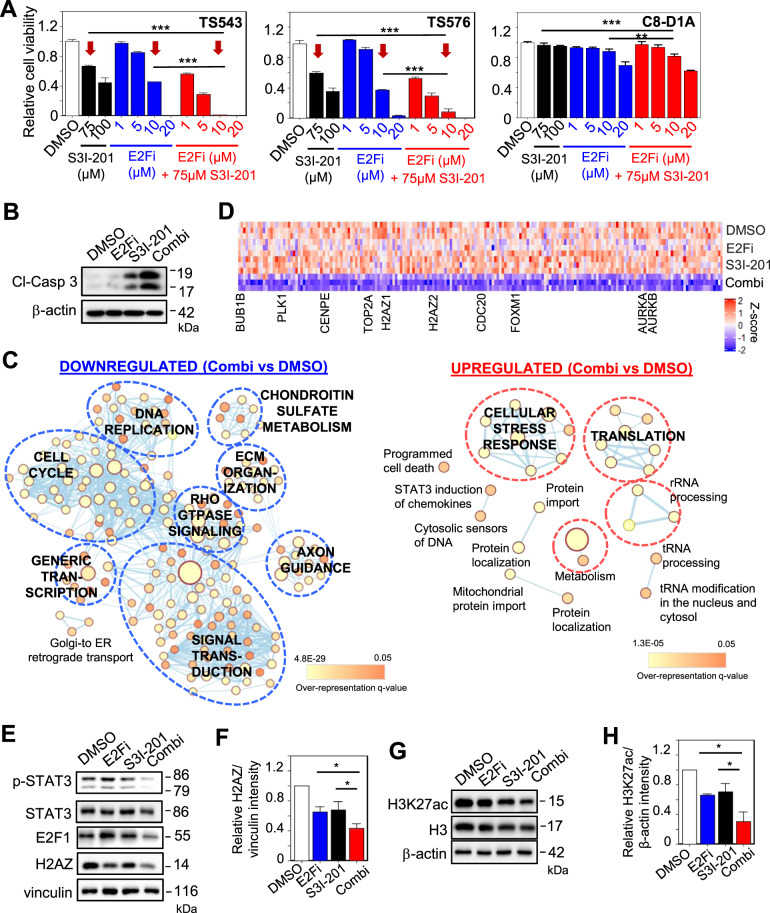
S3I-201 synergizes with E2Fi in reducing *H2AZ* expression and GSC viability. **A** Cell viability assay of GSCs and mouse astrocytes with 3 days treatment of S3I-201, E2Fi, or E2Fi/S3I-201 combination at the indicated concentrations (*n* = 8) (mean ± SD). ***p < 10e−5. **B** Western blot analysis of cleaved-caspase 3 protein levels with the respective drug treatment of GSC. β-actin serves the loading control. **C** Gene set enrichment map of pathways containing genes downregulated or upregulated upon 75 μM S3I-201/10 μM E2Fi combination treatment of GSC (3 days). Nodes represent gene sets (pathways) that were significantly enriched in the comparison treated vs. control samples (FDR < 0.01). **D** Heat map of cell cycle gene expression with DMSO, 10 μM E2Fi, 75 μM S3I-201, and 10 μM E2Fi + 75 μM S3I-201 (3 days) treatment of GSC. **E** Western blot analysis of p-STAT3, STAT3, E2F1, and H2AZ protein levels with the respective drug treatment of GSC. Vinculin serves the loading control. **F** Quantification of H2AZ band intensities in (**E**) when normalized to the vinculin control (*n* = 3) (mean ± SD). *p < 0.05. **G** Western blot analysis of H3K27ac and H3 protein levels with the respective drug treatment of GSC. β-actin serves the loading control. **H** Quantification of H3K27ac band intensities in (**G**) when normalized to the β-actin control (*n* = 3) (mean ± SD). *p < 0.05. **A**, **F**, **H** Two-tailed unpaired Student’s *t* test.

To address if E2F1 and STAT3 may regulate *H2AZ2* transcription in a synergistic or redundant manner, we first quantified GSC viability with E2Fi, S3I-201 and E2Fi/S3I-201 combination treatments (3 days). Strikingly, the combination of 75 μM S3I-201 and 10 μM E2Fi resulted in a reduction of cell viability that was significantly greater than that achieved with 75 μM S3I-201 or 10 μM E2Fi treatment alone (combination: >95%; S3I-201 or E2Fi: 40–60%) (Fig. 6A). This was accompanied by more cellular apoptosis in drug combination-treated GSC than that of single agent treatment (Fig. 6B). In contrast, there was only a modest decrease in the cell viability of non-cancerous mouse astrocytes under similar treatment conditions (~10%) (Fig. 6A). The synergistic anti-GSC activity from the E2Fi/S3I-201 combination was not due to off-target effects of E2Fi as similar results were obtained with the combination of palbociclib and S3I-201 (Supplementary Fig. S5C). RNA-Seq followed by ConsensusPathDB analysis of the DEGs revealed that the drug combination downregulated many pathways, including cell cycle, DNA replication, generic transcription, Rho GTPase signaling, signal transduction and extracellular matrix organization (Fig. 6C). We also observed that the drug combination led to the strongest decrease in cell cycle gene expression when compared to E2Fi or S3I-201 alone (Fig. 6D), which tracked with the greatest reduction of H2AZ and H3K27ac protein levels in GSC (Fig. 6E–H). Since E2F1 and STAT3 regulate *H2AZ2* gene transcription by binding to the enhancer region within *H2AZ2* gene promoter, we next asked if E2F1 can interact with STAT3. Using 293T cell lysates that have STAT3 and HA-tagged E2F1 being overexpressed, we found a faint E2F1 band in the STAT3 immunoprecipitates (Supplementary Fig. S5D). In contrast, no E2F1 was detected in the STAT3 immunoprecipitates from GSC lysates (Supplementary Fig. S5E). These STAT3 immunoprecipitation experiments suggest that E2F1 may weakly/ transiently interact with STAT3 as the E2F1-STAT3 interaction can only be detected when the levels of both proteins are high. Thus, we conclude that co-inhibiting E2F and STAT3 synergistically decreases H2AZ levels, chromatin accessibility (reflected by H3K27ac levels), cell cycle gene transcription and GSC viability.

### S3I-201/E2Fi combination potently and durably impairs GSC tumorigenicity

**Fig. 7:**
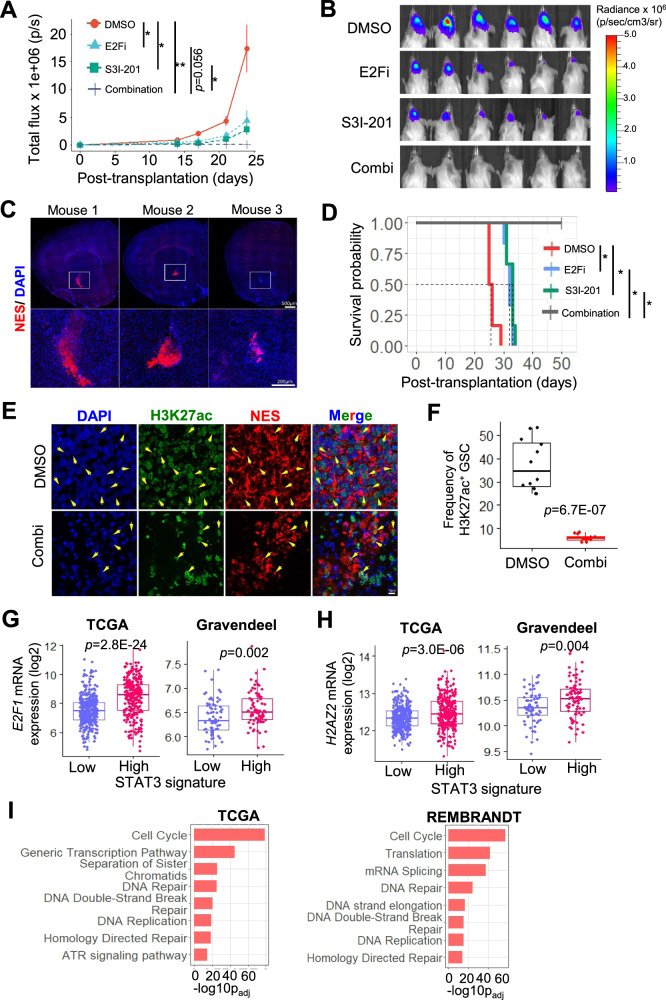
S3I-201/E2Fi combination potently and durably impairs GSC tumorigenicity. Quantification of tumor volume based on in vivo bioluminescence-based imaging (**A**) and representative images (**B**) of the tumor-bearing mice at 24 days posttransplantation (*n* = 6) (mean ± SD). The mice were challenged with of DMSO, 10 μM E2Fi, 75 μM S3I-201, or 10 μM E2Fi + 75 μM S3I-201-treated GSC TS543 (1 × 10^5^ cells; 3 days treatment). Two-tailed unpaired Student’s *t* test; *p < 0.05, **p < 0.01. **C** Representative images of mouse brains showing engraftment of drug combination-treated GSC as detected using a human-specific anti-NES antibody (*n* = 3). **D** Survival curves of mice from **A**. Log-rank test. *p < 0.001. Representative images (**E**) and frequency (**F**) of H3K27ac^+^ GSC (NES^+^) in DMSO vs. drug combination-treated GSC (*n* = 3, 4 sections per mouse brain) (mean ± SD). Comparison of *E2F1* (**G**) and *H2AZ2* (**H**) mRNA levels in TCGA and Gravendeel glioma patients based on a STAT3^high^ vs. STAT3^low^ gene signature. **I** Pathways that are overrepresented in *H2AZ2* correlated genes in glioma patients from TCGA and REMBRANDT cohorts using ConsensusPathDB analysis. **G**, **H** Mann–Whitney test, (**I**) hypergeometric test.

Although some CDK4/6 inhibitors can penetrate the blood-brain barrier, there are currently no such STAT3 inhibitors [39–42]. Thus, we generated orthotopic xenografts using GSCs that were pretreated with S3I-201, E2Fi or E2Fi/S3I-201 combination (3 days) to evaluate the durability of the respective drug treatment on tumor growth. Strikingly, GSCs with the drug combination treatment remained cytostatic while those with single agent treatment grew albeit at a slower rate when compared to vehicle control (Fig. 7A, B). We confirmed the engraftment of the drug combination-treated GSC in the mouse brains (50 days posttransplantation), as detected using a human NES-specific antibody, indicating that the cells were alive at the time of transplantation (Fig. 7C). Accordingly, all mice that were challenged with drug combination-treated GSC remained alive for more than 50 days posttransplantation, while those that bore GSCs with single agent treatment only had an extended survival of 7-8 days when compared to the vehicle control (Fig. 7D) (NB: *H2AZ2* KD extended median survival by 13 days (Fig. 2I)). These results corroborate with significantly less H3K27ac^+^ GSC in drug combination-treated GSC (at 50 days posttransplantation) when compared to the DMSO control (at mouse euthanasia) (Fig. 7E, F). Finally, we asked if the E2F/STAT3 inhibitor combination may find its utility in glioma treatment. In both TCGA and Gravendeel cohorts, gliomas with a STAT3^high^ gene signature (associated with inferior glioma patient survival and GBM [39]) significantly expressed higher *E2F1* and *H2AZ2* levels than tumors with a STAT3^low^ gene signature (Fig. 7G, H). To strengthen the idea that H2AZ2 also regulates cell cycle genes in glioma patients, we subjected genes that positively correlated with *H2AZ2* expression in gliomas to the ConsensusPathDB analysis, revealing an enrichment of the cell cycle pathway in both TCGA and REMBRANDT cohorts (Fig. 7I). Collectively, these findings demonstrate the superior anti-GBM activity of the E2F/STAT3 inhibitor combination and its potential application in GBM treatment.

## Discussion

In this study, we first ascertain the clinical and functional relevance of *H2AZ2* in GBM malignant progression before setting out to investigate its transcriptional regulation. On the basis of our ChIP-Seq, ATAC-Seq and RNA-Seq analyses, we propose the following model to explain how the H2AZ2 protein can regulate the transcription of *H2AZ2* and other GSC-critical genes. When the H2AZ2 protein is expressed, its deposition at enhancer region within gene promoters confers an “open” chromatin conformation that allows the access of E2F1 and STAT3 to the promoters of *H2AZ2* and other E2F and STAT3 target genes (Supplementary Fig. S6A). In this way, the H2AZ2 protein facilitates the transcription of these genes to promote GSC proliferation, self-renewal, invasiveness and tumorigenicity. Upon *H2AZ2* KD, a more compact chromatin conformation reduces the accessibility of E2F1 and STAT3 to the enhancer region within the promoters of E2F and STAT3 target genes. This decreases transcription of genes in the cell cycle (E2F-regulated) and Rho GTPase (STAT3-regulated) pathways, thereby impairing GSC function (Supplementary Fig. S6B). Co-inhibition of E2F/STAT3 decreases the transcription of *H2AZ2* and other E2F and STAT3 target genes (Supplementary Fig. S6C). The downregulation of *H2AZ2* gene transcription leads to less H2AZ2 protein, which in turn reduces H3K27ac levels and hence cell cycle gene expression (Fig. 6). Such epigenetic changes are a secondary effect of *H2AZ2* downregulation upon E2F/STAT3 co-inhibition. Thus, the E2F/STAT3 inhibitor treatment mimics *H2AZ2* KD (Figs. 4C and 6C). That E2F/STAT3 provide transcriptional synergy for *H2AZ2* gene activation is a phenomenon that has also been described in embryonic stem cells where the binding of multiple TFs to enhancer regions generate “enhanceosomes” that are proposed to mediate embryonic stem cell-specific gene expression [43]. Interestingly, the same study reported that STAT3 and E2F1 do not frequently co-occupy on these multiple TF-binding loci in embryonic stem cells. Thus, GBM has uniquely opted the use of E2F1- and STAT3-containing enhanceosomes that integrate multiple signaling pathways to achieve *H2AZ* gene activation. Since H2AZ levels are higher in GSCs than non-GSCs, this suggests that the GSCs are dependent on H2AZ-associated chromatin conformation to sustain their cell identity.

Previously, it was demonstrated that H2AZ2 recruits E2F1 and BRD2 to the promoters of cell cycle genes to drive melanoma proliferation and invasiveness [14]. From our transcriptomic comparison of *H2AZ2* KD and drug-treated GSC, we found that the downregulated pathways of *H2AZ2* KD cells resembled that of E2Fi/S3I-201 combination treatments, suggesting that both E2F1 and STAT3 chromatin occupancy may be compromised in the absence of H2AZ2. This would be consistent with our integrative analyses showing E2F1 and/or STAT3 target genes among the 99 high confidence H2AZ2-associated genes with reduced transcriptional output upon *H2AZ2* KD (Supplementary Fig. S4E, F). Notably, our mechanistic studies also illuminate a previously unrecognized E2F1-H2AZ2 feedforward loop wherein E2F1 transcriptionally activates *H2AZ2*, and the chromatin-incorporated H2AZ2 protein in turn facilitates the access of E2F1 to the promoters of E2F target genes. Thus, the E2F1-H2AZ2 feedforward loop may serve to ensure the uninterrupted transcription of cell cycle genes that is required for rapid GBM proliferation. Accordingly, *H2AZ2* overexpression can partially rescue the loss of GSC colony formation upon *E2F1* depletion, supporting the view that E2F1 functions in part through H2AZ2 to promote GSC proliferation (Supplementary Fig. S3F–H). Given the widespread H2AZ and E2F dysregulations across diverse cancer types, these findings suggest that the E2F-H2AZ feedforward loop may be a common theme in cancer.

As the Rb/E2F pathway is frequently deregulated in cancer, E2F inhibition using CDK4/6 inhibitors are widely explored as therapeutic agents in cancer. However, the dose-limiting toxicities of the CDK4/6 inhibitors and the inevitable resistance of tumor cells toward them have limited their clinical application, with the exception of ER+ metastatic breast cancer [44, 45]. As a result, there are now more than 100 clinical trials that attempt to combine CDK4/6 inhibitors with targeted therapies, chemotherapies or immunotherapy for development of more superior anti-cancer treatment options [44]. Our transcriptional studies of *H2AZ2* led us to explore the use of E2F/STAT3 inhibitor combination as an anti-GSC treatment. There are attempts to combine CDK4/6 inhibitors with MEK inhibitors (for RAS-driven cancers) or PI3K inhibitors (for breast cancer), but to our knowledge this study is the first to propose the use of E2F/STAT3 inhibitor combination for GBM treatment [46]. Strikingly, a 3-day treatment of GSC with the drug combination induced a sustained cytostatic effect (for as long as 50 days post-treatment) that is not achievable with single agent treatment, suggesting that H2AZ reduction beyond a certain threshold level may temporarily erase the “transcriptional memory” of GSC for cell cycle gene activation. “Transcriptional memory” is a phenomenon that occurs in genes that are frequently primed for reactivation and it allows cells to mount a more rapid transcriptional response to a previously experienced environmental challenge [47]. An example of “transcriptional memory” may be illustrated by the regulation of *INO1* expression in yeast. *H2AZ* deletion leads to the inability of yeast cells to retain *INO1* at the nuclear periphery after repression, failure to recruit RNA Pol II and thus a defect in the reactivation of *INO1* gene [47]. This corroborates with the established role of H2AZ in RNA Pol II recruitment and elongation, as well as maintenance of chromatin that can be readily remodeled for active transcription [48, 49].

In summary, we demonstrate that E2F1 and STAT3 provide transcriptional synergy for H2AZ activation that is required to sustain GBM chromatin accessibility and tumorigenicity. Our transcriptional studies of *H2AZ* also unveiled an E2F/STAT3 inhibitor combination that durably suppresses GSC tumorigenicity. The GSCs contribute to GBM proliferation, invasiveness, angiogenesis, plasticity and therapy resistance, thus represent the GBM subset that is the most attractive to target for effective new GBM treatment options [50]. Because numerous CDK4/6 and STAT3 inhibitors currently exist, including some that are already FDA-approved, it should be relatively straightforward to assess their clinical efficacy as a combinatorial therapy in GBM patients. With the challenge of delivering clinically useful doses of STAT3 inhibitors into the brain, our findings will also motivate efforts to develop new generation, blood-brain barrier-permeable STAT3 inhibitors that can subsequently be combined with CDK4/6 inhibitors for effective GBM eradication.

## Materials and methods

### Cell lines and compounds

Human GBM-derived GSCs were provided by Dr. Cameron Brennan (Memorial Sloan Kettering Cancer Center) and Dr. Ronald A. DePinho (MD Anderson Cancer Center). The GSCs were cultured in human neural stem cell Maintenance Media (Millipore), 1% Penicillin-Streptomycin (PS), and supplemented with EGF and bFGF (20 ng ml^−1^ each). Non-cancerous mouse astrocytes (C8-D1A from ATCC) were provided by Dr. Thiruma V. Arumugam (La Trobe University) and cultured with DMEM/F12 with 10% FBS and 1% PS. HEK293T cells were cultured with DMEM with 10% FBS and 1% PS. The following compounds were used in this study: Carboplatin (Tocris, 2626), S3I-201 (Selleckchem, S1155), HLM006474 (Selleckchem, S8963), Palbociclib (MedChemExpress, HY-50767).

### RNAscope assay

Fluorogenic RNAscope was performed on fixed cells or TMA (US Biomax) sections using company protocols. Briefly, TMA sections were baked at 60 °C for overnight, dewaxed and air-dried before pre-treatments. For all tissue sections a standard pre-treatment protocol was used. Test probes included Hs-OLIG2 (424191 Accession # NM_005806.3 – target region 959-2502), H2AZ2 (3000031 Accession #NM_138635.3 sequence region 1668-3217) and ITGA6 (559021 Accession # NM_001079818.1 -target region 831-1791) were used to stain the TMAs. Detection of specific probe binding sites was with RNAscope Multiplex Flourescent Reagent Kit v2 from ACD (Cat. No. 323100) with TSA Plus Cyanine3, and TSA Plus Cyanine5 (PerkinElmer). For semi-quantitative microscopical evaluations of control or test probe mRNA detection by RNAscope, the RNAscope®Assay Semi-Quantative Scoring Guideline was implemented. Based on manufacturer’s recommendation, score 0 corresponded to no staining or <1 dot/10 cells; score 1: 1–3 dots/cell; score 2: 4–9 dots/cell and/or no/very few dot clusters; score 3: 10–15 dots/cell and/or <10% dots are in clusters.

### DNA constructs

The shRNAs against human H2AZ2 (shH2AZ2#1, TRCN0000278497 and shH2AZ2#2, TRCN0000278496) and human E2F1 (shE2F1, TRCN0000010328) were purchased from Sigma. GSCs were transduced with viral particles. pDONR223_STAT3_WT (Plasmid #82235) and pCMVHA E2F1 (Plasmid #24225) were purchased from Addgene. The STAT3 ORF was subsequently shuttled into the pHAGE-EF1a-IRES-GFP vector (kindly provided by Dr. Ronald A. DePinho from MD Anderson Cancer Center) using Gateway cloning.

### In vitro limiting dilution and tumorsphere formation assays

GSCs were stained with PI, and PI-negative cells (*n* > 6) were flow-sorted with decreasing number of cells per well (1, 10, 25, and 100) plated in 96-well plates. The percentage of wells with tumorspheres was quantified after 7 days under a microscope. Extreme limiting dilution analysis was performed using software available at http://bioinf.wehi.edu.au/software/elda/. The tumorsphere formation assay involved seeding GCs at a density of 1 cell per µl, and the number of tumorspheres in each well was quantified after 7 days.

### Anchorage-independent growth assay

Anchorage-independent growth assays were performed in replicates of four in six-well plates. Indicated cells were seeded (1 × 10^4^ cells per well) in stem cell proliferation media with EGF and βFGF containing 0.5% low-melting agarose on the top of bottom agar containing 1% low-melting agarose stem cell proliferation media with EGF and βFGF. After 14–21 days, colonies were stained with iodonitrotetrazoliumchloride (Sigma) and counted. Each assay was performed in triplicate.

### Transwell migration and invasion assay

The invasiveness of GSCs was measured using 6.5 mm Transwell with 8.0 µm pore polycarbonate membrane insert (Corning, CLS3422). Membrane was coated with Matrigel Basement Membrane Matrix (100 µg/cm^2^) (BD Biosciences). The drug-treated cells were seeded in the upper compartment with serum-free GSC medium. The wells of the lower chamber were filled with GSC medium containing 10% FBS. At the end of the invasion assay, chambers were removed, fixed, and stained with a 0.5% Crystal Violet. Cells on the upper surface of the filters were removed by wiping with a cotton swab, and invasion was determined by counting the cells that migrated to the bottom side of the filter using at least ten fields per insert at ×20 magnification. Each assay was performed in triplicate.

### Immunofluorescence

For immunofluorescence, GSCs, and mouse brain samples were fixed, blocked, and incubated with anti-BrdU (Biolegend, 339802), Nestin (Merck Millipore, MAB5326), or H3K27ac (Abcam, ab4729) for overnight at 4 °C. Following, secondary antibodies conjugated with Alexa 488 (Molecular Probes) or Alexa 555 (Molecular Probes) were applied. Images were captured with a Leica DCF 9000 GT digital camera, using a Leica DMi8 microscope. Data presented are from two independent experiments with similar results.

### RNA isolation and qRT-PCR

RNA was isolated with RNeasy® Mini or Micro Kit (Qiagen), and then used for first-strand cDNA synthesis using random primers and SuperScriptIII Reverse Transcriptase (Invitrogen). qRT-PCR was performed using PowerUp™ SYBR® Green Master Mix (Applied Biosystems). Primers are listed in Table S5. The relative expression of genes was normalized using the indicated housekeeping genes. Each assay was performed in triplicate.

### Western blot analysis and antibodies

Whole cell lysates were prepared in RIPA buffer (Thermo) with protease inhibitor (Roche), and phosphatase inhibitor (Roche). For histone protein, cells were incubated with 0.5 Triton X-100 for 10 min, and pellets were incubated with 0.2 N HCl overnight at 4 °C. Protein concentration was determined by DC Protein Assay (Bio-rad), and equal amount of protein samples was used to perform SDS gel electrophoresis and transferred onto nylon membranes (Bio-rad). TBST with 5% skim milk was used for blocking. Incubation with primary antibody was performed at 4 °C for 16 h. The following antibodies were used: Cleaved-caspase3 (Cell Signaling, 9661), H3K27ac (Abcam, ab4729), H3K4me3 (Abcam, ab8580), H3K9me3 (Abcam, ab8898), H3 (Abcam, ab1791), phospho-STAT3 (Cell Signaling, 9145), STAT3 (ZooMAB, ZRB-1004 and Cell Signaling, 9139), E2F1 (Millipore, 05-376), H2AZ (Active Motif, 39113), HA (Proteintech, 51064-2-AP), Vinculin (Sigma, V9131) and β-actin (Sigma, A5316), HRP-linked anti-rabbit IgG antibody (Cell Signaling, 7074), HRP-linked anti-mouse IgG antibody (Cell Signaling, 7076). Quantification of protein expression was performed using Image J, and either Vinculin or β-actin was used as the loading control for protein normalization. Each assay was performed in triplicate.

### RNA-seq analysis

GSC TS543 were transduced with non-targeting and H2AZ2 shRNAs, or treated with DMSO, 75 µM S3I-201, 10 µM HLM006474, 15 µM HLM006474, 75 µM S3I-201, or 10 µM HLM006474 + 75 µM S3I-201 for 3 days (three biological replicates per condition). Total mRNA samples were sent to Axil Scientific Pte Ltd, Singapore and NovogeneAIT Genomics Singapore for RNA-Seq analysis. Transcriptomic sequencing (RNA-Seq) was performed on the Illumina HiSeq platform according to the standard paired-end protocol. RNA-seq data quality was monitored via FASTQC package (https://www.bioinformatics.babraham.ac.uk/projects/fastqc/). Adapters and overrepresented sequences have been removed using cutadapt software (https://cutadapt.readthedocs.io/en/stable/). Further reads preprocessing was performed by Trimmomatic (version 0.36) with the parameters: LEADING:3 TRAILING:3 SLIDINGWINDOW:4:15 MINLEN:50. Mapping of RNA-seq reads was done using STAR_2.5.0a [51] with default parameters for RNA-seq data; RSEM software [52] were used to quantify the gene-level expression. EBSeq package was utilized for differential gene expression analysis. Gene set/pathway enrichment analysis was performed using the ConsensusPathDB (http://consensuspathdb.org/). Functional pathway enrichment visualization was performed via Enrichment Map plugin in Cytoscape (version3.7.1) (https://cytoscape.org/). In order to generate the Enrichment Map plots, the lists of significantly enriched terms (*q* value < 0.05) under REACTOME were used as an input for the Enrichment Map plugin.

Figure S5B was generated using the filtered gene list of “Anti-H2AZ2” gene signature used as query in Connectivity Map Analysis. Filtering was performed by selecting all significantly DEGs between two conditions (*p*adj < 0.05, DMSO vs. treatment with S3I-201) in the list with fold changes either >1.3 or <−1.3 for both up- and downregulated genes.

### ChIP-seq and ChIP-qPCR analyses

Briefly, cells were cross-linked with 1% formaldehyde for 10 min at room temperature. The cells were lysed using SDS Lysis buffer for ChIP (1% SDS, 10 mM EDTA, 50 mM Tris-HCl pH 8). The lysate was then sonicated for 25 cycles at 30% amplitude (15 s ON and 45 s OFF). The sonicated samples were then diluted in ChIP dilution buffer (0.01% SDS, 1% Triton X-100, 1.2 mM EDTA, 16.7 mM Tris-HCl pH 8, 167 mM NaCl) and used for the immunoprecipitation with anti-H3K4me3 (Abcam, Ab8580) or H3K27ac (Abcam, Ab4729) for ChIP-Seq, and rabbit IgG, mouse IgG, STAT3 (Cell Signaling, 9139), E2F1 (Millipore, 05-376), or H3K27ac for ChIP-qPCR with protein A/G agarose beads. After an overnight incubation with antibody, the bound DNA was washed sequentially with low salt wash buffer (0.1% SDS, 1% Triton X-100, 2 mM EDTA, 20 mM Tris-HCl pH 8, 150 mM NaCl), high salt wash buffer (0.1% SDS, 1% Triton X-100, 2 mM EDTA, 20 mM Tris-HCl pH 8, 500 mM NaCl), LiCl wash buffer (0.25 M LiCl, 1% NP40, 1%deoxycholate, 1 mM EDTA, 10 mM Tris-HCl pH 8) and TE wash buffer (10 mM Tris-HCl pH 8, 1 mM EDTA) to remove non-specific sequences and eluted in the elution buffer (84 mg NaHCO3, 1 ml 10% SDS, 9 ml H_2_O). Then the samples were reverse cross-linked using NaCl at 65 °C overnight. The eluted DNA was purified and used for library preparation or ChIP-qPCR.

Library preparation was performed as follows. Briefly, end repair with NEBNext End Repair enzyme (NEB, E6050) and clean-up with 2.4x volume AMPure XP beads (Beckman Coulter, A63880). A-tailing with Klenow Fragment (3′ → 5′ exo-, NEB, M0212) and purified with 2.4x volume AMPure XP beads. Adapter ligation reactions contained annealed universal adapter and T4 rapid ligase (NEB, B0202) and clean-up with 1.8x volume AMPure XP beads. Chromatin was amplified using KAPA Real-time Library Amplification Kit (KAPABIOSYSTEMS, KK2701) with universal primer and barcoded primer. Amplified chromatin was purified with QIAquick Gel Extraction Kit (Qiagen). Processing of ChIP-Seq data was very similar to the analysis of ATAC-Seq data, except that the significant ChIP-Seq peaks were identified using the MACS2 software (version 2.1.1.20160309) [53] at a cutoff of *q* value 0.05.

In total, 12,306 putative gene targets of H2AZ in Fig. 3D have been obtained by annotating H2AZ ChIP-Seq regions generated in the current study using HOMER software. Only the genes with TSS located ≤1 kb and ≥−1 kb from nearest H2AZ ChIP-Seq peak border have been selected for further analyses.

ChIP-qPCR was performed using PowerUp™ SYBR® Green Master Mix (Applied Biosystems). Primers are listed in Table S5. Data presented are from two independent experiments with similar results.

### ATAC-seq analysis

Cells were harvested and frozen in culture media containing 5% DMSO. Frozen cells were sent to Active Motif to perform the ATAC-seq assay. The cells were then thawed in a 37 °C water bath, pelleted, washed with cold PBS, and tagmented as previously described [54]. Briefly, cell pellets were resuspended in lysis buffer, pelleted, and tagmented using the enzyme and buffer provided in the Nextera Library Prep Kit (Illumina). Tagmented DNA was then purified using the MinElute PCR purification kit (Qiagen), amplified with ten cycles of PCR, and purified. Resulting material was quantified using the KAPA Library Quantification Kit for Illumina platforms (KAPA Biosystems), and sequenced with PE42 sequencing on the NextSeq 500 sequencer (Illumina).

Reads were filtered based on quality and adapter sequences were removed from the ATAC-Seq experiments using Trim_galore (https://github.com/FelixKrueger/TrimGalore) with the default options. The resulting fastq files were aligned to the human reference genome (hg19) using *STAR*_2.5.0a [51] with the following parameters: “-alignIntronMax 1”, “-outFilterMismatchNoverLmax 0.09”, “-alignMatesGapMax 2000”, “-outFilterMultimapNmax 1”, “-alignEndsType EndToEnd“; the rest of the options were set to the default. Duplicated reads were removed from the bam files using *MarkDuplicates* (http://broadinstitute.github.io/picard/) and subsequently transformed to Bed format using *bamToBed* from *Bedtools* [55]. Differential open chromatin regions were called with *DiffReps* [56] using a negative binomial test and the following parameters “-window 100”, “-gname hg19” and “-frag 0”; the rest of the options were set to default. The resulting set of genomic locations were considered to have a significant change in ATAC-Seq signal only if: the regions had a minimum coverage of 50 reads in average for at least one of the conditions, a *p* adjusted value lower than 0.05, a log_2_ fold change of at least 0.5 in either direction and if they did not overlapp with the ENCODE blacklisted regions.

Bam files were converted to bigWig for visualization purposes using *deepTools* [57], normalizing by library size and transforming the values to “counts per million”. *ComputeMatrix* and *plotHeatmap* from *deepTools* were used to quantify and plot the heatmaps of the ATAC-Seq signal surrounding the differentially open chromatin regions. We used the results of the *hyper geometric test* from the biological functions GO table, using the hg19 assembly and the default settings of the *Basal plus extension* mode. The differentially open chromatin regions were annotated with annotatePeaks from the HOMER software [58] and the distribution was plotted with R. Using the HOMER annotation, the regions with differential ATAC-seq signal were divided into two groups: promoter regions (overlapping with the ±1 Kb region around the TSS) or distal from TSS. Annotating promoter-associated significantly decreased peaks upon *H2AZ2* KD allowed us identify 1336 putative genes functionally associated with H2AZ2. To produce Supplementary Table S1, 1336 genes functionally associated with H2AZ2 were intersected with 12,306 putative gene targets of H2AZ obtained by ChIP-Seq, resulting in 992 high confidence H2AZ2-associated gene targets. Only top 500 genes out of them are presented in the table.

ReMap [59] was used with default parameters to calculate TF enrichment in differentially open chromatin regions at gene promoters. To generate Table S2, the top selected TFs enriched within reduced chromatin accessibility regions at proximal promoters were also verified on positive correlation with worse clinical outcome in TCGA glioma patient cohort.

### Connectivity map analysis (CMA)

To identify candidate upstream regulators of H2AZ2 expression we used L1000CDS2 drug screening database [60] (https://maayanlab.cloud/L1000CDS2/#/index). Gene symbols positively and negatively correlated with H2AZ2 expression in TCGA glioma cohort (Pearson correlation coefficient >0.40 and <−0.40, respectively, Table S4) were used as “downregulated” and “upregulated” input genes in the L1000CDS2 search engine, respectively. The L1000CDS2 calculates the pair-wise cosine distance between the directions of the disease-drug characteristics and provides ranked lists of scores for the candidate compounds. First, the search engine prioritized small-molecules that were predicted to mimic expression pattern of the H2AZ2 correlation signature. Then, we calculated the aggregated score of each compound which took into account the compound gene pattern consistency in multiple cell lines: (1) average score value for original L1000CDS2 cell line scores for each compound hit was calculated; (2) average score value for each drug hit was multiplied by number of independent cell line hits to obtain the aggregated score for each compound.

### Cell viability assay

Cell-Titer Glow (Promega) was used to determine cell proliferation rate. Briefly, GSCs and mouse astrocytes were seeded on the 96 wells, following day cells were treated with different inhibitors. Cell viability assay was performed 3 days after with Cell-Titre Glow. Data was normalized to DMSO treatment. Each assay was performed in triplicate.

### Immunoprecipitation

GSC and HEK293T cells were lysed in IP lysis buffer (50 mM Tris-Cl at pH 7.5, 150 mM NaCl, 1% Triton X-100) supplemented with protease inhibitor and phosphatase inhibitor. Equal amount of proteins was incubated with the indicated antibody and Protein A/G agarose (Thermo Fisher, 20421) at 4 °C for 16 h. Beads were washed with IP lysis buffer five times and the bound proteins were eluted with 2x SDS sample loading dye. The same volume of eluted proteins was resolved using SDS electrophoresis and detected using western blot analysis.

### Intracranial tumor formation in vivo

GSCs (1 × 10^5^ viable cells) were grafted intracranially into NSG mice (InVivos) aged 6–8 weeks. Tumor incidence was determined at indicated timepoints by luciferase imaging of mice using Xenogen IVIS (PerkinElmer) according to manufacturer’s instructions. Animals were maintained until neurological signs were apparent, at which point they were sacrificed.

### Public datasets and data analyses

Processed tumor gene expression and clinical data for TCGA (RNA-seq; glioma and GBM datasets), REMBRANDT and Gravendeel glioma patients’ cohorts have been obtained from GlioVis portal (http://recur.bioinfo.cnio.es/). Histone gene expression profiling in four glioma stem cell lines in Fig. 1B was done using an in-house microarray dataset.

For association analysis of gene expression of *H2AZ1* and *H2AZ2* with hallmark GBM genetic phenotypes (*IDH1* gene mutation status, *MGMT* promoter methylation, *TP53* and *PTEN* mutation status, *PDGFRA* and *CDK4*/*CDK6* amplification) TCGA patient genetic data were downloaded either from GlioVis portal or from UCSC Xena browser (https://xenabrowser.net/).

The single cell gene expression data for GBM was downloaded from NCBI GEO database (GSE57872) and analyzed as follows: (1) processed single cell data have been ranged based on the stem cell marker gene expression (FABP7); (2) H2AZ2 and H2AZ1 gene expression values were compared between the cells from the 1st (0–25%, “low marker expression”) and the last (75–100%, “high marker expression”) gene expression quartiles, respectively. Bulk RNA-Seq gene expression data to compare H2AZ2 expression between GSC and GBM tumors was obtained from Mack et al. [61].

E2F1 and STAT3 ChIP-Seq occupancy regions and ChIP-PCR primers in proximity of H2AZ2 promoter have been determined by analyzing public ChIP-Seq data for multiple cancer cell lines (HeLa, LNCap, MDA-MD231, U87, MCF10A, HCC70, SU-DHL-2) downloaded from ReMap database [59].

Gene list of 3165 direct STAT3 targets with ChIP-Seq binding sites within the 1 kb window near TSS in U87 GBM cells have been obtained from Zhang et al. [62]. 11730 direct E2F1 gene targets in U87 GBM cells with the same characteristics (1 kb window near TSS) have been retrieved as follows. Processed E2F1 binding regions narrowPeak file was downloaded from GEO: GSE99171; ChIP-Seq regions were annotated using HOMER software [59]. Only the genes with TSS located ≤500 bp and ≥−500 bp from nearest E2F1 ChIP-Seq peak have been selected for further analyses.

STAT3 functionally-tuned gene signature derived from Tan et al. [39] was utilized to stratify glioma patients into STAT3^low^ and STAT3^high^ subgroups from TCGA and Gravendeel glioma patients’ cohorts. Patients stratification was performed using Nearest Template Prediction method using GenePattern portal (https://www.genepattern.org/use-genepattern). Only stratified glioma patients samples with statistical significance (Benjamini-Hochberg FDR *p* value < 0.05, 1000 permutation tests) were included into the analysis.

In order to estimate whether the expression of gene of interest was significantly associated with cancer patient’s survival, we used the one‐dimensional data‐driven grouping (1‐D DDg) method [63]. Briefly, after sorting the patients’ data by the gene expression values, the values were fitted to survival times and events using the Cox proportional hazards model; goodness-of-fit analysis was applied to get the separation between the sorted patients into low- and high-risk subgroups. To compute the differences between the Kaplan–Meier survival curves, we used the Cox hazards model and Wald test statistic. R package survminer was used for visualization of survival curves.

### Statistical analyses

Two-tailed Student’s *t* test and Man–Whitney test were performed using either R3.4.1 or Cytel Studio (Version 9.0.0). Significance was defined as *p* < 0.05. For multiple testing correction Benjamini-Hochberg statistic was applied to estimate the FDR. 1‐D DDg method was used to estimate the significance of association of H2AZ2 gene with cancer patient’s survival. SigmaPlot (Version 11.0) or a set of R packages (ggplot2, ggpubr ComplexHeatmap and survminer) were implemented for plots generation. For all experiments with error bars, standard error mean was calculated to indicate the variation within each experiment and data, and values represent mean ± SD, as indicated in the figure legends.

## Supplementary information

Supplementary Table

Supplementary Figure Legend

Supplementary Figure 1

Supplementary Figure 2

Supplementary Figure 3

Supplementary Figure 4

Supplementary Figure 5

Supplementary Figure 6

## Data Availability

All data, supplementary data, and data in repositories are available. Raw and processed data from RNA-Seq of *H2AZ2* KD or drug-treated GSC TS543, ChIP-Seq data of H2AZ, H3K27ac, and H3K4me3 in GSC TS543, as well as ATAC-Seq data of *H2AZ2* KD GSC TS543 are available on the Gene Expression Omnibus database (GSE152861, GSE152862, GSE152858, GSE189781).
