# Supplementary material for: E2F and STAT3 provide transcriptional synergy for histone variant H2AZ activation to sustain glioblastoma chromatin accessibility and tumorigenicity
Source: Cell Death Differ. 2022 Jan 20;29(7):1379–94. doi: 10.1038/s41418-021-00926-5 (PMC9287453; doi:10.1038/s41418-021-00926-5)
Supplement: Supplementary file 1 — Supplementary Table [file 41418_2021_926_MOESM1_ESM.docx]

**SUPPLEMENTARY TABLES**

E2F and STAT3 provide transcriptional synergy for histone variant H2AZ activation to sustain glioblastoma chromatin accessibility and tumorigenicity

Jeehyun Yoon^1,2†^, Oleg V. Grinchuk^1,2†^, Roberto Tirado Magallanes^3†^, Zhen Kai Ngian^4^, Emmy Xue Yun Tay^1,2^, You Heng Chuah^1,2^, Bernice Woon Li Lee^1,2^, Jia Feng^1,2^, Karen Carmelina Crasta^1,2,5,6^, Chin Tong Ong^4,7^, Touati Benoukraf^3,8^, Derrick Sek Tong Ong^1,2,6,9^*

**Supplementary Table S1. Top 500 high confidence H2AZ2-associated genes with significantly decreased ATAC-Seq peaks (±1kb from TSS of neighbouring genes) that also harbor H2AZ ChIP-Seq binding sites (±1kb from TSS).**

| **Peak#** | **Chr** | **Start of ATAC-seq peak** | **End of ATAC-seq peak** | **Nearest PromoterID** | **Gene symbol** | **Distance to TSS** | **log2FC** | **padj** |
| --- | --- | --- | --- | --- | --- | --- | --- | --- |
| 1 | chr6 | 119031212 | 119031580 | NM_001178035 | CEP85L | -158 | -1.59 | 6.69E-08 |
| 2 | chr2 | 20850862 | 20851110 | NM_022460 | HS1BP3 | -122 | -0.65 | 9.88E-08 |
| 3 | chr15 | 40074942 | 40075150 | NM_001324338 | FSIP1 | -7 | -0.64 | 9.88E-08 |
| 4 | chr19 | 50354082 | 50354400 | NM_001305105 | PTOV1 | -136 | -0.77 | 0.000000109 |
| 5 | chr4 | 17513652 | 17513980 | NM_001306140 | QDPR | 41 | -0.68 | 0.000000114 |
| 6 | chr16 | 20817742 | 20817920 | NM_001142725 | ERI2 | -36 | -0.56 | 0.000000114 |
| 7 | chr9 | 4741062 | 4741500 | NM_001199852 | AK3 | 28 | -0.78 | 0.000000115 |
| 8 | chr16 | 81130112 | 81130360 | NR_033249 | GCSH | -256 | -0.58 | 0.000000124 |
| 9 | chr13 | 25496942 | 25497120 | NR_047594 | CENPJ | -4 | -0.51 | 0.000000132 |
| 10 | chr18 | 47013482 | 47013840 | NM_001199346 | C18orf32 | -17 | -0.73 | 0.000000142 |
| 11 | chr1 | 113615682 | 113615910 | NM_014813 | LRIG2 | 4 | -0.61 | 0.000000142 |
| 12 | chr10 | 86184502 | 86184910 | NM_001284243 | CCSER2 | 11 | -0.78 | 0.000000143 |
| 13 | chr2 | 64068722 | 64069010 | NM_006759 | UGP2 | -148 | -0.69 | 0.000000144 |
| 14 | chr6 | 20212642 | 20212930 | NM_001080480 | MBOAT1 | -91 | -0.69 | 0.000000147 |
| 15 | chr4 | 1714192 | 1714620 | NM_006527 | SLBP | 62 | -0.92 | 0.000000149 |
| 16 | chr5 | 140044252 | 140044560 | NM_017706 | WDR55 | 22 | -0.67 | 0.000000152 |
| 17 | chr10 | 103113582 | 103113920 | NM_001256856 | BTRC | -39 | -0.59 | 0.000000162 |
| 18 | chr15 | 45003592 | 45003850 | NM_004048 | B2M | 36 | -0.62 | 0.000000169 |
| 19 | chr17 | 73043032 | 73043410 | NM_015353 | KCTD2 | -58 | -0.56 | 0.000000174 |
| **20** | **chr7** | **44887792** | **44888130** | **NM_012412** | **H2AZ2** | **-236** | **-0.55** | **0.000000177** |
| 21 | chr10 | 101380532 | 101380830 | NM_031212 | SLC25A28 | -460 | -0.56 | 0.000000177 |
| 22 | chr2 | 238969372 | 238969590 | NM_016510 | SCLY | -84 | -0.52 | 0.000000184 |
| 23 | chr8 | 77593222 | 77593550 | NM_024721 | ZFHX4 | -129 | -0.98 | 0.00000019 |
| 24 | chr3 | 123303942 | 123304200 | NM_001329783 | HACD2 | -39 | -0.57 | 0.000000199 |
| 25 | chr15 | 91260282 | 91260570 | NR_120372 | CRTC3-AS1 | -55 | -0.66 | 0.0000002 |
| 26 | chr9 | 95432272 | 95432700 | NM_022755 | IPPK | 61 | -0.61 | 0.0000002 |
| 27 | chr10 | 102672142 | 102672520 | NM_018121 | SLF2 | 5 | -0.85 | 0.0000002 |
| 28 | chr14 | 50087282 | 50087570 | NM_001001 | RPL36AL | -23 | -0.65 | 0.000000211 |
| 29 | chr3 | 37034582 | 37034930 | NM_014805 | EPM2AIP1 | 39 | -0.51 | 0.000000224 |
| 30 | chr2 | 25264812 | 25265250 | NM_014971 | EFR3B | 58 | -0.68 | 0.00000023 |
| 31 | chr18 | 71815492 | 71815880 | NM_014177 | TIMM21 | -60 | -0.57 | 0.00000024 |
| 32 | chr19 | 37663542 | 37663860 | NM_152655 | ZNF585A | -58 | -0.65 | 0.000000241 |
| 33 | chr5 | 139943792 | 139944130 | NM_080670 | SLC35A4 | -189 | -0.88 | 0.000000262 |
| 34 | chr13 | 78315042 | 78315520 | NM_001242869 | SLAIN1 | -14 | -0.8 | 0.000000268 |
| 35 | chr13 | 95131852 | 95132440 | NM_001922 | DCT | -210 | -1.12 | 0.000000271 |
| 36 | chr16 | 67595882 | 67596190 | NM_006565 | CTCF | -274 | -0.6 | 0.000000273 |
| 37 | chr8 | 87520812 | 87521060 | NM_001286719 | RMDN1 | 73 | -0.76 | 0.000000275 |
| 38 | chr13 | 46964302 | 46964710 | NM_001286761 | RUBCNL | -329 | -0.57 | 0.000000294 |
| 39 | chr3 | 149376182 | 149376580 | NR_040250 | WWTR1-AS1 | 384 | -0.71 | 0.000000349 |
| 40 | chr1 | 179262702 | 179263060 | NM_001252511 | SOAT1 | 32 | -0.79 | 0.00000036 |
| 41 | chr13 | 30169802 | 30170220 | NM_003045 | SLC7A1 | -186 | -0.95 | 0.000000454 |
| 42 | chr2 | 179315382 | 179315640 | NM_001139517 | PRKRA | -27 | -0.94 | 0.000000518 |
| 43 | chr2 | 172290442 | 172290720 | NM_001321155 | METTL8 | 38 | -0.51 | 0.000000531 |
| 44 | chr22 | 20067342 | 20067670 | NM_001190326 | DGCR8 | -249 | -0.75 | 0.000000565 |
| 45 | chr12 | 120632542 | 120632850 | NM_006836 | GCN1 | -183 | -0.78 | 0.000000879 |
| 46 | chr13 | 58205302 | 58205560 | NM_001040429 | PCDH17 | -358 | -0.74 | 0.0000011 |
| 47 | chr1 | 179923592 | 179924040 | NM_014810 | CEP350 | -92 | -0.58 | 0.00000117 |
| 48 | chr10 | 1094772 | 1094960 | NM_001317957 | IDI1 | -43 | -0.67 | 0.00000163 |
| 49 | chr8 | 37887742 | 37888080 | NM_004095 | EIF4EBP1 | -109 | -0.82 | 0.00000211 |
| 50 | chr14 | 53417682 | 53417950 | NM_001135000 | FERMT2 | -1 | -0.61 | 0.00000264 |
| 51 | chr5 | 92957122 | 92957410 | NR_031754 | MIR2277 | -772 | -0.96 | 0.0000027 |
| 52 | chr4 | 76439472 | 76440020 | NM_144721 | THAP6 | 103 | -0.74 | 0.00000272 |
| 53 | chr19 | 58987352 | 58987760 | NM_017908 | ZNF446 | 25 | -0.63 | 0.00000317 |
| 54 | chr17 | 58603532 | 58603770 | NM_006380 | APPBP2 | -50 | -0.71 | 0.00000348 |
| 55 | chr4 | 103748912 | 103749280 | NM_003340 | UBE2D3 | 9 | -0.67 | 0.00000353 |
| 56 | chr1 | 228353722 | 228353920 | NM_001010867 | IBA57 | 312 | -0.61 | 0.0000037 |
| 57 | chr1 | 33641962 | 33642320 | NM_001330483 | TRIM62 | 10 | -0.9 | 0.00000425 |
| 58 | chr3 | 123339142 | 123339600 | NM_053032 | MYLK | 83 | -0.92 | 0.00000432 |
| 59 | chr20 | 44462132 | 44462390 | NM_001042632 | SNX21 | -209 | -0.72 | 0.00000452 |
| 60 | chr11 | 33278682 | 33278920 | NM_005734 | HIPK3 | -67 | -0.82 | 0.00000538 |
| 61 | chr2 | 228028202 | 228028630 | NM_000092 | COL4A4 | 859 | -1.54 | 0.00000544 |
| 62 | chr10 | 81107082 | 81107370 | NM_005729 | PPIF | 6 | -0.96 | 0.00000608 |
| 63 | chr11 | 111472832 | 111473150 | NM_015191 | SIK2 | -124 | -0.66 | 0.00000613 |
| 64 | chr2 | 9983322 | 9983650 | NM_005680 | TAF1B | -85 | -0.86 | 0.00000619 |
| 65 | chr13 | 48611462 | 48611810 | NM_018283 | NUDT15 | -67 | -0.9 | 0.00000653 |
| 66 | chr6 | 135818862 | 135819130 | NR_026805 | LINC00271 | 57 | -0.7 | 0.00000776 |
| 67 | chr5 | 56469682 | 56470040 | NM_001203246 | GPBP1 | 86 | -0.63 | 0.00000854 |
| 68 | chr2 | 54683332 | 54683540 | NM_003128 | SPTBN1 | -18 | -0.81 | 0.00000932 |
| 69 | chr19 | 36119572 | 36119910 | NM_024321 | RBM42 | -177 | -0.62 | 0.0000104 |
| 70 | chr18 | 52989462 | 52989680 | NM_001243234 | TCF4 | -481 | -0.86 | 0.0000127 |
| 71 | chr7 | 87505512 | 87505910 | NM_018843 | SLC25A40 | -19 | -0.57 | 0.0000128 |
| 72 | chr2 | 33359312 | 33359780 | NM_001166265 | LTBP1 | -118 | -0.77 | 0.000013 |
| 73 | chr1 | 85931542 | 85931870 | NM_012137 | DDAH1 | -817 | -1.16 | 0.0000135 |
| 74 | chr3 | 11313842 | 11314170 | NM_006395 | ATG7 | -4 | -0.7 | 0.0000139 |
| 75 | chr17 | 35716272 | 35716590 | NM_198836 | ACACA | -372 | -0.7 | 0.0000144 |
| 76 | chr5 | 146435462 | 146436020 | NM_181674 | PPP2R2B | -41 | -0.79 | 0.0000152 |
| 77 | chr9 | 34458482 | 34458820 | NM_001184940 | FAM219A | -83 | -0.63 | 0.0000158 |
| 78 | chr2 | 176032952 | 176033350 | NR_045773 | ATF2 | -217 | -0.53 | 0.0000169 |
| 79 | chr9 | 91933542 | 91933800 | NM_001282690 | SECISBP2 | -95 | -0.65 | 0.0000201 |
| 80 | chr6 | 126070192 | 126070600 | NM_012259 | HEY2 | -336 | -1 | 0.000024 |
| 81 | chr6 | 90062442 | 90062620 | NM_016021 | UBE2J1 | 88 | -0.7 | 0.000024 |
| 82 | chr18 | 11980982 | 11981270 | NM_014214 | IMPA2 | -301 | -0.53 | 0.0000243 |
| 83 | chr2 | 210288592 | 210288870 | NM_001039538 | MAP2 | -40 | -0.69 | 0.0000248 |
| 84 | chr9 | 88741872 | 88742130 | NR_110996 | LOC101927623 | -454 | -1.02 | 0.0000256 |
| 85 | chr12 | 69081132 | 69081420 | NM_020401 | NUP107 | 545 | -1.03 | 0.0000256 |
| 86 | chr7 | 18535102 | 18535360 | NM_001204146 | HDAC9 | -102 | -0.51 | 0.0000274 |
| 87 | chr3 | 121553802 | 121554070 | NM_001023571 | IQCB1 | -10 | -0.67 | 0.000028 |
| 88 | chr12 | 105478162 | 105478410 | NM_001034173 | ALDH1L2 | 55 | -0.81 | 0.0000295 |
| 89 | chr4 | 77135062 | 77135260 | NM_001242936 | FAM47E | -32 | -0.75 | 0.0000306 |
| 90 | chr16 | 90086972 | 90087460 | NM_001288708 | DBNDD1 | -677 | -0.69 | 0.0000319 |
| 91 | chr12 | 124018322 | 124018640 | NM_001319243 | RILPL1 | -216 | -0.85 | 0.0000329 |
| 92 | chr14 | 64805482 | 64805710 | NR_073496 | ESR2 | -328 | -0.63 | 0.0000342 |
| 93 | chr6 | 139695762 | 139695990 | NM_006079 | CITED2 | -89 | -0.95 | 0.0000367 |
| 94 | chr7 | 103848432 | 103848700 | NM_181747 | ORC5 | -71 | -0.75 | 0.0000408 |
| 95 | chr3 | 12705542 | 12705890 | NM_002880 | RAF1 | -16 | -0.71 | 0.0000422 |
| 96 | chr16 | 11836572 | 11836780 | NM_001303447 | TXNDC11 | 58 | -0.63 | 0.0000427 |
| 97 | chr5 | 65018742 | 65018990 | NM_020726 | NLN | 843 | -1.07 | 0.0000436 |
| 98 | chr6 | 148663512 | 148663790 | NM_001346506 | SASH1 | -78 | -0.93 | 0.0000443 |
| 99 | chr15 | 59042212 | 59042400 | NM_001320570 | ADAM10 | -129 | -0.73 | 0.0000468 |
| 100 | chr11 | 113576932 | 113577130 | NM_030770 | TMPRSS5 | 64 | -0.71 | 0.0000468 |
| 101 | chr10 | 75936112 | 75936450 | NM_001202449 | ADK | 34 | -0.72 | 0.0000478 |
| 102 | chr12 | 53473012 | 53473330 | NM_032840 | SPRYD3 | 33 | -0.54 | 0.000049 |
| 103 | chr6 | 36853572 | 36853880 | NM_152734 | C6orf89 | 86 | -0.63 | 0.0000494 |
| 104 | chr8 | 61429422 | 61429660 | NM_001242644 | RAB2A | 72 | -0.8 | 0.0000499 |
| 105 | chr6 | 56717012 | 56717290 | NM_001144770 | DST | -437 | -1.62 | 0.0000509 |
| 106 | chr8 | 130253372 | 130253700 | NR_033916 | LINC00977 | -50 | -0.67 | 0.0000524 |
| 107 | chr2 | 64455202 | 64455500 | NR_120420 | LOC100507006 | -184 | -0.73 | 0.0000549 |
| 108 | chr12 | 32259722 | 32259950 | NM_001003398 | BICD1 | -349 | -0.75 | 0.0000556 |
| 109 | chr7 | 99766192 | 99766720 | NM_024637 | GAL3ST4 | -83 | -0.75 | 0.0000563 |
| 110 | chr4 | 123844062 | 123844350 | NM_001317799 | SPATA5 | -19 | -0.78 | 0.0000563 |
| 111 | chr13 | 41345352 | 41345530 | NM_005830 | MRPS31 | -94 | -0.57 | 0.0000587 |
| 112 | chr8 | 141522032 | 141522270 | NR_040712 | CHRAC1 | 70 | -0.95 | 0.0000612 |
| 113 | chr10 | 6186662 | 6186960 | NM_001145443 | PFKFB3 | -30 | -0.84 | 0.0000615 |
| 114 | chr6 | 36954172 | 36954420 | NM_001271641 | MTCH1 | 31 | -0.98 | 0.0000629 |
| 115 | chr17 | 28088422 | 28088640 | NM_001282131 | SSH2 | -81 | -0.86 | 0.0000657 |
| 116 | chr18 | 44337622 | 44337930 | NM_001307987 | ST8SIA5 | -644 | -1.06 | 0.0000658 |
| 117 | chr20 | 3827232 | 3827540 | NR_037921 | MAVS | -60 | -0.78 | 0.0000668 |
| 118 | chrX | 118925862 | 118926130 | NM_001000 | RPL39 | -374 | -0.96 | 0.0000757 |
| 119 | chr12 | 74931492 | 74931680 | NM_001136262 | ATXN7L3B | 35 | -0.67 | 0.000082 |
| 120 | chr2 | 178129422 | 178129720 | NM_001313903 | NFE2L2 | 288 | -0.9 | 0.0000832 |
| 121 | chr5 | 74532642 | 74532890 | NM_001164443 | ANKRD31 | -63 | -1.19 | 0.0000871 |
| 122 | chr4 | 56413022 | 56413310 | NM_004898 | CLOCK | -90 | -0.66 | 0.000088 |
| 123 | chr11 | 63933412 | 63933630 | NM_014067 | MACROD1 | 64 | -0.76 | 0.0000886 |
| 124 | chr7 | 192222 | 192630 | NM_020223 | FAM20C | -543 | -0.87 | 0.000089 |
| 125 | chr1 | 244615352 | 244615620 | NM_001126 | ADSS | -50 | -0.57 | 0.0000893 |
| 126 | chr12 | 107168282 | 107168500 | NM_018157 | RIC8B | 57 | -0.67 | 0.0000915 |
| 127 | chr1 | 101490462 | 101490790 | NM_001077394 | DPH5 | 736 | -1.23 | 0.0000933 |
| 128 | chr9 | 127962592 | 127962830 | NR_135145 | LOC105376271 | -36 | -0.56 | 0.0000978 |
| 129 | chr17 | 74350192 | 74350470 | NM_001330503 | PRPSAP1 | -101 | -0.67 | 0.0000979 |
| 130 | chr4 | 160188232 | 160188480 | NM_014247 | RAPGEF2 | -642 | -1.21 | 0.0000993 |
| 131 | chr2 | 203130242 | 203130480 | NM_015934 | NOP58 | -78 | -0.71 | 0.000101916 |
| 132 | chr18 | 61089812 | 61090090 | NM_004869 | VPS4B | -199 | -0.55 | 0.000102679 |
| 133 | chr10 | 103911882 | 103912060 | NM_004741 | NOLC1 | 38 | -0.52 | 0.000104742 |
| 134 | chr16 | 19179052 | 19179310 | NM_001330509 | SYT17 | 25 | -0.73 | 0.000109319 |
| 135 | chr7 | 33102282 | 33102470 | NM_001002009 | NT5C3A | 33 | -0.55 | 0.00010971 |
| 136 | chr11 | 6256212 | 6256480 | NM_001098794 | FAM160A2 | -405 | -0.61 | 0.000110854 |
| 137 | chr1 | 182360402 | 182360670 | NM_001033056 | GLUL | 3 | -0.85 | 0.000110881 |
| 138 | chr4 | 41614492 | 41614910 | NM_001330982 | LIMCH1 | -25 | -0.72 | 0.000111421 |
| 139 | chr1 | 224370772 | 224371130 | NM_001321541 | DEGS1 | 41 | -0.7 | 0.000111703 |
| 140 | chr11 | 120434782 | 120435070 | NM_001282470 | GRIK4 | -219 | -0.87 | 0.000111752 |
| 141 | chr1 | 150669552 | 150669910 | NM_018178 | GOLPH3L | -59 | -0.66 | 0.000114763 |
| 142 | chr7 | 45026362 | 45026620 | NR_003697 | SNHG15 | -232 | -0.59 | 0.000114881 |
| 143 | chr21 | 37092812 | 37093180 | NR_030414 | MIR802 | -17 | -1.35 | 0.000115088 |
| 144 | chr1 | 111149022 | 111149290 | NM_004974 | KCNA2 | -181 | -0.79 | 0.000118293 |
| 145 | chr13 | 27746012 | 27746310 | NM_182488 | USP12 | -128 | -0.56 | 0.000126566 |
| 146 | chr5 | 71475152 | 71475700 | NM_001324255 | MAP1B | -29 | -0.82 | 0.000128183 |
| 147 | chr1 | 193074542 | 193074750 | NM_197962 | GLRX2 | -38 | -0.68 | 0.000131132 |
| 148 | chr5 | 86708652 | 86708910 | NM_001199189 | CCNH | -60 | -0.59 | 0.000137306 |
| 149 | chr9 | 80911702 | 80911980 | NM_021154 | PSAT1 | -150 | -0.62 | 0.000139526 |
| 150 | chr9 | 19408812 | 19409060 | NM_001010887 | ACER2 | 11 | -0.67 | 0.000140927 |
| 151 | chr14 | 23845542 | 23846300 | NM_001288746 | CMTM5 | -96 | -0.99 | 0.000144788 |
| 152 | chr10 | 32734952 | 32735190 | NR_109826 | CCDC7 | 61 | -0.89 | 0.000145312 |
| 153 | chr5 | 31532322 | 31532650 | NM_018356 | C5orf22 | 113 | -0.78 | 0.000146498 |
| 154 | chr11 | 805292 | 805560 | NM_145886 | PIDD1 | -176 | -1.03 | 0.000151834 |
| 155 | chr7 | 105752872 | 105753070 | NM_006754 | SYPL1 | 122 | -0.76 | 0.000151892 |
| 156 | chr12 | 46777022 | 46777360 | NR_125380 | LOC100288798 | -268 | -0.75 | 0.000152051 |
| 157 | chr11 | 66726002 | 66726200 | NM_000920 | PC | -254 | -0.57 | 0.00015693 |
| 158 | chr11 | 85779982 | 85780290 | NM_001008660 | PICALM | 3 | -0.67 | 0.000157064 |
| 159 | chr12 | 109221092 | 109221690 | NM_001161331 | SSH1 | -64 | -0.98 | 0.000157553 |
| 160 | chr12 | 30907502 | 30907920 | NM_001319845 | CAPRIN2 | 113 | -0.73 | 0.000160479 |
| 161 | chr12 | 102513852 | 102514100 | NM_017915 | PARPBP | 27 | -0.74 | 0.000162519 |
| 162 | chr4 | 100867722 | 100868050 | NM_001278310 | DNAJB14 | -3 | -0.88 | 0.000172493 |
| 163 | chr2 | 20101642 | 20101930 | NM_001008237 | TTC32 | -39 | -0.87 | 0.00017325 |
| 164 | chr17 | 39844932 | 39845210 | NM_005801 | EIF1 | -56 | -0.64 | 0.000176402 |
| 165 | chr15 | 89456312 | 89456760 | NM_001114614 | MFGE8 | 164 | -0.97 | 0.000185058 |
| 166 | chr11 | 32112392 | 32112710 | NM_002901 | RCN1 | 74 | -0.8 | 0.000191328 |
| 167 | chr6 | 105850982 | 105851290 | NM_002726 | PREP | -137 | -0.75 | 0.000194506 |
| 168 | chr2 | 208634572 | 208634860 | NM_003468 | FZD5 | -573 | -0.55 | 0.000198894 |
| 169 | chr12 | 122710522 | 122710780 | NM_001278304 | DIABLO | -254 | -0.57 | 0.000199503 |
| 170 | chr12 | 122907832 | 122908160 | NM_002956 | CLIP1 | -880 | -0.69 | 0.000208254 |
| 171 | chr8 | 61326082 | 61327040 | NR_103854 | LINC01301 | -184 | -1.5 | 0.00020996 |
| 172 | chr17 | 40897002 | 40897260 | NM_001991 | EZH1 | -17 | -0.85 | 0.000218298 |
| 173 | chr2 | 183731292 | 183731510 | NM_001463 | FRZB | 97 | -0.81 | 0.000222268 |
| 174 | chr2 | 128615512 | 128615750 | NM_004805 | POLR2D | 98 | -1.07 | 0.000227367 |
| 175 | chr11 | 124823712 | 124824060 | NM_025004 | CCDC15 | -131 | -0.61 | 0.000230232 |
| 176 | chr3 | 123680062 | 123680330 | NM_001308317 | CCDC14 | 59 | -0.65 | 0.000236662 |
| 177 | chr1 | 47779652 | 47779910 | NM_001282936 | STIL | 38 | -0.73 | 0.000237699 |
| 178 | chr6 | 143771562 | 143771790 | NM_182503 | ADAT2 | 165 | -0.97 | 0.000238613 |
| 179 | chr3 | 112280742 | 112281010 | NM_017945 | SLC35A5 | 19 | -0.73 | 0.000241996 |
| 180 | chr11 | 77348772 | 77348970 | NM_001311200 | CLNS1A | -20 | -0.63 | 0.000244613 |
| 181 | chr11 | 96076232 | 96076550 | NM_032427 | MAML2 | -47 | -0.67 | 0.000248283 |
| 182 | chr8 | 110552192 | 110552420 | NM_004215 | EBAG9 | 2 | -0.93 | 0.000253443 |
| 183 | chr2 | 198175452 | 198175670 | NM_153697 | ANKRD44 | -40 | -0.64 | 0.000261716 |
| 184 | chr7 | 108166612 | 108166730 | NM_001256007 | PNPLA8 | 91 | -0.53 | 0.000263785 |
| 185 | chr15 | 64673692 | 64673960 | NM_014736 | PCLAF | -117 | -0.73 | 0.000268012 |
| 186 | chr2 | 232329412 | 232329610 | NM_005381 | NCL | -306 | -0.81 | 0.000268429 |
| 187 | chr17 | 49198402 | 49198580 | NM_001130527 | SPAG9 | -265 | -0.9 | 0.000275634 |
| 188 | chr1 | 100715272 | 100715550 | NM_001918 | DBT | -2 | -0.74 | 0.000277713 |
| 189 | chr1 | 249200412 | 249200560 | NM_001017434 | PGBD2 | 44 | -0.91 | 0.000281696 |
| 190 | chr17 | 37607432 | 37607720 | NM_004774 | MED1 | -49 | -0.71 | 0.000283606 |
| 191 | chr19 | 4457802 | 4458130 | NM_025241 | UBXN6 | -175 | -0.86 | 0.000293274 |
| 192 | chr10 | 98346802 | 98347040 | NM_020123 | TM9SF3 | -112 | -0.67 | 0.000299711 |
| 193 | chr2 | 183580662 | 183580920 | NM_018981 | DNAJC10 | 23 | -0.84 | 0.000307746 |
| 194 | chr7 | 121512932 | 121513300 | NM_001206839 | PTPRZ1 | -43 | -0.62 | 0.000327953 |
| 195 | chr7 | 99517052 | 99517330 | NM_033017 | TRIM4 | 32 | -0.75 | 0.000335176 |
| 196 | chr4 | 83822102 | 83822360 | NR_034075 | THAP9-AS1 | -162 | -1.13 | 0.00033633 |
| 197 | chr1 | 70876742 | 70877010 | NM_153742 | CTH | -25 | -0.64 | 0.000350308 |
| 198 | chr5 | 148737542 | 148737740 | NM_001301057 | PCYOX1L | 71 | -0.82 | 0.000351578 |
| 199 | chr17 | 36956102 | 36956380 | NM_003559 | PIP4K2B | -83 | -0.59 | 0.000360182 |
| 200 | chr5 | 112073282 | 112073590 | NM_001127510 | APC | -120 | -0.58 | 0.000360492 |
| 201 | chr2 | 172967322 | 172967520 | NM_004405 | DLX2 | 57 | -0.94 | 0.000364791 |
| 202 | chr2 | 236402582 | 236402760 | NM_014914 | AGAP1 | -62 | -0.7 | 0.000372055 |
| 203 | chr18 | 71959322 | 71959540 | NM_148923 | CYB5A | -180 | -0.64 | 0.000372055 |
| 204 | chr16 | 28936452 | 28936730 | NM_024816 | RABEP2 | -59 | -0.72 | 0.000385825 |
| 205 | chr12 | 120315032 | 120315300 | NM_007174 | CIT | -71 | -0.64 | 0.000394338 |
| 206 | chr19 | 50887482 | 50887680 | NR_046402 | POLD1 | 1 | -0.68 | 0.00039564 |
| 207 | chr11 | 13030682 | 13030930 | NM_001080521 | RASSF10 | -164 | -0.83 | 0.00040272 |
| 208 | chr22 | 24989062 | 24989280 | NM_207644 | LRRC75B | -136 | -0.7 | 0.000425668 |
| 209 | chr2 | 28974512 | 28974750 | NM_002709 | PPP1CB | 5 | -0.72 | 0.000443232 |
| 210 | chr1 | 25942942 | 25943120 | NM_020379 | MAN1C1 | -928 | -0.73 | 0.000446471 |
| 211 | chr6 | 146055942 | 146056150 | NR_038246 | LOC100507557 | 41 | -0.52 | 0.000449898 |
| 212 | chr2 | 197791472 | 197791680 | NM_001321099 | PGAP1 | -121 | -0.7 | 0.000471457 |
| 213 | chr14 | 32546172 | 32546360 | NM_001173 | ARHGAP5 | -229 | -0.81 | 0.000491892 |
| 214 | chr20 | 57617862 | 57618050 | NR_037930 | SLMO2-ATP5E | -55 | -0.89 | 0.000495937 |
| 215 | chr7 | 127291822 | 127292020 | NM_014390 | SND1 | -122 | -0.62 | 0.000504053 |
| 216 | chr1 | 23694652 | 23695080 | NM_030634 | ZNF436 | 13 | -0.71 | 0.000524262 |
| 217 | chr5 | 138210862 | 138211350 | NM_001323988 | CTNNA1 | 17 | -1.01 | 0.000542556 |
| 218 | chr6 | 151187382 | 151187710 | NM_001242768 | MTHFD1L | 77 | -0.64 | 0.000548688 |
| 219 | chr9 | 79792222 | 79792440 | NM_001018038 | VPS13A | -30 | -0.7 | 0.000555214 |
| 220 | chr11 | 12695612 | 12695870 | NM_021961 | TEAD1 | -228 | -0.81 | 0.000563071 |
| 221 | chr21 | 34397392 | 34397610 | NM_005806 | OLIG2 | -715 | -0.94 | 0.000608016 |
| 222 | chr7 | 155089022 | 155089380 | NM_198336 | INSIG1 | -281 | -0.74 | 0.00061267 |
| 223 | chr10 | 99473372 | 99473570 | NM_031484 | MARVELD1 | 6 | -1.07 | 0.000613474 |
| 224 | chr13 | 91999602 | 91999880 | NR_027350 | MIR17HG | -333 | -0.94 | 0.000642305 |
| 225 | chr7 | 21467372 | 21467550 | NR_137166 | SP4 | -191 | -0.54 | 0.000645805 |
| 226 | chr12 | 121124862 | 121125050 | NM_001303628 | MLEC | 29 | -0.61 | 0.000647352 |
| 227 | chr22 | 29138402 | 29138690 | NM_001318316 | HSCB | -122 | -1.1 | 0.000670222 |
| 228 | chr7 | 55594592 | 55594830 | NM_001321251 | VOPP1 | -138 | -0.71 | 0.000678814 |
| 229 | chr3 | 167813622 | 167814080 | NM_001308155 | GOLIM4 | -138 | -0.68 | 0.000680026 |
| 230 | chr9 | 72873912 | 72874140 | NM_015110 | SMC5 | 148 | -0.93 | 0.000680736 |
| 231 | chr4 | 148605142 | 148605460 | NM_138364 | PRMT9 | 80 | -0.76 | 0.000693889 |
| 232 | chr4 | 2264112 | 2264340 | NM_006454 | MXD4 | -487 | -0.56 | 0.000694654 |
| 233 | chr19 | 36605602 | 36605790 | NM_001281 | TBCB | -192 | -1 | 0.00069517 |
| 234 | chr12 | 64237392 | 64237830 | NM_001346201 | SRGAP1 | -922 | -0.59 | 0.00070035 |
| 235 | chr3 | 148709062 | 148709280 | NM_004130 | GYG1 | -24 | -0.83 | 0.000712395 |
| 236 | chr12 | 57824752 | 57824930 | NM_014925 | R3HDM2 | -53 | -0.61 | 0.000718771 |
| 237 | chr1 | 92764192 | 92764390 | NM_024813 | RPAP2 | -231 | -0.65 | 0.000720388 |
| 238 | chr15 | 41785842 | 41786050 | NM_002220 | ITPKA | -110 | -0.83 | 0.00073273 |
| 239 | chr2 | 201753722 | 201753920 | NM_130906 | PPIL3 | 28 | -1.03 | 0.000745916 |
| 240 | chr11 | 31391372 | 31391540 | NM_181706 | DNAJC24 | 79 | -0.75 | 0.000747814 |
| 241 | chr1 | 113932912 | 113933100 | NM_152900 | MAGI3 | -469 | -0.54 | 0.000752231 |
| 242 | chr17 | 77029902 | 77030590 | NM_198593 | C1QTNF1 | -135 | -0.71 | 0.000755381 |
| 243 | chr2 | 27294562 | 27294740 | NM_001134693 | OST4 | -84 | -0.68 | 0.000779268 |
| 244 | chr9 | 131644142 | 131644400 | NM_004059 | KYAT1 | 83 | -0.69 | 0.000792162 |
| 245 | chr9 | 2015102 | 2015290 | NM_003070 | SMARCA2 | -23 | -0.59 | 0.000800492 |
| 246 | chr4 | 20701852 | 20702130 | NM_001330745 | PACRGL | -45 | -0.67 | 0.000804434 |
| 247 | chr12 | 49504582 | 49504780 | NM_001300751 | LMBR1L | 2 | -0.53 | 0.000810613 |
| 248 | chr6 | 36842802 | 36843020 | NM_016059 | PPIL1 | -111 | -0.92 | 0.000819596 |
| 249 | chr3 | 33155352 | 33155550 | NM_006371 | CRTAP | 1 | -0.88 | 0.000838432 |
| 250 | chr9 | 89763362 | 89763580 | NM_001001709 | C9orf170 | -88 | -0.77 | 0.000857663 |
| 251 | chrX | 46432992 | 46433270 | NM_019886 | CHST7 | 9 | -0.75 | 0.000858641 |
| 252 | chr3 | 42845912 | 42846160 | NM_001099669 | HIGD1A | -9 | -0.61 | 0.000867214 |
| 253 | chr7 | 66093282 | 66093490 | NM_153033 | KCTD7 | -482 | -0.69 | 0.000870531 |
| 254 | chr5 | 133304442 | 133304640 | NM_020199 | C5orf15 | -135 | -0.83 | 0.000898974 |
| 255 | chr5 | 126112262 | 126112820 | NM_005573 | LMNB1 | 226 | -0.79 | 0.000922606 |
| 256 | chr2 | 167232592 | 167232790 | NM_002977 | SCN9A | -194 | -0.89 | 0.000937072 |
| 257 | chr17 | 61904732 | 61904920 | NM_002805 | PSMC5 | 56 | -0.69 | 0.000938973 |
| 258 | chr1 | 170043862 | 170043990 | NM_001204514 | KIFAP3 | -47 | -0.52 | 0.000947443 |
| 259 | chr2 | 166810352 | 166810620 | NM_024753 | TTC21B | -138 | -0.52 | 0.001003576 |
| 260 | chr17 | 79670202 | 79670430 | NM_002949 | MRPL12 | -84 | -0.73 | 0.001007422 |
| 261 | chr20 | 32890792 | 32890970 | NM_001322084 | AHCY | -69 | -0.65 | 0.001014109 |
| 262 | chr1 | 23695352 | 23695580 | NR_033690 | ZNF436-AS1 | 2 | -0.6 | 0.001021289 |
| 263 | chr6 | 108582492 | 108582670 | NM_001300929 | SNX3 | -117 | -0.7 | 0.001039212 |
| 264 | chr3 | 171178142 | 171178400 | NM_001161562 | TNIK | -74 | -0.74 | 0.001047997 |
| 265 | chr5 | 142783222 | 142783480 | NM_001204259 | NR3C1 | -97 | -0.77 | 0.001089641 |
| 266 | chr12 | 106976492 | 106976790 | NM_213594 | RFX4 | -44 | -0.77 | 0.001100544 |
| 267 | chr6 | 26538142 | 26538340 | NM_006353 | HMGN4 | -331 | -0.86 | 0.001119622 |
| 268 | chr11 | 85376162 | 85376360 | NR_028027 | CREBZF | -79 | -0.81 | 0.001119622 |
| 269 | chr19 | 4007142 | 4007370 | NM_015897 | PIAS4 | -340 | -0.72 | 0.001127564 |
| 270 | chr1 | 43855432 | 43855640 | NM_015284 | SZT2 | -20 | -0.9 | 0.001128992 |
| 271 | chr3 | 170136542 | 170136820 | NM_005602 | CLDN11 | 28 | -1.02 | 0.001134456 |
| 272 | chr1 | 33116642 | 33116820 | NM_001135255 | RBBP4 | -18 | -0.63 | 0.001144591 |
| 273 | chr15 | 91446742 | 91446980 | NM_006122 | MAN2A2 | 400 | -0.94 | 0.001162101 |
| 274 | chr6 | 19837112 | 19837250 | NM_001546 | ID4 | -420 | -0.85 | 0.001210157 |
| 275 | chr8 | 124428732 | 124428920 | NM_001283027 | WDYHV1 | -139 | -0.79 | 0.001211228 |
| 276 | chr10 | 11206052 | 11206240 | NM_001326348 | CELF2 | -698 | -0.87 | 0.001224703 |
| 277 | chr10 | 25463472 | 25463660 | NM_020752 | GPR158 | -724 | -0.75 | 0.001226873 |
| 278 | chr20 | 62370872 | 62371140 | NM_020062 | SLC2A4RG | -205 | -0.8 | 0.001231989 |
| 279 | chr8 | 67525382 | 67525620 | NM_001080416 | MYBL1 | -17 | -0.68 | 0.001251351 |
| 280 | chr3 | 98312442 | 98312630 | NM_000097 | CPOX | -81 | -0.62 | 0.001255648 |
| 281 | chr1 | 84971802 | 84972050 | NM_001310156 | SPATA1 | -58 | -0.66 | 0.00127081 |
| 282 | chr2 | 3606232 | 3606420 | NR_038431 | RNASEH1-AS1 | 349 | -0.85 | 0.001277009 |
| 283 | chr16 | 75301252 | 75301520 | NM_001170715 | BCAR1 | 565 | -1 | 0.001296893 |
| 284 | chr13 | 30463102 | 30463520 | NR_046510 | LINC00297 | -769 | -1.42 | 0.001324922 |
| 285 | chr2 | 166325952 | 166326190 | NM_001172173 | CSRNP3 | -86 | -0.91 | 0.001372439 |
| 286 | chr16 | 730042 | 730220 | NM_005861 | STUB1 | 20 | -0.84 | 0.001373099 |
| 287 | chr15 | 65133062 | 65133280 | NM_025201 | PLEKHO2 | -911 | -0.78 | 0.001374216 |
| 288 | chr11 | 64072532 | 64072700 | NM_001282451 | ESRRA | -384 | -0.79 | 0.001387016 |
| 289 | chr11 | 17297962 | 17298150 | NM_001330227 | NUCB2 | -209 | -0.69 | 0.00141581 |
| 290 | chrX | 13671232 | 13671440 | NM_152634 | TCEANC | 26 | -0.75 | 0.001442925 |
| 291 | chr9 | 108006702 | 108006880 | NM_001330731 | SLC44A1 | -95 | -0.57 | 0.001443873 |
| 292 | chr1 | 90460562 | 90460740 | NM_181781 | ZNF326 | -3 | -0.62 | 0.00146089 |
| 293 | chr1 | 90098522 | 90098720 | NM_032270 | LRRC8C | -23 | -0.69 | 0.001461021 |
| 294 | chr2 | 182850342 | 182850700 | NM_001080545 | PPP1R1C | -30 | -0.94 | 0.001467916 |
| 295 | chr17 | 17184392 | 17184690 | NM_001316357 | COPS3 | 76 | -0.87 | 0.001480692 |
| 296 | chr11 | 9594872 | 9595100 | NM_003390 | WEE1 | -242 | -0.57 | 0.001496929 |
| 297 | chr5 | 132362112 | 132362350 | NM_001300816 | ZCCHC10 | 65 | -0.67 | 0.001510361 |
| 298 | chr16 | 73126142 | 73126370 | NR_027756 | HCCAT5 | 8 | -1.04 | 0.001546257 |
| 299 | chr5 | 123774802 | 123775040 | NR_125774 | LINC01170 | -708 | -0.79 | 0.001570308 |
| 300 | chr2 | 10262682 | 10262950 | NM_001034 | RRM2 | -47 | -0.57 | 0.001571538 |
| 301 | chr1 | 155293002 | 155293160 | NM_001278229 | RUSC1 | -647 | -0.51 | 0.001572746 |
| 302 | chr2 | 70520502 | 70520700 | NM_001317169 | SNRPG | -11 | -0.73 | 0.001589124 |
| 303 | chr4 | 184365422 | 184365600 | NM_001317343 | CDKN2AIP | -233 | -0.76 | 0.001594872 |
| 304 | chrX | 70585802 | 70585960 | NM_138923 | TAF1 | -208 | -0.6 | 0.001606099 |
| 305 | chr8 | 134309552 | 134309750 | NM_006096 | NDRG1 | -104 | -0.75 | 0.00161003 |
| 306 | chr4 | 154125512 | 154125700 | NM_015271 | TRIM2 | 40 | -0.73 | 0.001618498 |
| 307 | chr6 | 139309482 | 139309670 | NM_031922 | REPS1 | -178 | -0.91 | 0.001623981 |
| 308 | chr3 | 120068072 | 120068280 | NM_001099678 | LRRC58 | 10 | -0.74 | 0.00166614 |
| 309 | chr18 | 11908672 | 11908900 | NM_023075 | MPPE1 | 10 | -0.8 | 0.001678304 |
| 310 | chr1 | 205196822 | 205197010 | NM_014858 | TMCC2 | -122 | -0.93 | 0.001686451 |
| 311 | chr2 | 118771632 | 118771820 | NM_019044 | CCDC93 | 13 | -0.78 | 0.001688093 |
| 312 | chr20 | 19870022 | 19870270 | NM_018993 | RIN2 | -64 | -0.98 | 0.001697109 |
| 313 | chr7 | 86781682 | 86781810 | NR_136253 | LOC101927420 | 63 | -0.53 | 0.001708961 |
| 314 | chr19 | 19496142 | 19496320 | NM_017660 | GATAD2A | -411 | -0.66 | 0.001715653 |
| 315 | chr8 | 90769902 | 90770120 | NM_003821 | RIPK2 | 36 | -0.9 | 0.001720597 |
| 316 | chr17 | 42580792 | 42581000 | NM_001304941 | GPATCH8 | 74 | -0.76 | 0.001745552 |
| 317 | chr5 | 175875162 | 175875320 | NM_014613 | FAF2 | -115 | -0.69 | 0.001752498 |
| 318 | chr19 | 8070542 | 8070790 | NM_001419 | ELAVL1 | -137 | -0.71 | 0.001805672 |
| 319 | chr10 | 43932072 | 43932300 | NR_026693 | ZNF487 | -388 | -0.53 | 0.001815201 |
| 320 | chr9 | 128508962 | 128509150 | NR_024123 | PBX3 | -561 | -0.79 | 0.001857121 |
| 321 | chr16 | 4475422 | 4475610 | NM_005147 | DNAJA3 | -290 | -0.6 | 0.00187197 |
| 322 | chr19 | 41869622 | 41869860 | NM_001098825 | TMEM91 | -130 | -0.89 | 0.001885442 |
| 323 | chr1 | 234614822 | 234615010 | NM_005646 | TARBP1 | -67 | -0.94 | 0.001889311 |
| 324 | chr6 | 160182892 | 160183070 | NM_005891 | ACAT2 | -8 | -0.73 | 0.001897696 |
| 325 | chr20 | 43595082 | 43595280 | NM_006282 | STK4 | 66 | -0.75 | 0.001897771 |
| 326 | chr19 | 44100572 | 44100770 | NM_001145347 | ZNF576 | -67 | -0.72 | 0.001907171 |
| 327 | chr8 | 27169042 | 27169240 | NM_173174 | PTK2B | 142 | -0.81 | 0.001912447 |
| 328 | chr11 | 73693942 | 73694070 | NM_003355 | UCP2 | -117 | -0.75 | 0.001943828 |
| 329 | chr9 | 15422612 | 15422750 | NM_001039697 | SNAPC3 | -101 | -0.59 | 0.001968806 |
| 330 | chr1 | 11994492 | 11994700 | NM_000302 | PLOD1 | -128 | -0.91 | 0.001985154 |
| 331 | chr11 | 134093922 | 134094110 | NM_015261 | NCAPD3 | 410 | -0.76 | 0.002007543 |
| 332 | chr2 | 203499482 | 203499720 | NM_173511 | FAM117B | -300 | -0.54 | 0.002017524 |
| 333 | chr6 | 18155512 | 18155810 | NM_153042 | KDM1B | 42 | -0.63 | 0.002024192 |
| 334 | chr9 | 130159352 | 130159500 | NM_014580 | SLC2A8 | 9 | -0.56 | 0.002062276 |
| 335 | chr19 | 40971812 | 40972020 | NM_000713 | BLVRB | -191 | -0.55 | 0.002073172 |
| 336 | chr10 | 102046282 | 102046520 | NM_173809 | BLOC1S2 | 68 | -0.86 | 0.002127966 |
| 337 | chr2 | 190648742 | 190648950 | NM_001321045 | PMS1 | 35 | -0.7 | 0.002128193 |
| 338 | chr10 | 11574392 | 11574620 | NM_001080491 | USP6NL | -227 | -0.78 | 0.002146803 |
| 339 | chr16 | 70473102 | 70473260 | NM_006927 | ST3GAL2 | -190 | -0.67 | 0.002147376 |
| 340 | chr6 | 44214262 | 44214450 | NM_001271972 | HSP90AB1 | -339 | -0.8 | 0.002159957 |
| 341 | chr8 | 128748202 | 128748320 | NM_002467 | MYC | -54 | -0.57 | 0.002168564 |
| 342 | chr6 | 122720632 | 122720830 | NM_001135564 | HSF2 | 35 | -0.62 | 0.002252158 |
| 343 | chr16 | 54964542 | 54964760 | NM_001252197 | IRX5 | -460 | -0.66 | 0.002262947 |
| 344 | chr5 | 61708412 | 61708620 | NM_016338 | IPO11 | -57 | -0.78 | 0.002263905 |
| 345 | chr2 | 198364482 | 198364680 | NM_002156 | HSPD1 | 59 | -0.62 | 0.002270703 |
| 346 | chr2 | 206547012 | 206547210 | NM_018534 | NRP2 | -113 | -0.71 | 0.002290234 |
| 347 | chr2 | 120124302 | 120124490 | NM_001322332 | C2orf76 | 8 | -0.63 | 0.002316797 |
| 348 | chr5 | 68389592 | 68389840 | NM_001251969 | SLC30A5 | -60 | -0.57 | 0.002333522 |
| 349 | chr5 | 67583772 | 67584030 | NM_181524 | PIK3R1 | -351 | -0.71 | 0.002387566 |
| 350 | chr3 | 185303682 | 185303870 | NM_021627 | SENP2 | -255 | -0.65 | 0.002416315 |
| 351 | chr10 | 62704022 | 62704190 | NM_014836 | RHOBTB1 | -73 | -0.91 | 0.002433773 |
| 352 | chr4 | 128801872 | 128802040 | NM_001190799 | PLK4 | -60 | -0.77 | 0.002459002 |
| 353 | chr3 | 101568202 | 101568380 | NM_031419 | NFKBIZ | -67 | -0.6 | 0.002501303 |
| 354 | chr14 | 77564192 | 77564360 | NM_033426 | CIPC | -302 | -0.61 | 0.002501303 |
| 355 | chr1 | 68962862 | 68963080 | NM_001114120 | DEPDC1 | -67 | -0.58 | 0.002522867 |
| 356 | chr22 | 42343082 | 42343250 | NM_001002876 | CENPM | 2 | -0.68 | 0.00256495 |
| 357 | chr7 | 102715452 | 102715660 | NM_001161011 | ARMC10 | 228 | -0.56 | 0.002644538 |
| 358 | chr2 | 179345042 | 179345260 | NM_019091 | PLEKHA3 | -48 | -0.63 | 0.002744321 |
| 359 | chr19 | 45926882 | 45927070 | NM_202001 | ERCC1 | -156 | -0.8 | 0.002770366 |
| 360 | chr19 | 41222912 | 41223080 | NM_025194 | ITPKC | -12 | -0.71 | 0.002853331 |
| 361 | chr14 | 55518092 | 55518250 | NM_144578 | MAPK1IP1L | -191 | -0.78 | 0.002855461 |
| 362 | chr12 | 62997162 | 62997340 | NR_121682 | LINC01465 | -37 | -0.85 | 0.002861914 |
| 363 | chr14 | 54863662 | 54863800 | NM_005192 | CDKN3 | 58 | -0.6 | 0.002946911 |
| 364 | chr2 | 128284322 | 128284550 | NM_017969 | IWS1 | -349 | -0.64 | 0.003040373 |
| 365 | chr6 | 41909482 | 41909830 | NM_001136125 | CCND3 | -70 | -0.81 | 0.003047212 |
| 366 | chr7 | 91763692 | 91763860 | NM_000786 | CYP51A1 | 64 | -0.61 | 0.00306552 |
| 367 | chr11 | 86748692 | 86748880 | NM_001168724 | TMEM135 | -100 | -0.83 | 0.003104344 |
| 368 | chr3 | 72937212 | 72937450 | NR_138564 | GXYLT2 | -54 | -0.85 | 0.00311 |
| 369 | chr22 | 18560682 | 18560890 | NM_001127649 | PEX26 | 26 | -0.58 | 0.003127753 |
| 370 | chr2 | 232379032 | 232379220 | NR_024079 | LINC00471 | -76 | -0.55 | 0.00316917 |
| 371 | chr8 | 22298362 | 22298540 | NM_001243974 | PPP3CC | -32 | -0.76 | 0.003249428 |
| 372 | chr17 | 74553602 | 74553740 | NR_038110 | SNHG16 | -175 | -0.64 | 0.003262776 |
| 373 | chr9 | 5437912 | 5438090 | NM_018465 | PLGRKT | -64 | -0.68 | 0.003269143 |
| 374 | chr1 | 161102222 | 161102400 | NM_001039712 | DEDD | -55 | -0.74 | 0.00333816 |
| 375 | chr2 | 46844152 | 46844340 | NM_173074 | PIGF | 5 | -0.57 | 0.003408072 |
| 376 | chr9 | 140100062 | 140100260 | NM_001144027 | NDOR1 | 42 | -0.84 | 0.003419433 |
| 377 | chr12 | 21810702 | 21810860 | NM_001315537 | LDHB | 8 | -0.64 | 0.003463711 |
| 378 | chr5 | 167913322 | 167913460 | NM_002887 | RARS | -72 | -0.7 | 0.00346677 |
| 379 | chr7 | 26241072 | 26241260 | NM_016587 | CBX3 | -158 | -0.59 | 0.003501677 |
| 380 | chr3 | 5229282 | 5229500 | NM_014674 | EDEM1 | 32 | -0.61 | 0.003504822 |
| 381 | chr11 | 67888812 | 67889000 | NM_001277 | CHKA | -48 | -0.63 | 0.003557814 |
| 382 | chr14 | 97263482 | 97263660 | NM_003384 | VRK1 | -113 | -0.61 | 0.003573339 |
| 383 | chr12 | 133263822 | 133264030 | NM_006231 | POLE | 184 | -0.75 | 0.003583854 |
| 384 | chr18 | 55289012 | 55289190 | NM_004539 | NARS | 76 | -0.64 | 0.003584579 |
| 385 | chr3 | 136537732 | 136537890 | NM_001097600 | SLC35G2 | -50 | -0.94 | 0.003586448 |
| 386 | chr7 | 66205372 | 66205600 | NM_001287062 | RABGEF1 | -157 | -0.6 | 0.003636488 |
| 387 | chr8 | 41386622 | 41386810 | NM_032336 | GINS4 | -9 | -0.54 | 0.003666508 |
| 388 | chr12 | 51420312 | 51420510 | NR_033422 | SLC11A2 | -212 | -0.77 | 0.003677094 |
| 389 | chr12 | 4430012 | 4430240 | NM_020375 | TIGAR | -233 | -0.56 | 0.003691193 |
| 390 | chr14 | 21537602 | 21537820 | NM_001278529 | ARHGEF40 | -708 | -0.62 | 0.003754072 |
| 391 | chr6 | 117002222 | 117002380 | NM_002269 | KPNA5 | -66 | -0.64 | 0.003757817 |
| 392 | chr22 | 21368432 | 21368620 | NR_003608 | TUBA3FP | 50 | -0.63 | 0.003779344 |
| 393 | chr19 | 7459832 | 7460080 | NM_015318 | ARHGEF18 | -43 | -0.7 | 0.003785496 |
| 394 | chr3 | 101280512 | 101280660 | NM_017819 | TRMT10C | -94 | -0.95 | 0.00378588 |
| 395 | chr1 | 202311152 | 202311340 | NM_001310326 | UBE2T | -152 | -0.59 | 0.003803461 |
| 396 | chr19 | 4400452 | 4400610 | NM_003025 | SH3GL1 | 34 | -0.81 | 0.003824465 |
| 397 | chr22 | 47015812 | 47016320 | NM_015124 | GRAMD4 | -233 | -0.8 | 0.003987884 |
| 398 | chr18 | 55711162 | 55711320 | NM_015277 | NEDD4L | -369 | -0.78 | 0.003999184 |
| 399 | chr12 | 102455712 | 102455940 | NM_016053 | WASHC3 | 76 | -0.72 | 0.00402669 |
| 400 | chr14 | 64854682 | 64854830 | NM_005956 | MTHFD1 | -3 | -0.66 | 0.00404323 |
| 401 | chr6 | 136610842 | 136610990 | NM_014739 | BCLAF1 | 73 | -0.74 | 0.004060147 |
| 402 | chr14 | 61447752 | 61447980 | NM_001172702 | SLC38A6 | 34 | -0.62 | 0.004092448 |
| 403 | chr5 | 118324232 | 118324440 | NM_173666 | DTWD2 | -36 | -0.64 | 0.00417425 |
| 404 | chr6 | 43597122 | 43597310 | NM_001003690 | MAD2L1BP | -63 | -0.57 | 0.004182707 |
| 405 | chr1 | 43824242 | 43824440 | NM_001255 | CDC20 | -285 | -0.78 | 0.004183518 |
| 406 | chr2 | 203879162 | 203879370 | NM_001114132 | NBEAL1 | -336 | -0.55 | 0.004192077 |
| 407 | chr12 | 22778062 | 22778250 | NM_001039481 | ETNK1 | 80 | -0.54 | 0.004226329 |
| 408 | chr5 | 32174302 | 32174570 | NM_022130 | GOLPH3 | -11 | -0.65 | 0.004257837 |
| 409 | chr8 | 8860152 | 8860330 | NM_153332 | ERI1 | -73 | -0.78 | 0.004265414 |
| 410 | chr9 | 35080142 | 35080290 | NM_004629 | FANCG | -203 | -0.89 | 0.004284223 |
| 411 | chr1 | 27247912 | 27248220 | NM_006600 | NUDC | -147 | -0.54 | 0.004288107 |
| 412 | chr11 | 110300472 | 110300680 | NM_004109 | FDX1 | -85 | -0.94 | 0.004333231 |
| 413 | chr5 | 110848372 | 110848530 | NM_139164 | STARD4 | -156 | -0.58 | 0.004389983 |
| 414 | chr3 | 57678292 | 57678450 | NM_152678 | DENND6A | 445 | -0.85 | 0.004396686 |
| 415 | chr13 | 38923552 | 38923720 | NM_001286704 | UFM1 | -272 | -0.84 | 0.004402279 |
| 416 | chr2 | 154728062 | 154728250 | NM_052917 | GALNT13 | -255 | -0.76 | 0.004461943 |
| 417 | chr9 | 101867052 | 101867200 | NM_004612 | TGFBR1 | -245 | -0.61 | 0.004506099 |
| 418 | chr2 | 106227682 | 106227890 | NR_038891 | LOC285000 | -770 | -0.99 | 0.004514659 |
| 419 | chr5 | 32313022 | 32313180 | NM_001294343 | MTMR12 | 13 | -0.58 | 0.004522883 |
| 420 | chr11 | 65122092 | 65122260 | NM_145719 | TIGD3 | -106 | -0.62 | 0.004531209 |
| 421 | chr5 | 159848662 | 159848890 | NM_004219 | PTTG1 | -38 | -0.7 | 0.004595143 |
| 422 | chr11 | 8932612 | 8932780 | NM_001206647 | AKIP1 | -5 | -0.7 | 0.004597727 |
| 423 | chr16 | 5148342 | 5148530 | NM_201400 | EEF2KMT | -615 | -0.73 | 0.004611911 |
| 424 | chr16 | 3019072 | 3019230 | NM_001284512 | PAQR4 | -95 | -0.65 | 0.004624181 |
| 425 | chr6 | 166755982 | 166756160 | NM_145169 | SFT2D1 | 23 | -0.7 | 0.004637063 |
| 426 | chr6 | 126101442 | 126101710 | NM_001199619 | NCOA7 | -731 | -0.63 | 0.004640769 |
| 427 | chr15 | 64995482 | 64995630 | NM_002537 | OAZ2 | -76 | -0.67 | 0.004683999 |
| 428 | chr11 | 117015002 | 117015130 | NM_001184747 | PAFAH1B2 | 66 | -0.77 | 0.004722703 |
| 429 | chr11 | 107992232 | 107992420 | NM_000019 | ACAT1 | 68 | -0.69 | 0.004788247 |
| 430 | chrX | 85302432 | 85302630 | NM_001145414 | CHM | 38 | -0.81 | 0.004805129 |
| 431 | chr12 | 52430722 | 52430950 | NM_001202234 | NR4A1 | -663 | -0.76 | 0.004882856 |
| 432 | chr18 | 13726432 | 13726610 | NM_152352 | FAM210A | 70 | -0.68 | 0.004908989 |
| 433 | chr17 | 72983592 | 72983800 | NM_014603 | CDR2L | -31 | -0.52 | 0.004932353 |
| 434 | chr1 | 184356152 | 184356330 | NM_030806 | C1orf21 | 91 | -0.82 | 0.004932771 |
| 435 | chr2 | 176866942 | 176867090 | NM_001305010 | LNPK | 57 | -0.6 | 0.004957794 |
| 436 | chrX | 13752752 | 13752960 | NM_003611 | OFD1 | 24 | -0.64 | 0.004961567 |
| 437 | chr1 | 24969272 | 24969460 | NM_001303448 | SRRM1 | -228 | -0.74 | 0.005003132 |
| 438 | chr2 | 33824482 | 33824640 | NM_015475 | FAM98A | -132 | -0.61 | 0.005007746 |
| 439 | chr13 | 103498082 | 103498300 | NM_000123 | ERCC5 | 0 | -0.75 | 0.005014173 |
| 440 | chr16 | 48644172 | 48644360 | NM_153029 | N4BP1 | -146 | -0.83 | 0.005069586 |
| 441 | chr4 | 166128622 | 166128820 | NM_007246 | KLHL2 | -49 | -0.74 | 0.005079142 |
| 442 | chr3 | 13521032 | 13521220 | NR_046690 | HDAC11-AS1 | 427 | -0.74 | 0.005088695 |
| 443 | chr8 | 17354462 | 17354660 | NM_001008539 | SLC7A2 | -36 | -0.69 | 0.005202397 |
| 444 | chr8 | 37962952 | 37963140 | NM_001282272 | ASH2L | 35 | -0.68 | 0.005300318 |
| 445 | chr16 | 2021782 | 2021980 | NM_006453 | TBL3 | -183 | -0.62 | 0.005339225 |
| 446 | chr8 | 143696772 | 143696890 | NM_015193 | ARC | -998 | -0.68 | 0.005392941 |
| 447 | chr5 | 10353562 | 10353790 | NM_005885 | MARCH6 | -75 | -0.67 | 0.005431503 |
| 448 | chr1 | 220701472 | 220701660 | NM_001286128 | MARK1 | 41 | -0.97 | 0.005470498 |
| 449 | chr9 | 98079662 | 98079800 | NM_001243743 | FANCC | -195 | -0.84 | 0.005487343 |
| 450 | chr9 | 130478002 | 130478120 | NM_001345978 | PTRH1 | -106 | -0.6 | 0.005502439 |
| 451 | chr13 | 37633792 | 37634020 | NM_001278481 | SUPT20H | -56 | -0.64 | 0.005536799 |
| 452 | chr2 | 208394622 | 208394730 | NM_004379 | CREB1 | 60 | -0.51 | 0.0056143 |
| 453 | chr2 | 11484912 | 11485070 | NM_004850 | ROCK2 | -280 | -0.78 | 0.005642331 |
| 454 | chr9 | 131486462 | 131486660 | NM_001318023 | ZDHHC12 | -153 | -0.69 | 0.005643339 |
| 455 | chr7 | 44163342 | 44163500 | NM_001127218 | POLD2 | -252 | -0.68 | 0.005659336 |
| 456 | chr2 | 70529162 | 70529310 | NM_001329755 | FAM136A | -16 | -0.8 | 0.005666525 |
| 457 | chr16 | 28962082 | 28962270 | NM_032815 | NFATC2IP | -142 | -0.51 | 0.005693421 |
| 458 | chr15 | 44719332 | 44719450 | NM_016396 | CTDSPL2 | -188 | -0.57 | 0.005723595 |
| 459 | chr6 | 123100462 | 123100590 | NM_001319039 | FABP7 | -94 | -0.81 | 0.005740788 |
| 460 | chr7 | 97501782 | 97501980 | NM_183356 | ASNS | -27 | -0.57 | 0.005806336 |
| 461 | chr3 | 179040942 | 179041100 | NM_001303425 | ZNF639 | 242 | -0.92 | 0.005821808 |
| 462 | chr6 | 99963202 | 99963380 | NM_001346029 | USP45 | -39 | -0.71 | 0.005833914 |
| 463 | chr5 | 16465812 | 16465980 | NM_033414 | ZNF622 | -2 | -0.83 | 0.005839022 |
| 464 | chr9 | 21974842 | 21974970 | NM_058197 | CDKN2A | -80 | -0.72 | 0.005873873 |
| 465 | chr3 | 167452732 | 167452830 | NM_145859 | PDCD10 | -130 | -0.61 | 0.005909395 |
| 466 | chr17 | 71228222 | 71228390 | NM_001288771 | C17orf80 | -66 | -0.79 | 0.005938232 |
| 467 | chr2 | 191745462 | 191745570 | NM_001256310 | GLS | -31 | -0.6 | 0.00601131 |
| 468 | chr3 | 57261642 | 57261760 | NM_012096 | APPL1 | -64 | -0.79 | 0.006111374 |
| 469 | chr6 | 52149512 | 52149680 | NM_002388 | MCM3 | 83 | -0.6 | 0.006216381 |
| 470 | chr8 | 37552902 | 37553070 | NM_025069 | ZNF703 | -283 | -0.77 | 0.006235705 |
| 471 | chr14 | 24605302 | 24605470 | NM_001281529 | PSME1 | 19 | -0.57 | 0.006244881 |
| 472 | chr5 | 109024952 | 109025130 | NM_002372 | MAN2A1 | -26 | -0.66 | 0.00625446 |
| 473 | chr1 | 201857372 | 201857640 | NR_030775 | SHISA4 | -291 | -0.6 | 0.006453942 |
| 474 | chr1 | 51434192 | 51434410 | NM_001262 | CDKN2C | -66 | -0.66 | 0.006513812 |
| 475 | chr1 | 47133992 | 47134150 | NM_022745 | ATPAF1 | 28 | -0.74 | 0.006513985 |
| 476 | chr1 | 236849522 | 236849700 | NM_001278343 | ACTN2 | -143 | -0.78 | 0.006530474 |
| 477 | chr17 | 27169712 | 27169870 | NM_001077498 | FAM222B | 66 | -0.75 | 0.006561847 |
| 478 | chr4 | 177116772 | 177116960 | NM_144644 | SPATA4 | -44 | -0.85 | 0.006600056 |
| 479 | chr12 | 107380752 | 107381120 | NM_025198 | MTERF2 | 8 | -0.57 | 0.006622331 |
| 480 | chr7 | 99036852 | 99037000 | NM_001318162 | CPSF4 | -73 | -0.72 | 0.006689849 |
| 481 | chr12 | 56660802 | 56660950 | NM_001099337 | COQ10A | -191 | -0.64 | 0.006734999 |
| 482 | chr2 | 9144022 | 9144200 | NR_135599 | MBOAT2 | -232 | -0.71 | 0.006789878 |
| 483 | chr9 | 33290182 | 33290370 | NM_001318758 | NFX1 | -139 | -0.76 | 0.00688451 |
| 484 | chr11 | 3819032 | 3819210 | NR_045925 | PGAP2 | 72 | -0.51 | 0.006913299 |
| 485 | chr3 | 152016382 | 152016690 | NM_207297 | MBNL1 | -658 | -0.55 | 0.006983744 |
| 486 | chr4 | 154265602 | 154265960 | NR_045605 | MND1 | -20 | -0.82 | 0.006992403 |
| 487 | chr15 | 66648932 | 66649100 | NM_017858 | TIPIN | 38 | -0.78 | 0.006992901 |
| 488 | chr7 | 151216952 | 151217130 | NM_005614 | RHEB | -31 | -0.52 | 0.007093782 |
| 489 | chr5 | 172261202 | 172261390 | NM_001031711 | ERGIC1 | 73 | -0.84 | 0.007213749 |
| 490 | chr19 | 2427842 | 2428010 | NM_012458 | TIMM13 | -12 | -0.57 | 0.007245573 |
| 491 | chr6 | 90539282 | 90539520 | NM_001137668 | CASP8AP2 | -218 | -0.53 | 0.007256478 |
| 492 | chr12 | 123319342 | 123319520 | NM_001303099 | HIP1R | 169 | -0.63 | 0.007283793 |
| 493 | chr5 | 43192152 | 43192360 | NM_153361 | NIM1K | 86 | -0.62 | 0.007491623 |
| 494 | chr19 | 33071872 | 33072030 | NM_004708 | PDCD5 | -143 | -0.67 | 0.00749285 |
| 495 | chr2 | 27440232 | 27440380 | NM_001306079 | CAD | 48 | -0.79 | 0.007518598 |
| 496 | chr9 | 77703272 | 77703390 | NM_012383 | OSTF1 | -67 | -0.71 | 0.00764173 |
| 497 | chr10 | 91403782 | 91403960 | NM_148978 | PANK1 | -223 | -0.73 | 0.00765427 |
| 498 | chr8 | 33330642 | 33330860 | NM_032664 | FUT10 | -87 | -0.72 | 0.007656305 |
| 499 | chrX | 122993802 | 122993930 | NM_001167 | XIAP | -151 | -0.75 | 0.007700682 |
| 500 | chr16 | 3507782 | 3507930 | NM_024845 | NAA60 | -129 | -0.66 | 0.007753557 |

**Supplementary Table S2. Top 25 candidate transcription complexes enriched within reduced chromatin accessibility regions at proximal promoters upon *H2AZ2* KD.**

| Gene symbol | ReMap enrichment analysis for ATAC-Seq peaks | | | Survival correlation in glioma patients (TCGA) | |
| --- | --- | --- | --- | --- | --- |
|  | Observed Overlap | Expected | -LOG10 E-value | Higher gene expression correlated with worse patient clinical outcome | Wald p-value |
| E2F4 | 1071 | 11 | 1670.9 | yes | 2.24E-20 |
| HBP1 | 952 | 7 | 1642.2 | yes | 2.79E-20 |
| CEBPD | 1016 | 13 | 1498.7 | yes | 6.49E-25 |
| KLF5 | 1006 | 13 | 1459.5 | yes | 6.27E-09 |
| CREM | 1100 | 21 | 1435.4 | yes | 5.44E-15 |
| NFKB1 | 947 | 12 | 1406.5 | yes | 5.40E-17 |
| EGR1 | 954 | 13 | 1379.6 | yes | 1.56E-11 |
| SRF | 897 | 10 | 1375.3 | yes | 5.63E-06 |
| BRCA1 | 851 | 8 | 1349.3 | yes | 2.82E-30 |
| EGLN2 | 1063 | 22 | 1346.2 | yes | 7.38E-12 |
| CDK6 | 897 | 11 | 1338.1 | yes | 9.84E-24 |
| PML | 950 | 16 | 1289.2 | yes | 1.14E-20 |
| SOX9 | 729 | 5 | 1286.6 | yes | 8.76E-13 |
| STAT1 | 1072 | 26 | 1281.6 | yes | 7.81E-20 |
| SIX5 | 756 | 6 | 1280.8 | yes | 1.67E-15 |
| SUZ12 | 1011 | 21 | 1267.0 | yes | 2.67E-06 |
| TRIM24 | 881 | 13 | 1246.3 | yes | 1.12E-09 |
| IRF2 | 753 | 6 | 1241.1 | yes | 3.73E-17 |
| E2F6 | 1031 | 25 | 1237.3 | yes | 2.63E-26 |
| USF1 | 1034 | 26 | 1221.4 | yes | 8.23E-06 |
| RB1 | 720 | 6 | 1207.6 | yes | 7.81E-10 |
| E2F1 | 928 | 18 | 1201.7 | yes | 2.79E-26 |
| ELK1 | 630 | 3 | 1170.9 | yes | 3.80E-18 |
| BHLHE40 | 981 | 24 | 1167.5 | yes | 3.48E-17 |
| MAF | 962 | 22 | 1166.8 | yes | 8.90E-06 |

**Supplementary Table S3. High confidence H2AZ2-associated genes, which are downregulated upon *H2AZ2* KD, and harbor E2F1 and STAT3 binding sites in their gene promoters in GBM cells.**

| **Gene symbol** | **Associated with H2AZ binding sites**  **(±1kb from TSS)** | **Reduced chromatin accessibility upon *H2AZ2* KD (±1 kb from TSS)** | **Down-regulated upon *H2AZ2* KD** | **E2F1 target in U87 GBM cells** | **STAT3 target in U87 GBM cells** | **Established role in cell proliferation** | **Established role in cell migration and invasion** |
| --- | --- | --- | --- | --- | --- | --- | --- |
| MAN2A1 | ✓ | ✓ | ✓ |  | ✓ |  |  |
| PITPNC1 | ✓ | ✓ | ✓ |  | ✓ |  |  |
| GALNT13 | ✓ | ✓ | ✓ | ✓ | ✓ | ✓ |  |
| CTNNAL1 | ✓ | ✓ | ✓ | ✓ | ✓ | ✓ |  |
| ACTN1 | ✓ | ✓ | ✓ | ✓ | ✓ | ✓ | ✓ |
| NEDD4L | ✓ | ✓ | ✓ | ✓ | ✓ |  |  |
| ADK | ✓ | ✓ | ✓ | ✓ | ✓ |  |  |
| OPA1 | ✓ | ✓ | ✓ | ✓ | ✓ |  |  |
| VRK1 | ✓ | ✓ | ✓ | ✓ | ✓ | ✓ | ✓ |
| SOCS2 | ✓ | ✓ | ✓ | ✓ | ✓ |  |  |
| HEY2 | ✓ | ✓ | ✓ | ✓ | ✓ |  |  |
| CENPJ | ✓ | ✓ | ✓ | ✓ | ✓ | ✓ |  |
| UGP2 | ✓ | ✓ | ✓ | ✓ | ✓ |  |  |
| EML2 | ✓ | ✓ | ✓ | ✓ | ✓ |  |  |
| ERI1 | ✓ | ✓ | ✓ | ✓ | ✓ |  |  |
| ARMC10 | ✓ | ✓ | ✓ | ✓ | ✓ | ✓ |  |
| MAN1C1 | ✓ | ✓ | ✓ | ✓ | ✓ |  |  |
| ARHGAP26 | ✓ | ✓ | ✓ | ✓ | ✓ | ✓ | ✓ |
| IGF2BP2 | ✓ | ✓ | ✓ | ✓ | ✓ | ✓ | ✓ |
| **H2AZ2** | **✓** | **✓** | **✓** | **✓** | **✓** | **✓** | **✓** |
| AGAP1 | ✓ | ✓ | ✓ | ✓ |  |  |  |
| KANK1 | ✓ | ✓ | ✓ | ✓ |  |  |  |
| MIER1 | ✓ | ✓ | ✓ | ✓ |  |  |  |
| SSBP3 | ✓ | ✓ | ✓ | ✓ |  |  |  |
| FGF14 | ✓ | ✓ | ✓ | ✓ |  |  |  |
| VCL | ✓ | ✓ | ✓ | ✓ |  |  | ✓ |
| GLS | ✓ | ✓ | ✓ | ✓ |  | ✓ | ✓ |
| TEAD1 | ✓ | ✓ | ✓ | ✓ |  | ✓ |  |
| DUSP16 | ✓ | ✓ | ✓ | ✓ |  | ✓ |  |
| LIMCH1 | ✓ | ✓ | ✓ | ✓ |  |  |  |
| RHOBTB1 | ✓ | ✓ | ✓ | ✓ |  |  |  |
| ALDH1L2 | ✓ | ✓ | ✓ | ✓ |  |  |  |
| ECE1 | ✓ | ✓ | ✓ | ✓ |  | ✓ |  |
| TNIK | ✓ | ✓ | ✓ | ✓ |  | ✓ |  |
| GOLIM4 | ✓ | ✓ | ✓ | ✓ |  |  |  |
| TRIM24 | ✓ | ✓ | ✓ | ✓ |  | ✓ | ✓ |
| STK11IP | ✓ | ✓ | ✓ | ✓ |  |  |  |
| CDC14A | ✓ | ✓ | ✓ | ✓ |  | ✓ |  |
| RAPGEF2 | ✓ | ✓ | ✓ | ✓ |  | ✓ |  |
| PREP | ✓ | ✓ | ✓ | ✓ |  |  |  |
| POLE | ✓ | ✓ | ✓ | ✓ |  | ✓ |  |
| APC | ✓ | ✓ | ✓ | ✓ |  | ✓ |  |
| RHEB | ✓ | ✓ | ✓ | ✓ |  | ✓ | ✓ |
| IL1RAP | ✓ | ✓ | ✓ | ✓ |  |  |  |
| EPB41L2 | ✓ | ✓ | ✓ | ✓ |  |  |  |
| PTK2B | ✓ | ✓ | ✓ | ✓ |  |  | ✓ |
| OSBPL3 | ✓ | ✓ | ✓ | ✓ |  | ✓ | ✓ |
| FAM126A | ✓ | ✓ | ✓ | ✓ |  |  |  |
| DTWD2 | ✓ | ✓ | ✓ | ✓ |  |  |  |
| DST | ✓ | ✓ | ✓ | ✓ |  |  |  |
| PRKCE | ✓ | ✓ | ✓ | ✓ |  |  |  |
| C1GALT1 | ✓ | ✓ | ✓ | ✓ |  | ✓ |  |
| TRIM2 | ✓ | ✓ | ✓ | ✓ |  | ✓ | ✓ |
| PUS7 | ✓ | ✓ | ✓ | ✓ |  | ✓ |  |
| CTNNBIP1 | ✓ | ✓ | ✓ | ✓ |  |  |  |
| USP42 | ✓ | ✓ | ✓ | ✓ |  |  |  |
| ADAM10 | ✓ | ✓ | ✓ | ✓ |  | ✓ | ✓ |
| ANKRD44 | ✓ | ✓ | ✓ | ✓ |  |  |  |
| MAGI3 | ✓ | ✓ | ✓ | ✓ |  |  |  |
| ROCK2 | ✓ | ✓ | ✓ | ✓ |  | ✓ | ✓ |
| R3HDM2 | ✓ | ✓ | ✓ | ✓ |  |  |  |
| AFG3L2 | ✓ | ✓ | ✓ | ✓ |  | ✓ |  |
| ACACA | ✓ | ✓ | ✓ | ✓ |  | ✓ | ✓ |
| HSF2 | ✓ | ✓ | ✓ | ✓ |  | ✓ |  |
| ERCC5 | ✓ | ✓ | ✓ | ✓ |  |  |  |
| HIPK3 | ✓ | ✓ | ✓ | ✓ |  | ✓ | ✓ |
| SNX8 | ✓ | ✓ | ✓ | ✓ |  |  |  |
| CCDC14 | ✓ | ✓ | ✓ | ✓ |  |  |  |
| FBXW11 | ✓ | ✓ | ✓ | ✓ |  | ✓ |  |
| SRBD1 | ✓ | ✓ | ✓ | ✓ |  |  |  |
| RRAGC | ✓ | ✓ | ✓ | ✓ |  |  |  |
| RRM1 | ✓ | ✓ | ✓ | ✓ |  | ✓ |  |
| PPP2R2B | ✓ | ✓ | ✓ | ✓ |  |  |  |
| ESR2 | ✓ | ✓ | ✓ | ✓ |  |  |  |
| ZHX1 | ✓ | ✓ | ✓ | ✓ |  | ✓ | ✓ |
| GPD2 | ✓ | ✓ | ✓ | ✓ |  |  |  |
| PHF14 | ✓ | ✓ | ✓ | ✓ |  | ✓ | ✓ |
| SIAH2 | ✓ | ✓ | ✓ | ✓ |  |  |  |
| MBOAT2 | ✓ | ✓ | ✓ | ✓ |  |  |  |
| FTO | ✓ | ✓ | ✓ | ✓ |  |  | ✓ |
| TMEM131 | ✓ | ✓ | ✓ | ✓ |  |  |  |
| CCM2 | ✓ | ✓ | ✓ | ✓ |  |  |  |
| ARPC3 | ✓ | ✓ | ✓ | ✓ |  |  |  |
| TULP3 | ✓ | ✓ | ✓ | ✓ |  |  |  |
| GLYCTK | ✓ | ✓ | ✓ | ✓ |  |  |  |
| ERCC4 | ✓ | ✓ | ✓ | ✓ |  |  |  |
| GIT2 | ✓ | ✓ | ✓ | ✓ |  |  |  |
| CEP350 | ✓ | ✓ | ✓ | ✓ |  |  |  |
| IQCB1 | ✓ | ✓ | ✓ |  |  |  |  |
| C9orf170 | ✓ | ✓ | ✓ |  |  |  |  |
| LTBP1 | ✓ | ✓ | ✓ |  |  |  | ✓ |
| RAPH1 | ✓ | ✓ | ✓ |  |  |  |  |
| GSK3B | ✓ | ✓ | ✓ |  |  |  |  |
| IPO11 | ✓ | ✓ | ✓ |  |  | ✓ |  |
| GNE | ✓ | ✓ | ✓ |  |  | ✓ |  |
| NAV2 | ✓ | ✓ | ✓ |  |  | ✓ | ✓ |
| SNX3 | ✓ | ✓ | ✓ |  |  |  |  |
| RAF1 | ✓ | ✓ | ✓ |  |  | ✓ | ✓ |
| HABP4 | ✓ | ✓ | ✓ |  |  |  |  |

**Supplementary Table S4. Gene lists used for Connectivity Map Analysis.**

| Gene correlated with H2AZ2 in TCGA glioma patient cohort | Pearson Corr. Coefficient | p-value | L1000CDS2 database gene list input type |
| --- | --- | --- | --- |
| H2AZ2 | 1.00 | 0 | "down-regulated" |
| KLHL7 | 0.63 | 2.52E-76 | "down-regulated" |
| SP4 | 0.62 | 1.00E-73 | "down-regulated" |
| TMPO | 0.62 | 4.02E-71 | "down-regulated" |
| ING3 | 0.61 | 6.87E-69 | "down-regulated" |
| FIGNL1 | 0.61 | 2.44E-68 | "down-regulated" |
| ZNF107 | 0.60 | 2.53E-66 | "down-regulated" |
| DBF4 | 0.59 | 5.22E-65 | "down-regulated" |
| ZNF92 | 0.59 | 4.51E-64 | "down-regulated" |
| CKAP2 | 0.59 | 8.01E-64 | "down-regulated" |
| CBX3 | 0.59 | 8.76E-63 | "down-regulated" |
| LIN9 | 0.58 | 4.86E-62 | "down-regulated" |
| NCAPG2 | 0.58 | 1.11E-61 | "down-regulated" |
| ATAD2 | 0.58 | 5.90E-61 | "down-regulated" |
| RSBN1L | 0.58 | 2.33E-60 | "down-regulated" |
| RACGAP1 | 0.57 | 1.01E-59 | "down-regulated" |
| PRIM2 | 0.57 | 2.03E-58 | "down-regulated" |
| XRCC2 | 0.57 | 1.06E-57 | "down-regulated" |
| TRIM24 | 0.56 | 1.71E-57 | "down-regulated" |
| METTL2B | 0.56 | 3.93E-57 | "down-regulated" |
| PRIM1 | 0.56 | 4.64E-57 | "down-regulated" |
| RALA | 0.56 | 1.16E-56 | "down-regulated" |
| ZNF138 | 0.56 | 2.56E-55 | "down-regulated" |
| SMC2 | 0.55 | 5.22E-55 | "down-regulated" |
| RFC3 | 0.55 | 6.18E-54 | "down-regulated" |
| NUP205 | 0.55 | 3.59E-53 | "down-regulated" |
| MCM4 | 0.55 | 5.04E-53 | "down-regulated" |
| CBLL1 | 0.54 | 6.59E-53 | "down-regulated" |
| SGOL2 | 0.54 | 1.52E-52 | "down-regulated" |
| ZNF12 | 0.54 | 5.34E-52 | "down-regulated" |
| KBTBD2 | 0.54 | 6.67E-51 | "down-regulated" |
| VMA21 | 0.53 | 1.24E-50 | "down-regulated" |
| C7orf44 | 0.53 | 1.85E-50 | "down-regulated" |
| ZNF3 | 0.53 | 2.09E-50 | "down-regulated" |
| RRM1 | 0.53 | 2.68E-50 | "down-regulated" |
| ZNF627 | 0.53 | 2.94E-50 | "down-regulated" |
| KIF15 | 0.53 | 3.33E-50 | "down-regulated" |
| POT1 | 0.53 | 1.82E-49 | "down-regulated" |
| FAM200A | 0.52 | 1.65E-48 | "down-regulated" |
| CENPI | 0.52 | 2.72E-48 | "down-regulated" |
| C7orf60 | 0.52 | 5.55E-48 | "down-regulated" |
| ZNF709 | 0.52 | 8.35E-48 | "down-regulated" |
| CASP2 | 0.52 | 1.12E-47 | "down-regulated" |
| ZNF398 | 0.52 | 1.17E-47 | "down-regulated" |
| EIF2AK1 | 0.52 | 2.31E-47 | "down-regulated" |
| RMI1 | 0.52 | 3.80E-47 | "down-regulated" |
| MAD2L1 | 0.52 | 4.56E-47 | "down-regulated" |
| CCDC15 | 0.52 | 5.36E-47 | "down-regulated" |
| KIF11 | 0.52 | 6.11E-47 | "down-regulated" |
| PURB | 0.52 | 9.92E-47 | "down-regulated" |
| GART | 0.51 | 1.52E-46 | "down-regulated" |
| RBL1 | 0.51 | 1.82E-46 | "down-regulated" |
| LSM5 | 0.51 | 1.98E-46 | "down-regulated" |
| EZH2 | 0.51 | 4.98E-46 | "down-regulated" |
| SP3 | 0.51 | 6.77E-46 | "down-regulated" |
| CDC25A | 0.51 | 7.25E-46 | "down-regulated" |
| GTPBP10 | 0.51 | 1.08E-45 | "down-regulated" |
| NT5C3 | 0.51 | 1.13E-45 | "down-regulated" |
| RAD18 | 0.51 | 5.16E-45 | "down-regulated" |
| C11orf82 | 0.51 | 6.82E-45 | "down-regulated" |
| C4orf46 | 0.51 | 8.87E-45 | "down-regulated" |
| CRCP | 0.51 | 9.02E-45 | "down-regulated" |
| ESCO2 | 0.51 | 1.12E-44 | "down-regulated" |
| ZNF614 | 0.51 | 1.18E-44 | "down-regulated" |
| ZNF680 | 0.50 | 2.31E-44 | "down-regulated" |
| WDR76 | 0.50 | 2.39E-44 | "down-regulated" |
| GINS1 | 0.50 | 3.30E-44 | "down-regulated" |
| UBE2D4 | 0.50 | 3.47E-44 | "down-regulated" |
| MCM7 | 0.50 | 3.68E-44 | "down-regulated" |
| RFWD3 | 0.50 | 1.20E-43 | "down-regulated" |
| ERCC8 | 0.50 | 1.50E-43 | "down-regulated" |
| CENPL | 0.50 | 1.93E-43 | "down-regulated" |
| HNRPLL | 0.50 | 2.51E-43 | "down-regulated" |
| POLD3 | 0.50 | 2.56E-43 | "down-regulated" |
| CCDC138 | 0.50 | 2.94E-43 | "down-regulated" |
| KIAA1524 | 0.50 | 3.11E-43 | "down-regulated" |
| RQCD1 | 0.50 | 3.16E-43 | "down-regulated" |
| SENP1 | 0.50 | 3.50E-43 | "down-regulated" |
| FBXO5 | 0.50 | 5.77E-43 | "down-regulated" |
| C7orf36 | 0.50 | 6.79E-43 | "down-regulated" |
| USP42 | 0.50 | 7.57E-43 | "down-regulated" |
| LMNB1 | 0.50 | 8.33E-43 | "down-regulated" |
| CDCA7 | 0.49 | 1.41E-42 | "down-regulated" |
| FANCD2 | 0.49 | 1.58E-42 | "down-regulated" |
| ZNF498 | 0.49 | 1.62E-42 | "down-regulated" |
| SGOL1 | 0.49 | 1.88E-42 | "down-regulated" |
| CASC5 | 0.49 | 3.18E-42 | "down-regulated" |
| DHX40 | 0.49 | 3.43E-42 | "down-regulated" |
| PHF14 | 0.49 | 5.38E-42 | "down-regulated" |
| SRR | 0.49 | 7.46E-42 | "down-regulated" |
| NUSAP1 | 0.49 | 9.70E-42 | "down-regulated" |
| CDC7 | 0.49 | 1.01E-41 | "down-regulated" |
| METTL2A | 0.49 | 1.28E-41 | "down-regulated" |
| LOC100128191 | 0.49 | 1.66E-41 | "down-regulated" |
| THAP5 | 0.49 | 1.98E-41 | "down-regulated" |
| SKA3 | 0.49 | 2.07E-41 | "down-regulated" |
| ECT2 | 0.49 | 2.32E-41 | "down-regulated" |
| RAD1 | 0.49 | 4.43E-41 | "down-regulated" |
| RBMX | 0.49 | 5.87E-41 | "down-regulated" |
| POLR2D | 0.49 | 6.14E-41 | "down-regulated" |
| CCDC99 | 0.49 | 6.90E-41 | "down-regulated" |
| CENPH | 0.49 | 7.43E-41 | "down-regulated" |
| SMAD5 | 0.49 | 7.52E-41 | "down-regulated" |
| SPC25 | 0.49 | 8.45E-41 | "down-regulated" |
| DTL | 0.48 | 1.43E-40 | "down-regulated" |
| PPM1D | 0.48 | 1.86E-40 | "down-regulated" |
| RAD51AP1 | 0.48 | 1.90E-40 | "down-regulated" |
| NEIL3 | 0.48 | 1.98E-40 | "down-regulated" |
| FANCI | 0.48 | 2.26E-40 | "down-regulated" |
| ZKSCAN5 | 0.48 | 2.46E-40 | "down-regulated" |
| BRIP1 | 0.48 | 2.63E-40 | "down-regulated" |
| TMED4 | 0.48 | 4.14E-40 | "down-regulated" |
| DHFR | 0.48 | 4.22E-40 | "down-regulated" |
| CTDSPL2 | 0.48 | 4.40E-40 | "down-regulated" |
| CKAP2L | 0.48 | 5.36E-40 | "down-regulated" |
| MCM6 | 0.48 | 5.45E-40 | "down-regulated" |
| ERCC6L | 0.48 | 5.70E-40 | "down-regulated" |
| BUB1 | 0.48 | 6.27E-40 | "down-regulated" |
| KIF14 | 0.48 | 6.58E-40 | "down-regulated" |
| ZNF207 | 0.48 | 7.54E-40 | "down-regulated" |
| STIL | 0.48 | 9.08E-40 | "down-regulated" |
| CHCHD3 | 0.48 | 1.03E-39 | "down-regulated" |
| TIMELESS | 0.48 | 1.10E-39 | "down-regulated" |
| CENPO | 0.48 | 1.20E-39 | "down-regulated" |
| MCM2 | 0.48 | 2.97E-39 | "down-regulated" |
| CDC23 | 0.48 | 5.35E-39 | "down-regulated" |
| NUP160 | 0.48 | 5.40E-39 | "down-regulated" |
| PMPCB | 0.48 | 5.76E-39 | "down-regulated" |
| TRIP13 | 0.47 | 6.11E-39 | "down-regulated" |
| RBBP4 | 0.47 | 6.70E-39 | "down-regulated" |
| PRC1 | 0.47 | 9.73E-39 | "down-regulated" |
| OIP5 | 0.47 | 9.80E-39 | "down-regulated" |
| KNTC1 | 0.47 | 1.17E-38 | "down-regulated" |
| POLA1 | 0.47 | 1.17E-38 | "down-regulated" |
| BRCA1 | 0.47 | 1.23E-38 | "down-regulated" |
| TMEM194A | 0.47 | 1.39E-38 | "down-regulated" |
| ATAD5 | 0.47 | 1.47E-38 | "down-regulated" |
| HMMR | 0.47 | 2.58E-38 | "down-regulated" |
| RINT1 | 0.47 | 4.54E-38 | "down-regulated" |
| TOP2A | 0.47 | 5.23E-38 | "down-regulated" |
| RFC4 | 0.47 | 5.42E-38 | "down-regulated" |
| TTK | 0.47 | 5.83E-38 | "down-regulated" |
| ZNF43 | 0.47 | 1.32E-37 | "down-regulated" |
| NUF2 | 0.47 | 1.47E-37 | "down-regulated" |
| KIF18A | 0.47 | 1.67E-37 | "down-regulated" |
| CENPF | 0.47 | 1.68E-37 | "down-regulated" |
| EXO1 | 0.47 | 1.79E-37 | "down-regulated" |
| SKP2 | 0.47 | 2.14E-37 | "down-regulated" |
| C13orf34 | 0.47 | 2.42E-37 | "down-regulated" |
| NCAPH | 0.47 | 2.46E-37 | "down-regulated" |
| PBK | 0.47 | 3.19E-37 | "down-regulated" |
| PLK4 | 0.46 | 5.73E-37 | "down-regulated" |
| UBTD2 | 0.46 | 5.74E-37 | "down-regulated" |
| USP1 | 0.46 | 6.40E-37 | "down-regulated" |
| CHEK1 | 0.46 | 6.48E-37 | "down-regulated" |
| KIF4A | 0.46 | 7.90E-37 | "down-regulated" |
| ZNF480 | 0.46 | 8.44E-37 | "down-regulated" |
| PRR11 | 0.46 | 9.69E-37 | "down-regulated" |
| ZIK1 | 0.46 | 1.00E-36 | "down-regulated" |
| ZNF367 | 0.46 | 1.29E-36 | "down-regulated" |
| CENPQ | 0.46 | 1.40E-36 | "down-regulated" |
| DEPDC1B | 0.46 | 1.47E-36 | "down-regulated" |
| NUP155 | 0.46 | 1.51E-36 | "down-regulated" |
| INTS7 | 0.46 | 1.77E-36 | "down-regulated" |
| TOPBP1 | 0.46 | 2.16E-36 | "down-regulated" |
| BAZ1B | 0.46 | 2.93E-36 | "down-regulated" |
| ZNF93 | 0.46 | 3.18E-36 | "down-regulated" |
| ZNF260 | 0.46 | 3.29E-36 | "down-regulated" |
| C18orf54 | 0.46 | 3.29E-36 | "down-regulated" |
| CDK1 | 0.46 | 3.93E-36 | "down-regulated" |
| ALS2CR4 | 0.46 | 4.19E-36 | "down-regulated" |
| SLC37A3 | 0.46 | 5.35E-36 | "down-regulated" |
| FOXM1 | 0.46 | 5.38E-36 | "down-regulated" |
| GSG2 | 0.46 | 7.58E-36 | "down-regulated" |
| MRPL42 | 0.46 | 7.79E-36 | "down-regulated" |
| ZNF100 | 0.46 | 1.18E-35 | "down-regulated" |
| GTSE1 | 0.46 | 1.26E-35 | "down-regulated" |
| TMEM106B | 0.46 | 1.53E-35 | "down-regulated" |
| BRCA2 | 0.46 | 1.66E-35 | "down-regulated" |
| RFC5 | 0.45 | 1.74E-35 | "down-regulated" |
| WDHD1 | 0.45 | 1.95E-35 | "down-regulated" |
| PAXIP1 | 0.45 | 2.06E-35 | "down-regulated" |
| KIFC1 | 0.45 | 2.81E-35 | "down-regulated" |
| ZNF420 | 0.45 | 3.89E-35 | "down-regulated" |
| CDC25C | 0.45 | 4.05E-35 | "down-regulated" |
| SNX13 | 0.45 | 4.60E-35 | "down-regulated" |
| MTERF | 0.45 | 5.04E-35 | "down-regulated" |
| ZNF273 | 0.45 | 5.10E-35 | "down-regulated" |
| SMC4 | 0.45 | 5.57E-35 | "down-regulated" |
| TPX2 | 0.45 | 5.65E-35 | "down-regulated" |
| MSH2 | 0.45 | 5.86E-35 | "down-regulated" |
| EME1 | 0.45 | 6.02E-35 | "down-regulated" |
| NUP107 | 0.45 | 7.00E-35 | "down-regulated" |
| TYW1 | 0.45 | 9.02E-35 | "down-regulated" |
| RTKN2 | 0.45 | 9.08E-35 | "down-regulated" |
| NCAPD3 | 0.45 | 9.55E-35 | "down-regulated" |
| KIF24 | 0.45 | 9.74E-35 | "down-regulated" |
| ZNF639 | 0.45 | 1.03E-34 | "down-regulated" |
| C2orf44 | 0.45 | 1.37E-34 | "down-regulated" |
| USP39 | 0.45 | 1.43E-34 | "down-regulated" |
| KIF4B | 0.45 | 1.48E-34 | "down-regulated" |
| CDK13 | 0.45 | 1.70E-34 | "down-regulated" |
| ARHGAP11A | 0.45 | 1.96E-34 | "down-regulated" |
| CCNA2 | 0.45 | 1.99E-34 | "down-regulated" |
| MCM10 | 0.45 | 2.37E-34 | "down-regulated" |
| C5orf34 | 0.45 | 2.46E-34 | "down-regulated" |
| MYBL1 | 0.45 | 2.59E-34 | "down-regulated" |
| MCM8 | 0.45 | 2.67E-34 | "down-regulated" |
| CCNB1 | 0.45 | 4.14E-34 | "down-regulated" |
| PHTF2 | 0.45 | 4.17E-34 | "down-regulated" |
| CMTM1 | 0.45 | 5.48E-34 | "down-regulated" |
| ADNP | 0.44 | 8.19E-34 | "down-regulated" |
| SKA1 | 0.44 | 8.50E-34 | "down-regulated" |
| GSTCD | 0.44 | 8.76E-34 | "down-regulated" |
| TRA2B | 0.44 | 8.87E-34 | "down-regulated" |
| AURKA | 0.44 | 1.34E-33 | "down-regulated" |
| SPIN4 | 0.44 | 1.48E-33 | "down-regulated" |
| TCF19 | 0.44 | 2.63E-33 | "down-regulated" |
| CCNE2 | 0.44 | 2.67E-33 | "down-regulated" |
| SUZ12 | 0.44 | 2.73E-33 | "down-regulated" |
| E2F2 | 0.44 | 2.83E-33 | "down-regulated" |
| ZNF670 | 0.44 | 2.90E-33 | "down-regulated" |
| CDCA2 | 0.44 | 3.15E-33 | "down-regulated" |
| FANCC | 0.44 | 3.20E-33 | "down-regulated" |
| ZNF800 | 0.44 | 3.22E-33 | "down-regulated" |
| FANCB | 0.44 | 3.42E-33 | "down-regulated" |
| STARD3NL | 0.44 | 3.42E-33 | "down-regulated" |
| DEK | 0.44 | 3.57E-33 | "down-regulated" |
| C6orf167 | 0.44 | 3.77E-33 | "down-regulated" |
| LUZP6 | 0.44 | 3.83E-33 | "down-regulated" |
| RFC2 | 0.44 | 4.40E-33 | "down-regulated" |
| ASF1B | 0.44 | 6.00E-33 | "down-regulated" |
| C12orf48 | 0.44 | 6.06E-33 | "down-regulated" |
| KIF18B | 0.44 | 6.63E-33 | "down-regulated" |
| VKORC1L1 | 0.44 | 7.54E-33 | "down-regulated" |
| FAM64A | 0.44 | 7.57E-33 | "down-regulated" |
| LMBR1 | 0.44 | 7.62E-33 | "down-regulated" |
| G3BP1 | 0.44 | 8.19E-33 | "down-regulated" |
| HELLS | 0.44 | 8.59E-33 | "down-regulated" |
| SKA2 | 0.44 | 1.02E-32 | "down-regulated" |
| ASPM | 0.44 | 1.04E-32 | "down-regulated" |
| CNOT10 | 0.44 | 1.10E-32 | "down-regulated" |
| XPO1 | 0.44 | 1.11E-32 | "down-regulated" |
| PUS7 | 0.44 | 1.12E-32 | "down-regulated" |
| C17orf80 | 0.44 | 1.20E-32 | "down-regulated" |
| NUPL2 | 0.44 | 1.51E-32 | "down-regulated" |
| ZNF486 | 0.44 | 1.72E-32 | "down-regulated" |
| DBR1 | 0.44 | 1.78E-32 | "down-regulated" |
| NCAPG | 0.44 | 1.95E-32 | "down-regulated" |
| GABPB1 | 0.44 | 1.95E-32 | "down-regulated" |
| MLF1IP | 0.44 | 1.98E-32 | "down-regulated" |
| ZNF322A | 0.44 | 2.00E-32 | "down-regulated" |
| HMGB2 | 0.44 | 2.24E-32 | "down-regulated" |
| PCNA | 0.44 | 2.32E-32 | "down-regulated" |
| DSCC1 | 0.44 | 2.72E-32 | "down-regulated" |
| FAM111B | 0.43 | 3.21E-32 | "down-regulated" |
| TGS1 | 0.43 | 3.58E-32 | "down-regulated" |
| DKC1 | 0.43 | 3.62E-32 | "down-regulated" |
| RNASEH2A | 0.43 | 3.83E-32 | "down-regulated" |
| EPR1 | 0.43 | 4.08E-32 | "down-regulated" |
| C7orf70 | 0.43 | 5.77E-32 | "down-regulated" |
| ZNF136 | 0.43 | 5.97E-32 | "down-regulated" |
| ODC1 | 0.43 | 6.08E-32 | "down-regulated" |
| ZNF675 | 0.43 | 6.54E-32 | "down-regulated" |
| CENPM | 0.43 | 7.00E-32 | "down-regulated" |
| CCNB2 | 0.43 | 7.07E-32 | "down-regulated" |
| RAD51 | 0.43 | 7.70E-32 | "down-regulated" |
| CSE1L | 0.43 | 1.00E-31 | "down-regulated" |
| MCM3 | 0.43 | 1.22E-31 | "down-regulated" |
| ZNF131 | 0.43 | 1.29E-31 | "down-regulated" |
| CENPK | 0.43 | 1.35E-31 | "down-regulated" |
| ZCCHC10 | 0.43 | 1.44E-31 | "down-regulated" |
| MELK | 0.43 | 1.62E-31 | "down-regulated" |
| FAM133B | 0.43 | 1.89E-31 | "down-regulated" |
| ZNF567 | 0.43 | 2.00E-31 | "down-regulated" |
| ZNF253 | 0.43 | 2.22E-31 | "down-regulated" |
| RRM2 | 0.43 | 2.54E-31 | "down-regulated" |
| LOC81691 | 0.43 | 2.78E-31 | "down-regulated" |
| GINS2 | 0.43 | 3.00E-31 | "down-regulated" |
| SIKE1 | 0.43 | 3.03E-31 | "down-regulated" |
| PPPDE1 | 0.43 | 3.11E-31 | "down-regulated" |
| SMARCE1 | 0.43 | 3.83E-31 | "down-regulated" |
| E2F7 | 0.43 | 4.11E-31 | "down-regulated" |
| TRAIP | 0.43 | 4.23E-31 | "down-regulated" |
| CDCA8 | 0.43 | 5.35E-31 | "down-regulated" |
| MIOS | 0.43 | 6.49E-31 | "down-regulated" |
| C12orf41 | 0.43 | 7.33E-31 | "down-regulated" |
| PSMA2 | 0.43 | 8.58E-31 | "down-regulated" |
| NCBP1 | 0.43 | 8.59E-31 | "down-regulated" |
| ORC6L | 0.43 | 8.91E-31 | "down-regulated" |
| ELAVL1 | 0.43 | 9.59E-31 | "down-regulated" |
| RNF2 | 0.42 | 1.35E-30 | "down-regulated" |
| POLA2 | 0.42 | 1.39E-30 | "down-regulated" |
| KIF20A | 0.42 | 1.50E-30 | "down-regulated" |
| POLQ | 0.42 | 1.72E-30 | "down-regulated" |
| CENPA | 0.42 | 1.74E-30 | "down-regulated" |
| PLK1 | 0.42 | 1.78E-30 | "down-regulated" |
| NUP88 | 0.42 | 1.81E-30 | "down-regulated" |
| ZNF562 | 0.42 | 1.90E-30 | "down-regulated" |
| WDR67 | 0.42 | 2.33E-30 | "down-regulated" |
| GAS2L3 | 0.42 | 2.52E-30 | "down-regulated" |
| CREB1 | 0.42 | 2.72E-30 | "down-regulated" |
| C15orf23 | 0.42 | 2.72E-30 | "down-regulated" |
| TMEM68 | 0.42 | 2.88E-30 | "down-regulated" |
| URB2 | 0.42 | 2.97E-30 | "down-regulated" |
| CGGBP1 | 0.42 | 2.99E-30 | "down-regulated" |
| THUMPD3 | 0.42 | 3.22E-30 | "down-regulated" |
| CKS2 | 0.42 | 3.50E-30 | "down-regulated" |
| CCDC126 | 0.42 | 3.66E-30 | "down-regulated" |
| KIF2C | 0.42 | 3.73E-30 | "down-regulated" |
| ZNF558 | 0.42 | 4.49E-30 | "down-regulated" |
| ESPL1 | 0.42 | 4.65E-30 | "down-regulated" |
| HJURP | 0.42 | 4.80E-30 | "down-regulated" |
| TRMT61B | 0.42 | 5.00E-30 | "down-regulated" |
| SPC24 | 0.42 | 5.66E-30 | "down-regulated" |
| CDCA5 | 0.42 | 6.91E-30 | "down-regulated" |
| ZBTB33 | 0.42 | 7.00E-30 | "down-regulated" |
| BUB1B | 0.42 | 7.95E-30 | "down-regulated" |
| TMEM48 | 0.42 | 8.70E-30 | "down-regulated" |
| GTF2IRD2B | 0.42 | 9.29E-30 | "down-regulated" |
| BARD1 | 0.42 | 9.42E-30 | "down-regulated" |
| TSPAN12 | 0.42 | 9.78E-30 | "down-regulated" |
| CENPE | 0.42 | 1.06E-29 | "down-regulated" |
| GGH | 0.42 | 1.10E-29 | "down-regulated" |
| ZNF85 | 0.42 | 1.21E-29 | "down-regulated" |
| DSN1 | 0.42 | 1.31E-29 | "down-regulated" |
| PALB2 | 0.42 | 1.59E-29 | "down-regulated" |
| ZNF682 | 0.42 | 1.63E-29 | "down-regulated" |
| VEZF1 | 0.42 | 1.69E-29 | "down-regulated" |
| NOL11 | 0.42 | 1.77E-29 | "down-regulated" |
| HAUS1 | 0.42 | 1.82E-29 | "down-regulated" |
| ZNF655 | 0.42 | 2.46E-29 | "down-regulated" |
| TBRG4 | 0.42 | 2.74E-29 | "down-regulated" |
| XRCC5 | 0.42 | 2.78E-29 | "down-regulated" |
| COIL | 0.42 | 3.04E-29 | "down-regulated" |
| CDC6 | 0.41 | 3.14E-29 | "down-regulated" |
| TBCCD1 | 0.41 | 3.44E-29 | "down-regulated" |
| NUP35 | 0.41 | 4.14E-29 | "down-regulated" |
| DLGAP5 | 0.41 | 4.43E-29 | "down-regulated" |
| NEK2 | 0.41 | 4.48E-29 | "down-regulated" |
| MYNN | 0.41 | 5.40E-29 | "down-regulated" |
| KIF23 | 0.41 | 5.74E-29 | "down-regulated" |
| E2F8 | 0.41 | 5.93E-29 | "down-regulated" |
| MYBL2 | 0.41 | 6.73E-29 | "down-regulated" |
| DEPDC1 | 0.41 | 6.83E-29 | "down-regulated" |
| NDC80 | 0.41 | 7.68E-29 | "down-regulated" |
| RBBP8 | 0.41 | 8.14E-29 | "down-regulated" |
| POLR1B | 0.41 | 9.36E-29 | "down-regulated" |
| ZNF845 | 0.41 | 9.78E-29 | "down-regulated" |
| CSNK1G3 | 0.41 | 1.11E-28 | "down-regulated" |
| GTF2IP1 | 0.41 | 1.18E-28 | "down-regulated" |
| TROAP | 0.41 | 1.24E-28 | "down-regulated" |
| ACTL6A | 0.41 | 1.38E-28 | "down-regulated" |
| DSEL | 0.41 | 1.38E-28 | "down-regulated" |
| CDKAL1 | 0.41 | 1.42E-28 | "down-regulated" |
| PIGW | 0.41 | 1.72E-28 | "down-regulated" |
| KIAA0101 | 0.41 | 1.93E-28 | "down-regulated" |
| MKI67 | 0.41 | 1.95E-28 | "down-regulated" |
| CLSPN | 0.41 | 2.27E-28 | "down-regulated" |
| CDKN2C | 0.41 | 2.54E-28 | "down-regulated" |
| CDC45 | 0.41 | 2.57E-28 | "down-regulated" |
| CEP76 | 0.41 | 2.61E-28 | "down-regulated" |
| BCDIN3D | 0.41 | 3.04E-28 | "down-regulated" |
| HERPUD2 | 0.41 | 3.09E-28 | "down-regulated" |
| MARS2 | 0.41 | 3.64E-28 | "down-regulated" |
| CPSF3 | 0.41 | 4.88E-28 | "down-regulated" |
| CDK4 | 0.41 | 4.98E-28 | "down-regulated" |
| BIRC5 | 0.41 | 5.00E-28 | "down-regulated" |
| KIAA0406 | 0.41 | 5.17E-28 | "down-regulated" |
| CRY1 | 0.41 | 5.78E-28 | "down-regulated" |
| ZWINT | 0.41 | 6.00E-28 | "down-regulated" |
| ZFP30 | 0.41 | 6.50E-28 | "down-regulated" |
| PKMYT1 | 0.41 | 6.74E-28 | "down-regulated" |
| DPY19L4 | 0.41 | 7.84E-28 | "down-regulated" |
| FKBP14 | 0.41 | 8.09E-28 | "down-regulated" |
| NONO | 0.41 | 8.48E-28 | "down-regulated" |
| ZNF300 | 0.40 | 9.16E-28 | "down-regulated" |
| ORC1L | 0.40 | 9.77E-28 | "down-regulated" |
| PSME3 | 0.40 | 1.25E-27 | "down-regulated" |
| GTF2IRD2 | 0.40 | 1.26E-27 | "down-regulated" |
| IFT81 | 0.40 | 1.44E-27 | "down-regulated" |
| POM121C | 0.40 | 1.48E-27 | "down-regulated" |
| SPAG5 | 0.40 | 1.74E-27 | "down-regulated" |
| RPA1 | 0.40 | 1.89E-27 | "down-regulated" |
| RBM12 | 0.40 | 1.90E-27 | "down-regulated" |
| C5orf24 | 0.40 | 2.18E-27 | "down-regulated" |
| INTS8 | 0.40 | 2.20E-27 | "down-regulated" |
| MSH6 | 0.40 | 2.32E-27 | "down-regulated" |
| TSN | 0.40 | 2.48E-27 | "down-regulated" |
| ZNF599 | 0.40 | 2.99E-27 | "down-regulated" |
| PPP1CC | 0.40 | 3.06E-27 | "down-regulated" |
| DCLRE1B | 0.40 | 3.09E-27 | "down-regulated" |
| ZNF140 | 0.40 | 3.11E-27 | "down-regulated" |
| SMARCD1 | 0.40 | 3.36E-27 | "down-regulated" |
| C15orf42 | 0.40 | 3.49E-27 | "down-regulated" |
| TMSB15B | 0.40 | 3.60E-27 | "down-regulated" |
| C7orf28B | 0.40 | 3.95E-27 | "down-regulated" |
| CAMTA2 | -0.40 | 3.57E-27 | "up-regulated" |
| PQLC1 | -0.40 | 3.08E-27 | "up-regulated" |
| PACS2 | -0.40 | 2.97E-27 | "up-regulated" |
| RILPL1 | -0.40 | 1.34E-27 | "up-regulated" |
| SPRYD3 | -0.40 | 1.20E-27 | "up-regulated" |
| NBL1 | -0.40 | 1.03E-27 | "up-regulated" |
| CBX7 | -0.41 | 2.15E-28 | "up-regulated" |
| SLC25A28 | -0.41 | 1.54E-28 | "up-regulated" |
| PI4K2A | -0.41 | 6.92E-29 | "up-regulated" |
| ZBTB7A | -0.41 | 3.15E-29 | "up-regulated" |
| RPS6KA4 | -0.42 | 2.49E-29 | "up-regulated" |
| AVPI1 | -0.42 | 9.52E-30 | "up-regulated" |
| BIN1 | -0.42 | 8.75E-30 | "up-regulated" |
| DNAJB2 | -0.42 | 3.00E-30 | "up-regulated" |
| MAP7D1 | -0.42 | 2.18E-30 | "up-regulated" |
| CTTN | -0.42 | 1.30E-30 | "up-regulated" |
| LZTS2 | -0.42 | 1.15E-30 | "up-regulated" |
| PNPLA6 | -0.43 | 4.04E-31 | "up-regulated" |
| FKBP8 | -0.43 | 2.25E-31 | "up-regulated" |
| BCL2L2 | -0.43 | 3.91E-32 | "up-regulated" |
| TOM1 | -0.44 | 2.56E-32 | "up-regulated" |
| PAOX | -0.44 | 2.24E-32 | "up-regulated" |
| C22orf9 | -0.44 | 2.00E-32 | "up-regulated" |
| LARP6 | -0.45 | 3.53E-34 | "up-regulated" |
| BAIAP2 | -0.45 | 5.64E-35 | "up-regulated" |
| SETD3 | -0.45 | 3.78E-35 | "up-regulated" |
| MAPK3 | -0.46 | 2.99E-36 | "up-regulated" |
| ZFYVE28 | -0.47 | 9.04E-38 | "up-regulated" |

**Supplementary Table S5. List of primers used in this study.**

| **Gene** |  |  | **Primer Sequences (5’to 3’)** |
| --- | --- | --- | --- |
| ACTB | F |  | CACCAACTGGGACGACAT |
|  | R |  | ACAGCCTGGATAGCAACG |
| HSP70 | F |  | ATGTCGGTGGTGGGCATAGA |
|  | R |  | CACAGCGACGTAGCAGCTCT |
| TBP | F |  | CGGTTTGCTGCGGTAATCAT |
|  | R |  | TTTCTTGCTGCCAGTCTGGAC |
| H2AZ2 | F |  | CAAGGCTAAGGCAGTATCTCG |
|  | R |  | GGTACTCCAGAATCGCAGCAC |
| MACROH2A1 | F |  | CGGATGCTGCGGTACATCAA |
|  | R |  | CTCCGCTGTCAGGTATTCCAG |
| H2AX | F |  | GGCCTTTCACATCAGCTCTC |
|  | R |  | ATTGCCGAGTTGAGTTTGCT |
| H2AZ1 | F |  | GGCGGTAAGGCTGGAAAGG |
|  | R |  | TGTCGATGAATACGGCCCAC |
| OLIG2 | F |  | GGTAAGTGCGCAATGCTAAGCTGT |
|  | R |  | TACAAAGCCCAGTTTGCAACGCAG |
| FABP7 | F |  | GCACATTCAAGAACACGGAGA |
|  | R |  | CACATCACCAAAAGTAAGGGTCA |
| PLK1 | F |  | CCTGCACCGAAACCGAGTTAT |
|  | R |  | CCGTCATATTCGACTTTGGTTGC |
| CDC20 | F |  | GACCACTCCTAGCAAACCTGG |
|  | R |  | GGGCGTCTGGCTGTTTTCA |
| MELK | F |  | TATTCACCTCGATGATGATTGCG |
|  | R |  | AGAAAGCCTTAAACGAACTGGTT |
| CDK2 | F |  | CCAGGAGTTACTTCTATGCCTGA |
|  | R |  | TTCATCCAGGGGAGGTACAAC |
| AURKB | F |  | CGCAGAGAGATCGAAATCCAG |
|  | R |  | AGATCCTCCTCCGGTCATAAAA |
| KIF18B | F |  | GCTGCAAGTAGTGGTACGGG |
|  | R |  | CCTCAGGGTTAAACACCAGCA |
| E2F1 | F |  | ACGCTATGAGACCTCACTGAA |
|  | R |  | TCCTGGGTCAACCCCTCAAG |
| H2AZ2_STAT3 ChIP-qPCR | F |  | TGGGAAGGACAGCAAAATTAGC |
|  | R |  | ACAAGTCTCAGAAGCGGGAA |
| H2AZ2_E2F1 ChIP-qPCR | F |  | GCCTCCTTCTAGCCAGACC |
|  | R |  | AGAGAGCGCGTCACTATTGG |
| c-FOS_STAT3 ChIP-qPCR | F |  | GCAGCCCGCGAGCAGTT |
|  | R |  | GCCTTGGCGCGTGTCCTAATC |
