# Supplementary material for: E2F and STAT3 provide transcriptional synergy for histone variant H2AZ activation to sustain glioblastoma chromatin accessibility and tumorigenicity
Source: Cell Death Differ. 2022 Jan 20;29(7):1379–94. doi: 10.1038/s41418-021-00926-5 (PMC9287453; doi:10.1038/s41418-021-00926-5)
Supplement: Supplementary file 2 — Supplementary Figure Legend [file 41418_2021_926_MOESM2_ESM.docx]

**SUPPLEMENTARY INFORMATION**

E2F and STAT3 provide transcriptional synergy for histone variant H2AZ activation to sustain glioblastoma chromatin accessibility and tumorigenicity

Jeehyun Yoon^1,2†^, Oleg V. Grinchuk^1,2†^, Roberto Tirado Magallanes^3†^, Zhen Kai Ngian^4^, Emmy Xue Yun Tay^1,2^, You Heng Chuah^1,2^, Bernice Woon Li Lee^1,2^, Jia Feng^1,2^, Karen Carmelina Crasta^1,2,5,6^, Chin Tong Ong^4,7^, Touati Benoukraf^3,8^, Derrick Sek Tong Ong^1,2,6,9^*

**Supplementary figure legends**

**Supplementary Fig. S1. High expression of the *H2AZ* isoforms correlates with GBM and GSC stemness. (A)** qRT-PCR analysis of H2A variant histone mRNA levels in GSCs with or without differentiation (N=3) (mean ± SD). TATA box binding protein gene (*TBP*), heat shock protein 70 gene (*HSP70*) and beta-actin gene (*ACTB*) serve as the housekeeping genes; while *OLIG2* serves as the positive control. **(B-G)** Correlative analysis of *H2AZ1* and *H2AZ2* mRNA levels with various common GBM genotypes in TCGA GBM. Mann-Whitney test. **(H and I)** Correlative analysis of *H2AZ2* and *H2AZ1* mRNA levels with GSC marker expression in GBM cells based on CD133 and NES levels (mean ± SD). **(J)** Comparison of *H2AZ2* mRNA levels in GSCs compared to bulk tumors. **(A),** **(H), and (I)** two-tailed unpaired Student’s *t*-test, *: p<0.05, **: p<1.0e-3; ***: p<1.0e-4. **(B-G) and (J)**: Mann-Whitney test.

**Supplementary Fig. S2. *H2AZ2* depletion reduces GSC colony formation and sensitizes GSC to carboplatin. (A)** qRT-PCR analysis of *H2AZ2* mRNA levels of GSCs with or without *H2AZ2* KD (N=3) (mean ± SD). The housekeeping genes include *TBP*, *HSP70* and *ACTB*. **(B)** Western blot analysis of H2AZ in GSCs, with or without *H2AZ2* KD. β-actin serves as the loading control. **(C)** Soft agar colony formation of *H2AZ2* depleted GSCs (N=4) (mean ± SD). Representative images shown below. **(D)** Cell viability assay of GSCs, with or without *H2AZ2* KD, upon treatment with carboplatin (3 days treatment) (n=6) (mean ± SD). **(E)** Western blot analysis of cleaved-caspase 3 protein levels in GSCs, with or without *H2AZ2* KD, upon treatment with carboplatin (3 days treatment). β-actin serves as the loading control. Two-tailed unpaired Student’s *t*-test. **(A and C)** *: p<1.0e-5. **(D)** *: p<0.005, **: p<10e-4, ***p<10e-8.

**Supplementary Fig. S3. Chromatin accessibility analysis unveils E2F1 as a transcriptional activator of *H2AZ2*.** (**A**) Pie chart with the distribution of the genomic features associated with H2AZ ChIP-Seq peaks in GSC. **(B)** Barplot displaying the log_2_ enrichment for the genomic features associated with ChIP-Seq binding regions of H2AZ. **(C)** Heatmaps displaying binding for H2AZ, H3K4me3 and H3K27ac histone marks in proximity of downregulated ATAC-Seq peaks after *H2AZ2* KD. (**D**) Pie charts with the distribution of the genomic features associated with decreased and increased ATAC-Seq peaks upon *H2AZ2* KD in GSC. **(E)** Integrative analysis of ATAC-Seq as well as H3K27ac, H2AZ and E2F1 ChIP-seq peaks at the *H2AZ2* proximal promoter. **(F)** Western blot analysis of E2F1 and H2AZ protein levels in *E2F1* KD GSC, with or without *H2AZ2* overexpression. Vinculin serves as a loading control. **(G)** Soft agar colony formation of *E2F1* KD GSCs, with or without *H2AZ2* overexpression (N=4) (mean ± SD). (**H**) Representative images of (**G**). Two-tailed unpaired Student’s *t*-test, ***p<0.01.

**Supplementary Fig. S4. Transcriptomic analysis of *H2AZ2* depleted GSC. (A)** qRT-PCR validation of representative cell cycle genes that were downregulated in *H2AZ2* KD GSC. *: p<0.05. **(B)** Cell cycle analysis of GSCs with or without *H2AZ2* KD. *: p<0.05; **: p<0.001; ***: p<1.0E-04. **(C and D)** Quantification and representative images of BrdU^+^ GSC upon *H2AZ2* KD (N=over 300 nuclei per sample). Scale bar, 10µm. *: p<0.05. **(E)** Venn diagram showing the 99 high confidence H2AZ2-associated gene targets that were also downregulated upon *H2AZ2* KD in GSC. **(F)** Venn diagram showing the 99 high confidence H2AZ2-associated gene targets with reduced transcriptional output that harbor E2F1 and/or STAT3 ChIP-Seq peaks.

**Supplementary Fig. S5. A chemical biology approach identified STAT3 as another *H2AZ2* transcriptional activator. (A)** Results of CMA analysis using an “anti-*H2AZ2*” query signature. **(B)** Heatmap showing the expression of genes from the “anti-*H2AZ2*” query signature upon treatment of GSC with 75 μM S3I-201 (3 days). **(C)** GSC cell viability analysis after 3 day treatment with the respective drugs. Two-tailed unpaired Student’s *t*-test, ***p<10e-6. **(D)** Western blot analysis of HA-E2F1 in the STAT3 immunoprecipitates from 293T cell lysates that have STAT3 and HA-E2F1 overexpressed. **(E)** Western blot analysis of endogenous E2F1 in the STAT3 immunoprecipitates from GSC lysates.

**Supplementary Fig. S6. Proposed model of how H2AZ2 regulates the GSC-critical genes. (A)** In *H2AZ2* expressing GSC, H2AZ2 facilitates the accessibility of E2F1 and STAT3 to the enhancer regions within promoters of *H2AZ2* and other E2F1 and STAT3 targets, thereby promoting GSC proliferation/ self-renewal, invasiveness and tumorigenicity. **(B)** In the absence of H2AZ2 protein, there is reduced accessibility of E2F1 and STAT3 to their target genes due to chromatin compaction at the enhancer regions, leading to the downregulation of GSC proliferation and invasiveness genes. **(C)** Co-inhibition of E2F and STAT3 results in the downregulation of their target genes, including *H2AZ2*. Decreased *H2AZ2* transcription in turn reduces H2AZ2 protein level that regulates H3K27ac level and hence cell cycle gene transcription (which is a secondary effect of E2F1/STAT3 co-inhibition), thus mimicking *H2AZ2* KD.
