# Supplementary figures and images for: E2F and STAT3 provide transcriptional synergy for histone variant H2AZ activation to sustain glioblastoma chromatin accessibility and tumorigenicity
Source: Cell Death Differ. 2022 Jan 20;29(7):1379–94. doi: 10.1038/s41418-021-00926-5 (PMC9287453; doi:10.1038/s41418-021-00926-5)

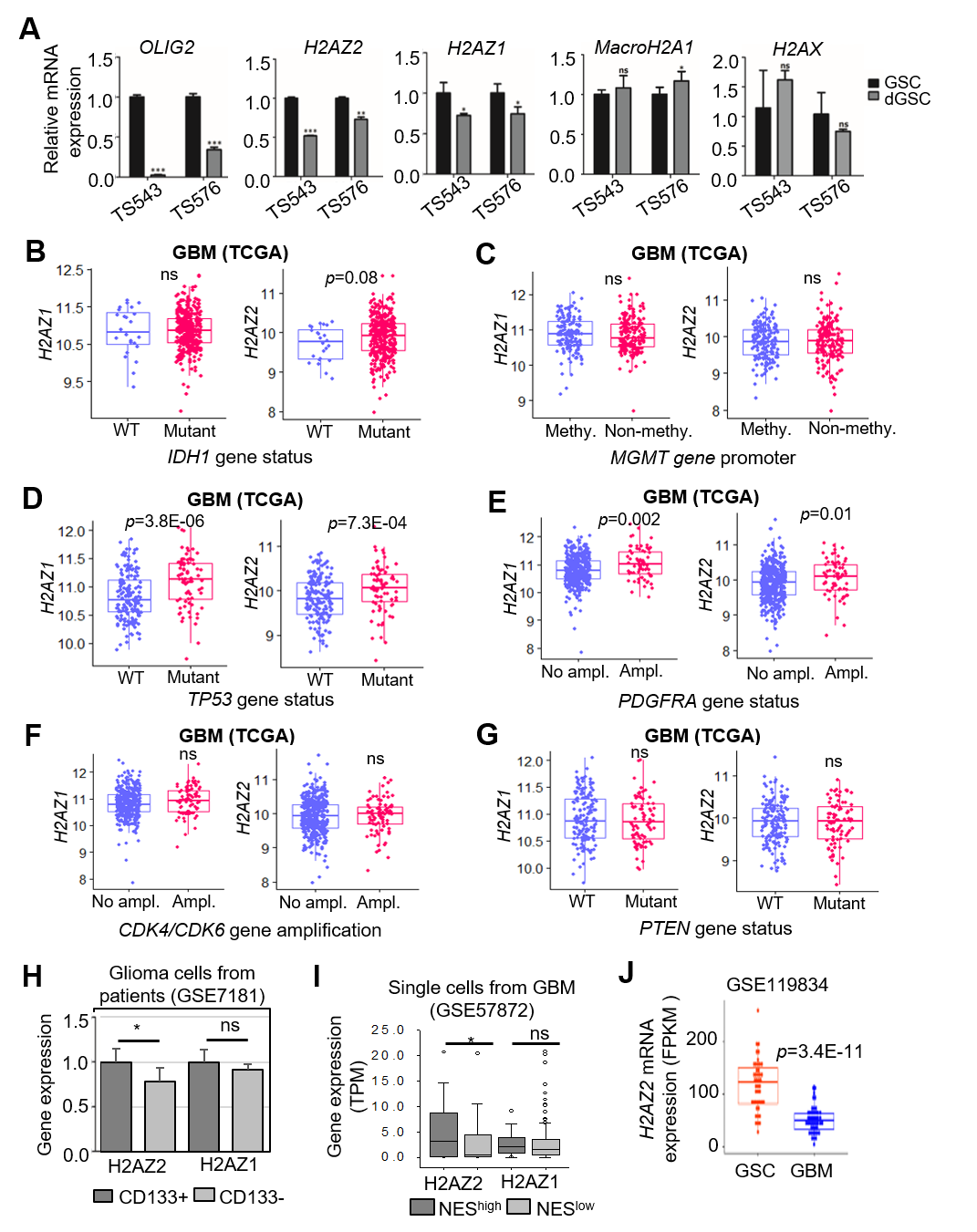

Supplement: Supplementary file 3 — Supplementary Figure 1 [file 41418_2021_926_MOESM3_ESM.tif]

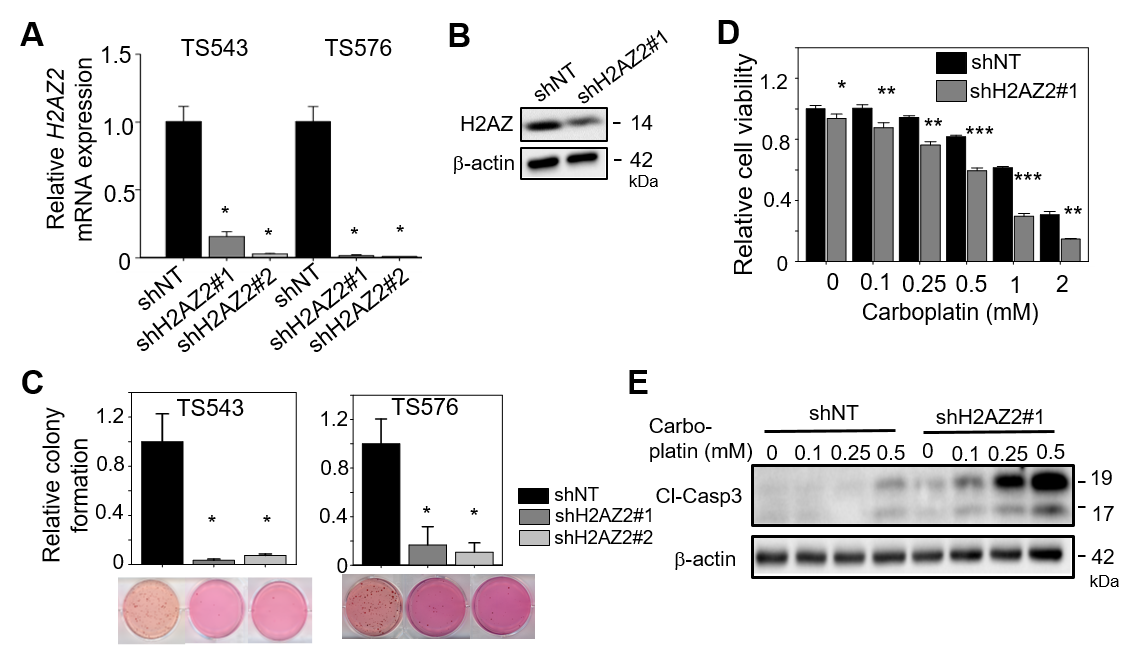

Supplement: Supplementary file 4 — Supplementary Figure 2 [file 41418_2021_926_MOESM4_ESM.tif]

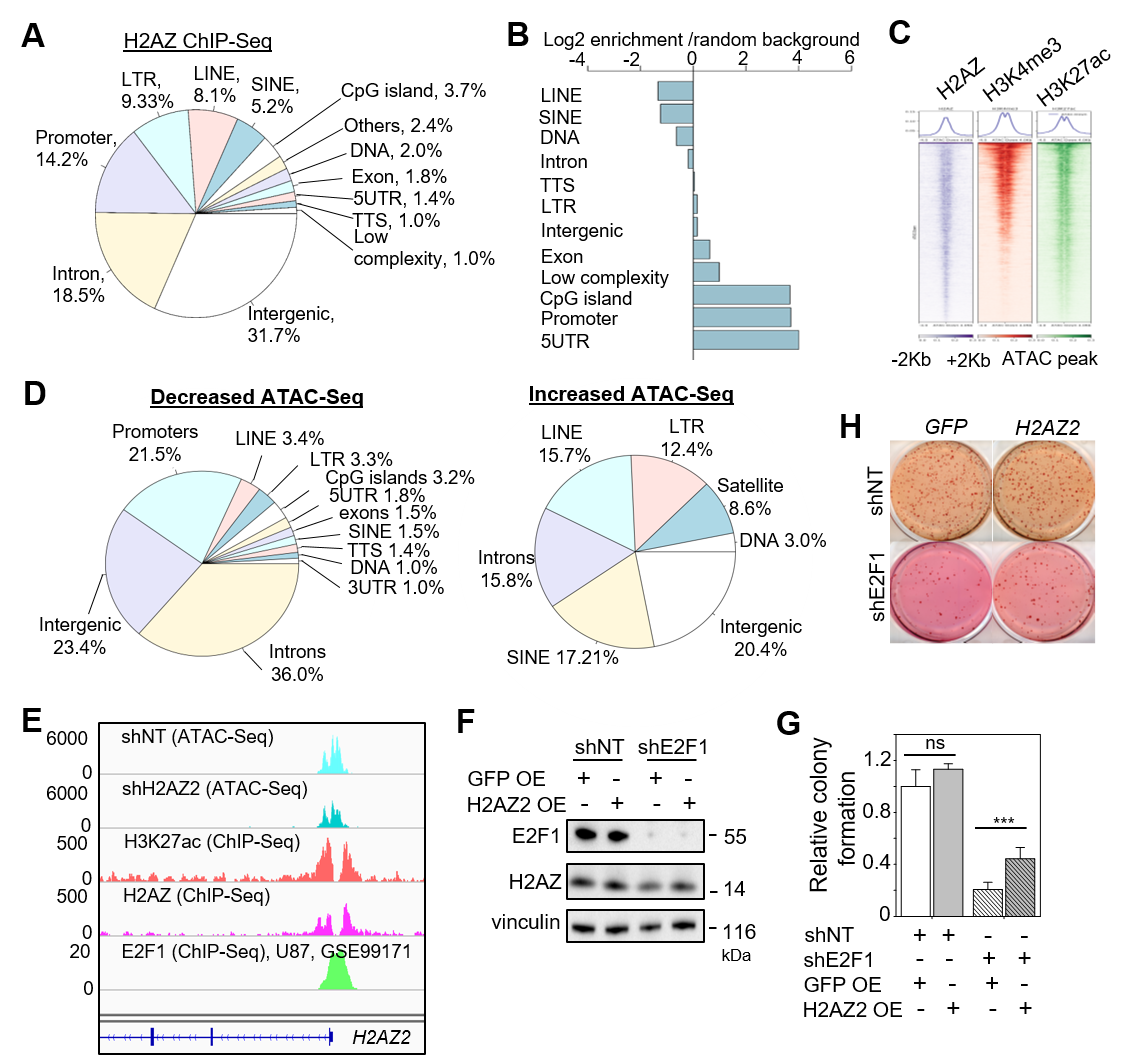

Supplement: Supplementary file 5 — Supplementary Figure 3 [file 41418_2021_926_MOESM5_ESM.tif]

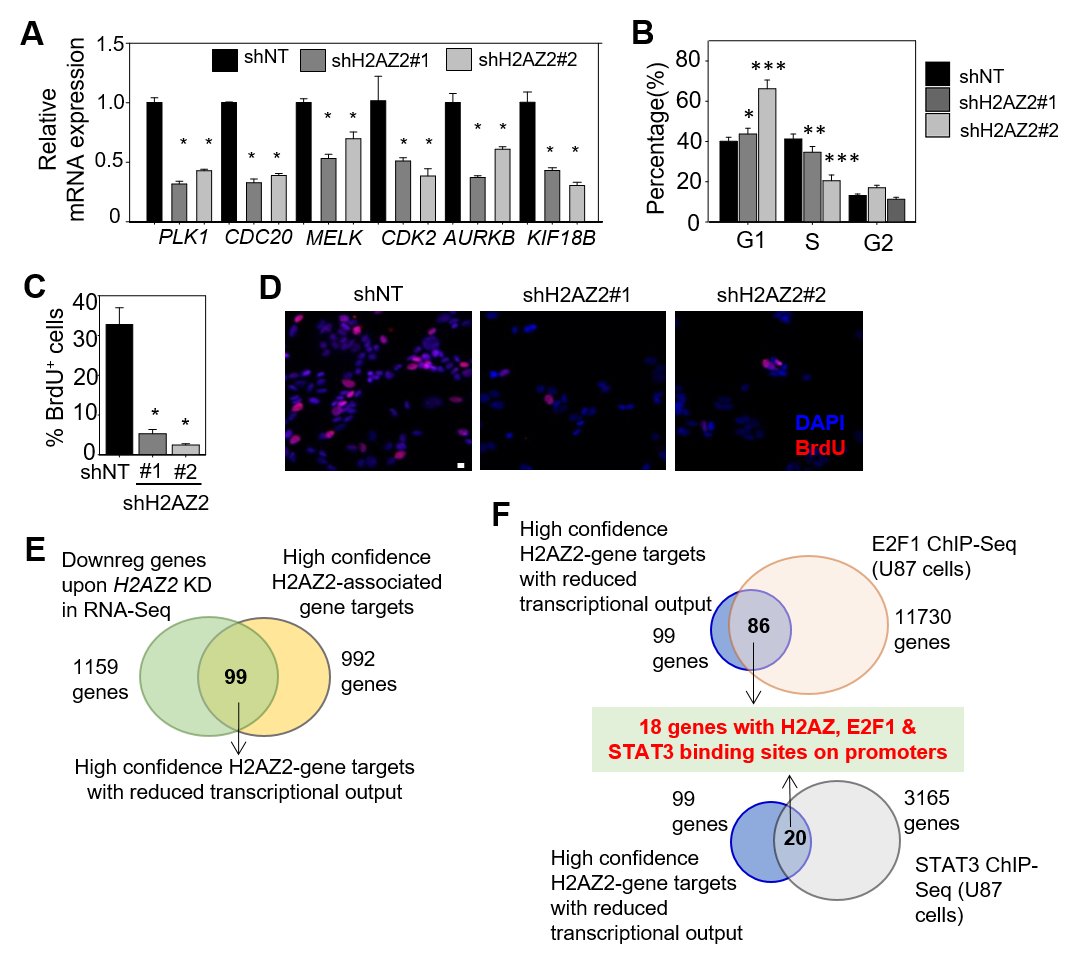

Supplement: Supplementary file 6 — Supplementary Figure 4 [file 41418_2021_926_MOESM6_ESM.tif]

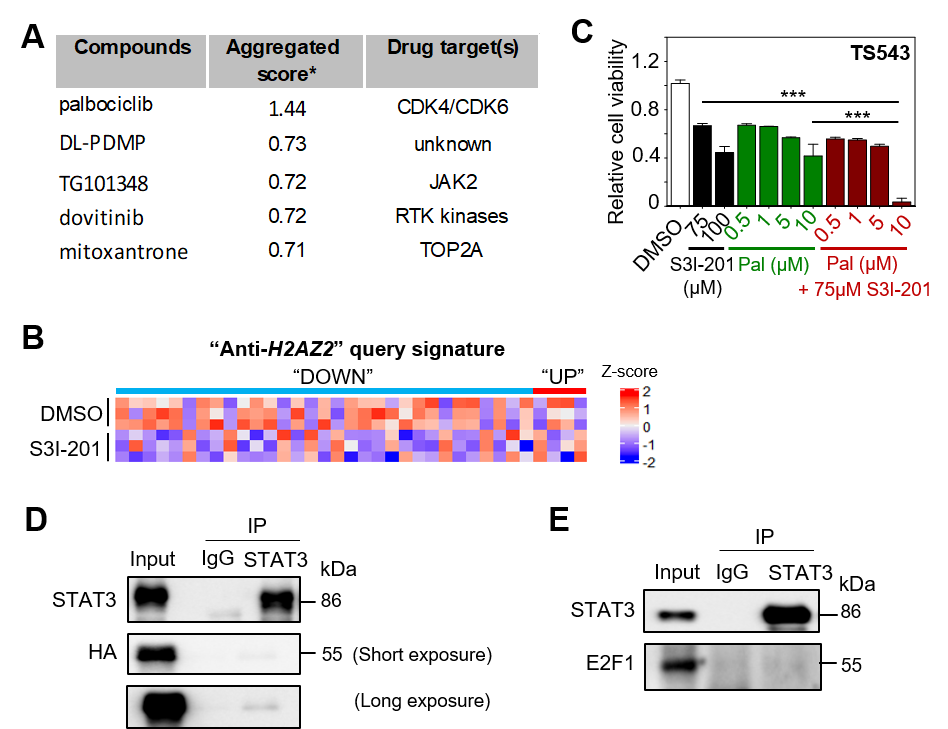

Supplement: Supplementary file 7 — Supplementary Figure 5 [file 41418_2021_926_MOESM7_ESM.tif]

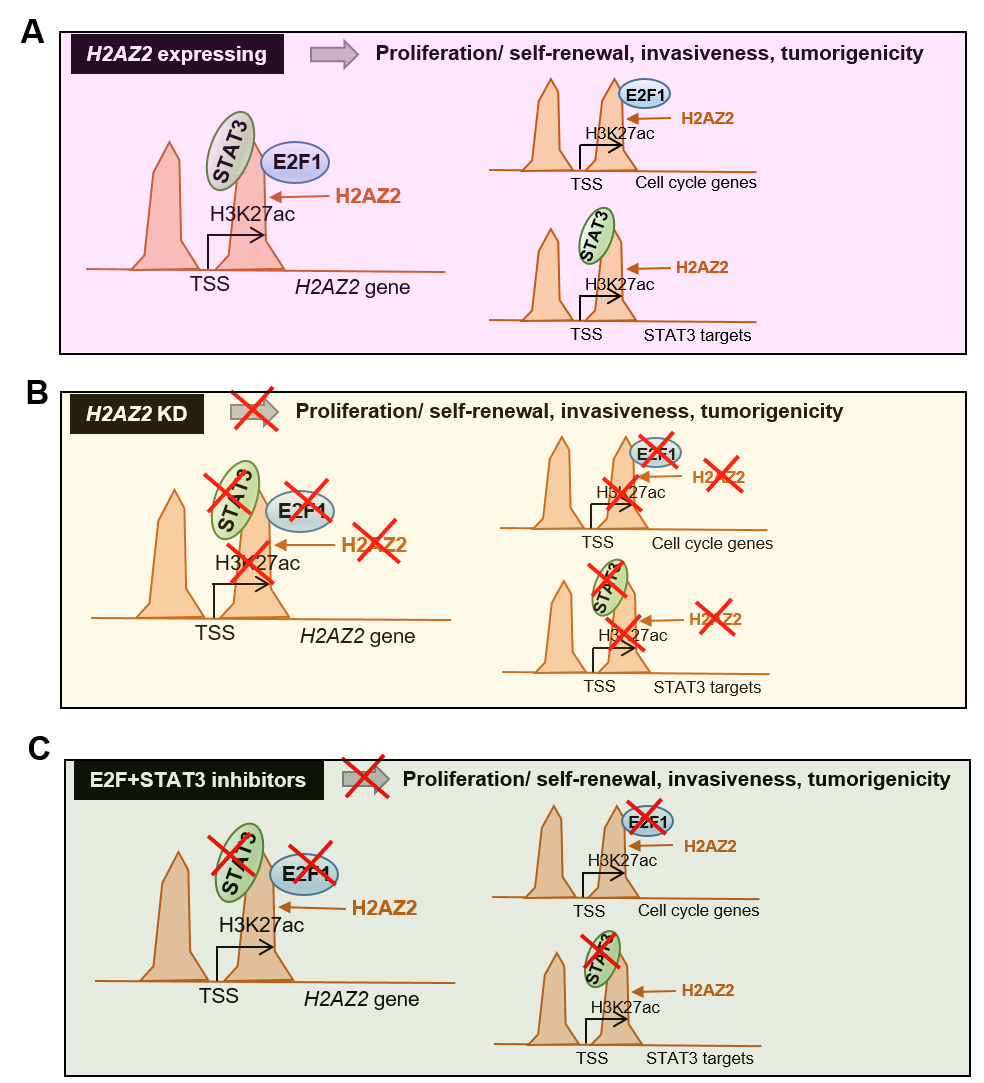

Supplement: Supplementary file 8 — Supplementary Figure 6 [file 41418_2021_926_MOESM8_ESM.tif]
